# Ultrahigh-Throughput Directed Evolution of a Metal-Free α/β-Hydrolase with a Cys-His-Asp Triad into an Efficient Phosphotriesterase

**DOI:** 10.1021/jacs.2c10673

**Published:** 2022-12-30

**Authors:** J. David Schnettler, Oskar James Klein, Tomasz S. Kaminski, Pierre-Yves Colin, Florian Hollfelder

**Affiliations:** Department of Biochemistry, University of Cambridge, 80 Tennis Court Road, Cambridge CB2 1GA, United Kingdom

## Abstract

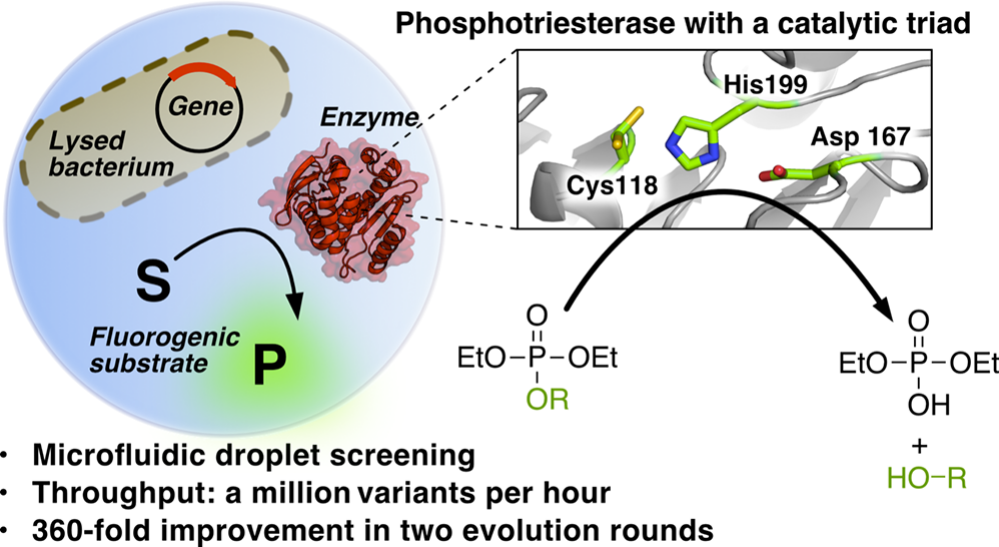

Finding new mechanistic solutions for biocatalytic challenges is key in the evolutionary adaptation of enzymes, as well as in devising new catalysts. The recent release of man-made substances into the environment provides a dynamic testing ground for observing biocatalytic innovation at play. Phosphate triesters, used as pesticides, have only recently been introduced into the environment, where they have no natural counterpart. Enzymes have rapidly evolved to hydrolyze phosphate triesters in response to this challenge, converging onto the same mechanistic solution, which requires bivalent cations as a cofactor for catalysis. In contrast, the previously identified metagenomic promiscuous hydrolase P91, a homologue of acetylcholinesterase, achieves slow phosphotriester hydrolysis mediated by a metal-independent Cys-His-Asp triad. Here, we probe the evolvability of this new catalytic motif by subjecting P91 to directed evolution. By combining a focused library approach with the ultrahigh throughput of droplet microfluidics, we increase P91’s activity by a factor of ≈360 (to a *k*_cat_/*K*_M_ of ≈7 × 10^5^ M^–1^ s^–1^) in only two rounds of evolution, rivaling the catalytic efficiencies of naturally evolved, metal-dependent phosphotriesterases. Unlike its homologue acetylcholinesterase, P91 does not suffer suicide inhibition; instead, fast dephosphorylation rates make the formation of the covalent adduct rather than its hydrolysis rate-limiting. This step is improved by directed evolution, with intermediate formation accelerated by 2 orders of magnitude. Combining focused, combinatorial libraries with the ultrahigh throughput of droplet microfluidics can be leveraged to identify and enhance mechanistic strategies that have not reached high efficiency in nature, resulting in alternative reagents with novel catalytic machineries.

## Introduction

Natural adaptive enzyme evolution is triggered by environmental challenges that need to be overcome with the help of a biocatalyst. Organophosphate nerve agents are a case in point: they range among the most toxic synthetic substances known, acting as potent covalent inhibitors of the enzyme acetylcholinesterase in human synapses and derailing synaptic transmission of nerve signals, which leads to paralysis by seizures and subsequent death by respiratory arrest. This toxic potential has been exploited by their use as chemical warfare agents (e.g., sarin, VX, or novichok). In addition, the global use of phosphotriesters as pesticides in agriculture leads to thousands of hospital admissions with organophosphate poisonings every year. However, the current medical treatment of organophosphate damage is limited to mitigating primary symptoms.^1−3^ From an evolutionary point of view, the massive release of this xenobiotic substance class into the environment (only starting in 1947)^4^ provides a unique opportunity to observe the emergence of new biocatalytic solutions to ecological challenges. While existing organophosphate-degrading enzymes hold prophylactic and therapeutic promise as catalytic bioscavengers,^5−7^ insight into the origins and evolution of further organophosphate-degrading enzymes could broaden the palette of available starting points for the development of suitable bioremediators and therapeutics.^8^

It is well established that new enzymatic activities can arise from pre-existing promiscuous activities that give a head start to adaptive evolution.^9,10^ Here too, in an astonishing showcase of rapid convergent evolution, existing enzymes have, merely within decades, adapted to the efficient hydrolysis of organophosphates. These new phosphotriesterases have arisen from diverse protein superfamilies, such as the amidohydrolases,^11^ the pita-bread fold,^12^ the β-propellers,^13,14^ the metallo-β-lactamases,^15^ and the cyclase family.^16^ Remarkably, all of these enzymes have, despite their diverse evolutionary origins, independently converged on the same mechanistic solution, requiring bivalent cations (Zn^2+^, Ca^2+^, Mn^2+^, Co^2+^) as a cofactor for metal-ion catalysis.^17^ Several promiscuous enzymes with this metal-dependent catalytic motif have also been evolved or engineered to high phosphotriesterase activities.^18−20^ In each case, the emerging new activity relied on the intrinsic promiscuous reactivity of a metal ion that provides Lewis acid catalysis, transition-state charge compensation, and coordination of a water molecule with reduced p*K*_a_ to facilitate its use as a nucleophile in hydrolysis. Other metallohydrolases, e.g., members of the alkaline phosphatase superfamily possess a promiscuous phosphotriester activity, but there is no evidence for the adaptive evolution of this relatively weak side activity.^21,22^

**Figure 1:**
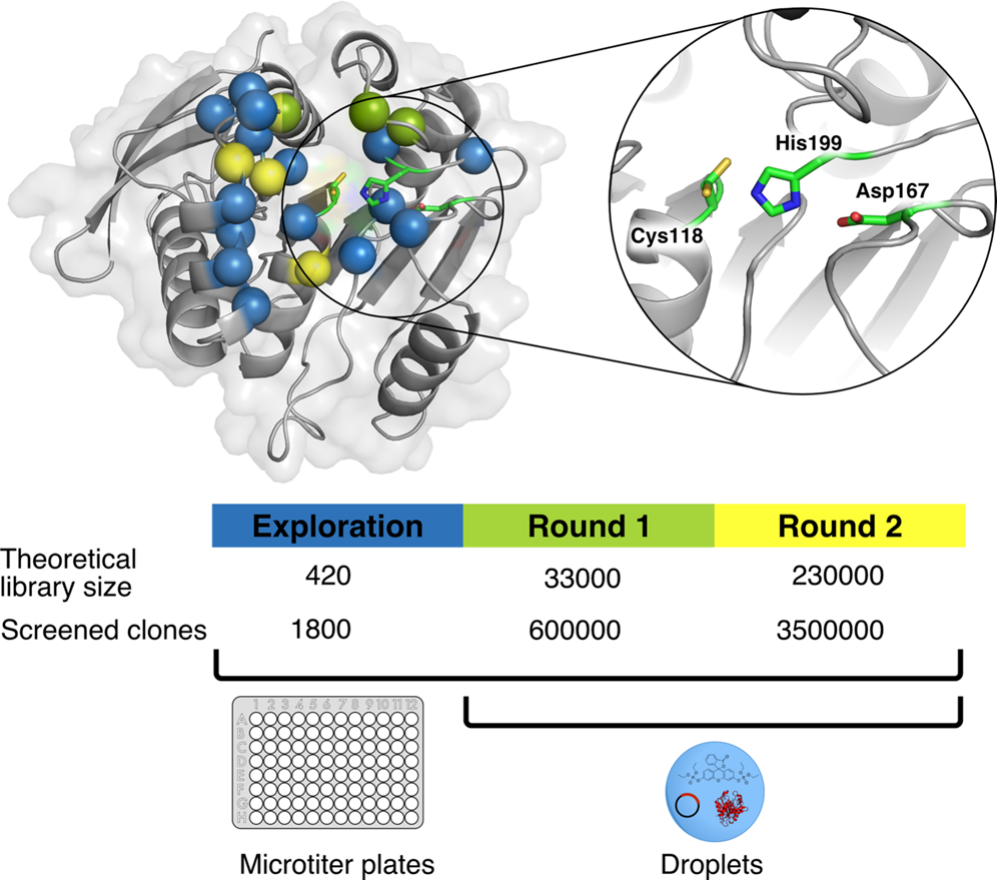
Library strategy for the directed evolution of P91. The active site of P91 was first mutationally explored (all spheres) by the screening of single-site saturation libraries for phosphotriesterase activity in multititer plates (see Figure S2). A subset of these residues was then combined in round 1 (green spheres) and round 2 (yellow spheres) into combinatorial multiple-site saturation mutagenesis libraries, which were screened in microfluidic droplets. In round 1, droplets were incubated off-chip, whereas in round 2, droplets were incubated in a delay line on-chip to maintain selection stringency by shortening the reaction time. **Blowout**: Catalytic triad of P91, consisting of Cys118, His199, and Asp167. Note the two conformations of Cys118 in the structure, an inward-pointing protected and an outward-pointing active conformation (for details on cysteine conformations, see Note S1.18 in the Supporting Information).

Our previous work^23^ had identified a second, metal cofactor-free catalytic motif by microfluidic droplet screening of a >10^6^-membered naïve metagenomic library generated from soil, marine sludge, and cow rumen samples that had not been exposed to phosphotriester contamination. This functional metagenomic screen elicited P91, a member of the α/β hydrolase superfamily and a distant homologue of acetylcholinesterase, with a Cys-His-Asp triad as the catalytic motif (Figure 1).^23^ This superfamily had previously never been associated with proficient phosphotriesterase activity. Quite the opposite was true: α/β hydrolases are inactivated by phosphotriesters that have been specifically designed to target and covalently inhibit the active site nucleophile of their catalytic triads (e. g., the Ser-His-Glu triad of synaptic acetylcholinesterase).^24^

In this context, P91’s ability to hydrolyze phosphotriesters, albeit at low level, was surprising. This observation also posed the question of whether P91’s catalytic triad with a cysteine nucleophile—a new catalytic motif for this substrate—holds the potential for proficient phosphotriester hydrolysis. At the level of chemical reactivity, active sites bearing a cysteine-containing catalytic triad have the potential for evolution: P–S bonds are more labile to hydrolysis compared to P–O bonds,^25^ giving P91 with its cysteine triad an intrinsic advantage over homologous serine triad enzymes in breaking up the covalent intermediate formed upon reaction with phosphotriesters. If not rapidly broken down, multiple turnover is precluded and intermediate formation is effectively inhibitory.

However, there is so far no evidence for the evolution of such a metal-free hydrolase with a cysteine triad into a phosphotriesterase in nature. A promiscuous starting activity does not ensure that a path to high proficiency emerges, either because reaching high rates is intrinsically difficult, given the chemical repertoire of the active site, or because there simply has not been time and biological opportunity to develop the new activity. Here, we subject P91 to directed evolution by microfluidic droplet screening (throughput >10^6^ variants/h) and identify improved phosphotriesterases that illustrate an evolutionary trajectory not observed in nature, elucidating key features necessary for introducing increased phosphotriesterase activity into esterases with a catalytic triad.

## Results

### Mutational Scanning Identifies the Catalytic Potential of Residues near P91’s Active Site

**Figure 2:**
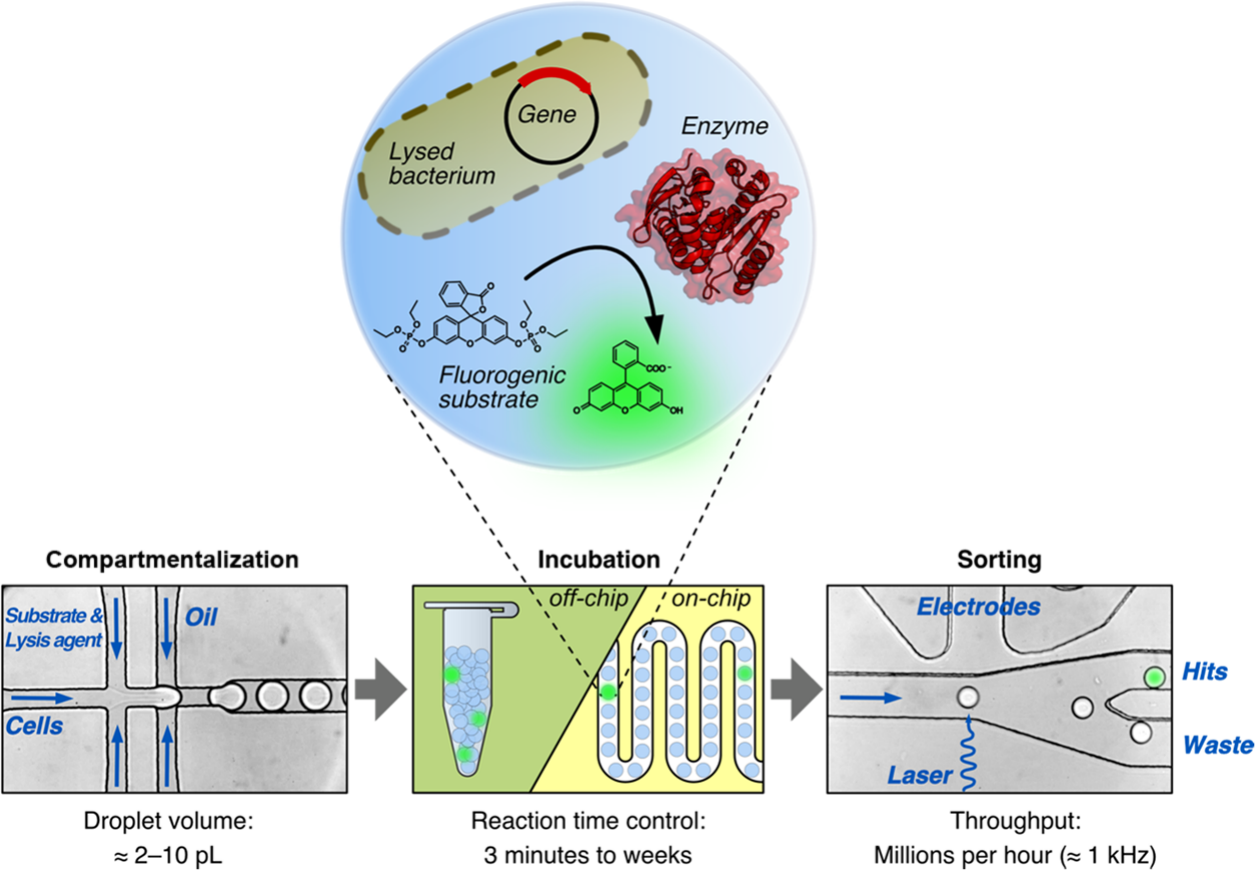
Microfluidic droplet screening assay. The microfluidic screening assay consists of three steps. (1) Encapsulation of bacterial cells together with a fluorogenic substrate and lysis agent into picoliter-sized aqueous droplets that are separated with fluorinated oil. The droplets serve as miniaturized reaction vessels that link phenotype (catalytic activity indicated by fluorescence) to genotype (gene sequence encoded on a plasmid). (2) Droplet incubation can be carried out in a delay line on-chip (for the range of minutes) or off-chip (for hours to weeks). (3) The droplets can then be sorted according to their fluorescence with an excitation laser that is focused on the droplet flow along a Y-shaped junction. When surpassing a preset fluorescence threshold, a single droplet can be electrophoretically deviated by the electrodes away from the waste channel into the hit collection channel. Additional chip details are given in Figures S3 and S5.

To remodel P91’s active site for the proficient turnover of phosphotriesters, we first investigated how substitutions in P91’s active site would affect activity in a ‘mutational scanning’ approach. The protein mutability landscape concept^26^ and our previous work^27^ suggest that designing a relatively small library which is amenable to plate screening can help to explore catalytic potential. All 23 residues that comprise the first shell around the catalytic triad, lining the catalytic site within up to 12 Å of the active site nucleophile Cys118, were individually completely randomized (Figure 1). We determined the maximal increase in lysate activity upon mutation and ranked the residues according to the observed effects (Figure S2). Based on screening for hydrolytic activity toward the fluorogenic model phosphotriester substrate **1** (fluorescein di(diethylphosphate), FDDEP; Figures 2 and S1) we identified three positions most amenable to significant rate enhancements: Ala73, Ile211, and Leu214 (each increasing activity up to ≈8–10-fold in cell lysate). These three residues are in direct vicinity with each other and line the upper edge of the active site (Figure 1, green spheres). They are situated in loops which are partly covering the active site, highly variable in sequence and length within the dienelactone hydrolase-like protein family, to which P91 is assigned based on sequence homology. Considering their crucial roles for substrate specificity and substrate-induced activation,^28−31^ we selected these residues for simultaneous randomization and constructed a plasmid-bound combinatorial library (P91-A) with a theoretical size of ≈33 000 members (32^3^; NNK codes for 32 codons, three positions randomized) using the degenerate codon NNK and transformed it into *Escherichia coli* cells.

### Droplet Screening of P91 Variants Leads to a 360-fold Improvement in Two Rounds

We screened the resulting library for phosphotriesterase activity using ultrahigh-throughput droplet sorting. To this end, the library was expressed in *E. coli* cells which were encapsulated together with substrate and lysis agent in picoliter-sized microdroplet compartments on a microfluidic chip (Figures 2 and S3a). After 2–3 h of incubation, the droplets were re-injected into a separate sorting chip (Figure S3b), where they were screened and sorted according to their fluorescence at kHz frequencies. Due to the high throughput, the entire library could be confidently sampled (≈3.6-fold oversampling at 20% droplet occupancy) by screening ≈600 000 droplets in less than 1 h. Of these, ≈10 000 droplets were selected, corresponding to the top ≈1.7% of brightest droplets. These relatively permissive sorting conditions were chosen to avoid losing false negative clones due to phenotypic variation in enzyme expression on the single cell level, allowing cumulative enrichment of improved clones over several rounds of sorting. This enrichment process by droplet sorting was carried out three times in total to gradually narrow down the diversity of the library before proceeding to secondary screening in microtiter plates. To exert selective pressure on *k*_cat_/*K*_M_, we chose a low substrate concentration of 3 μM (≈1/10 *K*_M_).

Following droplet screening, ≈70 randomly picked individual clones from each sorting (≈350 clones for the third, final sorting) were analyzed in a lysate-based microtiter plate screening, revealing a successive enrichment of active clones along the course of sorting (Figure S4). The most active clones were sequenced, revealing that all sequenced clones had a tryptophan in position 211 and a valine in position 214. Position 73, in contrast, was more varied among enriched variants.

Therefore, we constructed a library (P91-B) for the next round of evolution based on the consensus sequence Trp211, Val214 (dubbed P91-R1) and kept position 73, which had shown no clear consensus, randomized. We additionally randomized three further residues ranking next in terms of activity change upon individual mutation (Ala38, Leu76, and Ala122, Figure S2). Ala38 and Leu76 are in direct vicinity to the residues already randomized in the first library, seaming the active site, and therefore have a high potential of interacting with these. In analogy to the canonical esterase mechanism of P91’s homologue dienelactone hydrolase,^32^ Ala38 contributes with its backbone amide to stabilization of the oxyanion and forms an “oxyanion hole”. Ala122 is directly below the active site nucleophile and was hypothesized to be involved in positioning the hydrolytic water molecule for the breakdown of the covalent intermediate. We randomized these four positions using a mixture of the degenerate codons NDT, VHG, and TGG, which code for all canonical amino acids at approximately even representation and no stop codon (known as 22-codon trick^33^), yielding a theoretical diversity of 160 000 (20^4^) on the amino acid level and ≈234 000 (22^4^) on the nucleotide level.

To adjust for the shorter reaction time required for the improvement of mutants of P91-R1, we designed an integrated device on which droplet generation, incubation, and sorting were combined on a single chip^34,35^ (Figure S5) so that stringent sorting was possible in the second round. On this chip, the incubation time of the droplets was precisely controlled by the length of the delay line.^36^ To optimize the length of the delay line for stringent sorting, we measured the reaction progress of the starting variant P91-R1 in a chip with an elongated delay line (Figures S5a and S6a). The required delay line length for the library sorting device was then chosen such that sorting would occur in the early linear phase of the reaction, corresponding to a reaction time of only 4.5 min (Figures S5b and S6b).

As in the first round, droplet sorting was again repeated three times in total to gradually enrich active clones. In each sorting step, ≈1.5–3.5 million droplets were screened (≈5-fold oversampling of the library), and ≈10 000–70 000 droplets were sorted. To balance throughput with accuracy, the average droplet occupancy was continuously reduced from 35% in the first sorting to 20% in the second and then to 10% in the last sorting. Droplet sorting was again followed by a secondary microtiter plate screening (≈350 randomly picked clones) and sequencing of the most active clones.

**Figure 3:**
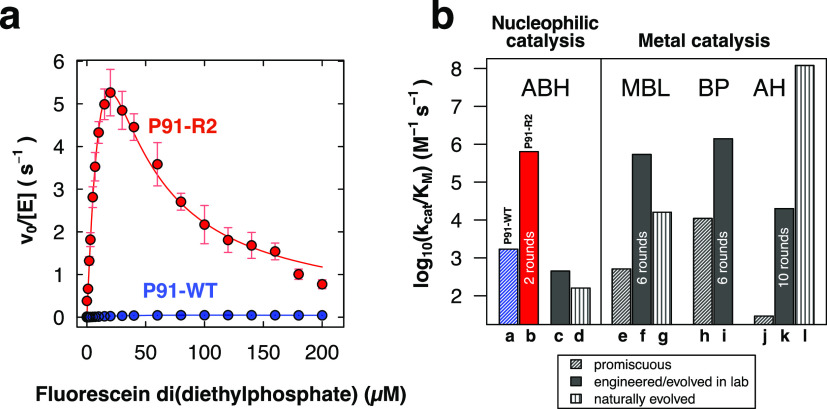
Directed evolution of P91 brings about the mutant P91-R2 that reaches the catalytic efficiencies (*k*_cat_/*K*_M_) of many engineered and naturally evolved metal-dependent phosphotriesterases. (a) Michaelis–Menten plots for P91-WT (blue) and the evolved variant P91-R2 (red) for the hydrolysis of fluorescein di(diethylphosphate) (FDDEP, 1), measured in 50 mM *N*-(2-hydroxyethyl)piperazine-*N*′-ethanesulfonic acid (HEPES)–NaOH, 150 mM NaCl, 1 mM tris(2-carboxyethyl)phosphine (TCEP), pH 8.0 at 25 °C. *v*_0_, initial reaction velocity; [E], initial enzyme concentration. Enzyme concentration was 0.2 μM for P91-WT and 0.2 nM for P91-R2. Error bars represent the standard error of three measurements of separate enzyme purifications. (b) Comparison of catalytic efficiencies of promiscuous (hashed), engineered (filled), and naturally evolved (lined) phosphotriesterases from different protein superfamilies: ABH, α/β-hydrolases; BP, β-propellers; MBL, metallo-β-lactamases; AH, amidohydrolases. P91-WT is shown in blue, and the evolved variant P91-R2 is in red. For enzymes that were evolved by directed evolution, the number of rounds is indicated in the bar. Annotation for the bar labels a–l and the respective references are detailed in Table S2. Substrates of the enzymes shown differ in their leaving groups (*p*-nitrophenol, fluorescein, and umbelliferone) but are all diethyl-substituted phosphotriesters. These are among the most common organophosphate insecticides in agricultural use and, due to their high accessibility compared to highly regulated warfare agents, are used in most published studies dealing with kinetic enzyme characterizations and directed evolution studies. When evolved variants showed higher activity toward a different organophosphate substrate used in the respective study, this is additionally noted in Table S2.

The most improved variant, P91-R2, has five mutations compared to wild-type P91: Ala38Leu, Ala73Glu, Leu76Val, Ile211Trp, and Leu214Val. The kinetic characterization of purified P91-R2 revealed a ≈360-fold increase in *k*_cat_/*K*_M_ over wild type (Figures 3, S7 and S8a, and Table 1), with *k*_cat_/*K*_M_ ≈ 7 × 10^5^ M^–1^ s^–1^ (Table 1). Despite only two rounds of evolution, departing from a weakly promiscuous starting point, P91-R2’s catalytic parameters fall within the range of many evolved and engineered phosphotriesterases employing a metal-assisted mechanism (Figure 3b), a range shared with many physiological enzymes shaped by long-term natural Darwinian evolution.^37^ Although the evolved variant shows a greater propensity for substrate inhibition than the wild type, its total turnover number, even at a high substrate concentration (200 μM), is still one order of magnitude higher than that for the wild type (Figure S9).

**Figure 4:**
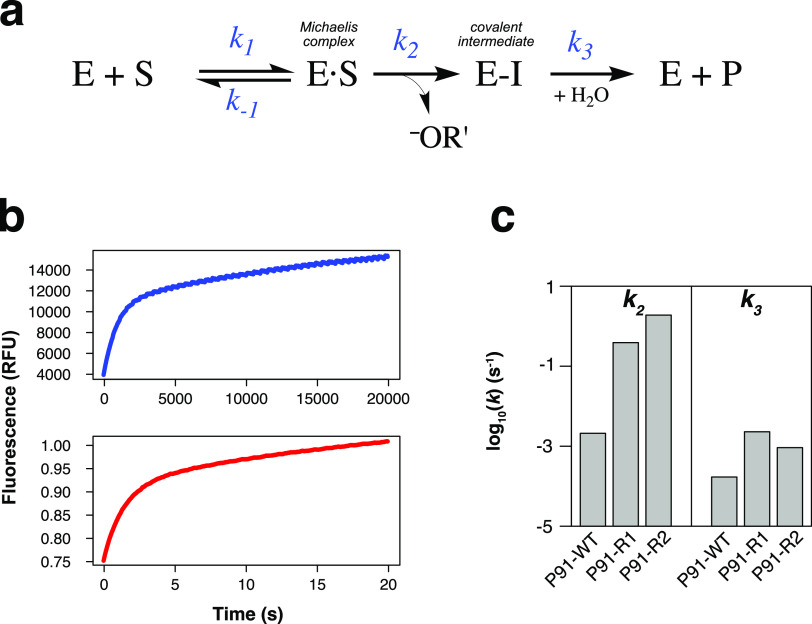
Largest rate improvements by evolution are on intermediate formation. (a) Overview of the reaction scheme postulated in analogy to esterase catalysis by hydrolases with a catalytic triad. P91 (enzyme E) hydrolyzes phosphotriesters (substrate S) via a mechanism involving the formation (phosphorylation, *k*_2_) and breakdown (dephosphorylation, *k*_3_) of a covalent intermediate (E–I). (b) Examples of traces of kinetic bursts with nucleophile-exchanged P91 variants (Cys118Ser). Reaction time course of P91-WT Cys118Ser (blue, enzyme concentration 10 μM) and P91-R2 Cys118Ser (red, enzyme concentration 1 μM) with 100 μM FDDEP in 50 mM HEPES–NaOH, 150 mM NaCl, pH 8.0 at 25 °C. Note the different time scales for the different variants, allowing the use of a spectrophotometric microplate reader for the wild-type-derived enzyme while requiring the use of a stopped-flow instrument for the evolved variant (with each instrument using different relative fluorescence units, RFU). (c) Individual rate constants of nucleophile-exchanged P91 variants (Cys118Ser) were determined from burst kinetics. The phosphorylation rate constant (*k*_2_) was measured with FDDEP (1), and the dephosphorylation rate constant (*k*_3_) was measured with paraoxon-ethyl (2) (see Note S1.15 in the Supporting Information). While *k*_2_ changed by almost 3 orders of magnitude over evolution, *k*_3_ remained approximately within the same order of magnitude.

**Table 1:** Steady-State Catalytic Parameters for Phosphotriester Hydrolysis by His_6_-Tagged P91-WT, P91-R1, and P91-R2 Measured with the Substrate FDDEP^a^

enzyme variant	mutations	*k*_cat_ (s^–1^)^b^	*K*_M_ (μM)^b^	*K*_i_ (μM)	*k*_cat_/*K*_M_ (M^–1^ s^–1^)^c^
P91-WT		0.081 ± 0.014	46 ± 9.7	290 ± 89	(1.8 ± 0.099) × 10^3^
P91-R1	Ile211Trp, Leu214Val	37	120	7.9	3.0 × 10^5^
P91-R2	Ala38Leu, Ala73Glu, Leu76Val, Ile211Trp, Leu214Val	150 ± 120	290 ± 250	7.1 ± 4.2	(6.5 ± 0.88) × 10^5^

a Measured with the substrate FDDEP in 50 mM HEPES–NaOH, 150 mM NaCl, 1 mM TCEP, pH 8.0 at 25 °C. Enzyme concentrations were: 0.2 μM for P91-WT, 1 nM for P91-R1, and 0.2 nM for P91-R2. While for P91-R1, values represent the result of a single measurement, for P91-WT and P91-R2, the indicated values represent the mean and standard error of three biological replicates.

b Due to strong substrate inhibition, estimates of *k*_cat_ and *K*_M_ for P91-R1 and P91-R2 are extrapolations, and only *k*_cat_/*K*_M_ can be regarded as precise (see the Michaelis–Menten plot, Figures S7 and S8a).

c The values measured for P91-WT differ from previously published values (<5-fold),^23^ which may be ascribed to a different affinity tag (here an N-terminal His_6_-tag had replaced an N-terminal StrepII-tag in ref (23)), differences in purification procedure and buffer conditions.

### Nucleophile Exchange and Pre-Steady-State Kinetics Elucidate the Effects of Directed Evolution on a Two-Step Mechanism

To elucidate the cause of P91-R2’s unprecedented activity, we investigated its mechanism. In analogy to the textbook case of serine proteases,^38^ a mechanism that involves a covalent intermediate had previously been postulated for P91 (Figure 4a and Note S1.16 in the Supporting Information on evidence for the presence of a covalent intermediate).^23^ A quantification of the rate constants of formation (*k*_2_) and breakdown (*k*_3_) of the intermediate will allow a comparison with the catalytic triads of other enzymes (e.g., the Ser-His-Glu triad in acetylcholinesterase and many targets beyond),^39^ where a fast and near-irreversible formation of a covalent adduct leads to their inactivation in single-turnover fashion, depriving these enzymes of their physiological function. In the case of this suicide reaction, the phosphorylation rate constant *k*_2_ is much larger than the dephosphorylation rate constant *k*_3_ and *k*_3_ ≈ 0, leading to biphasic kinetics with an observable burst and a flat secondary phase. For a multiple-turnover enzyme, like P91, we expect a larger *k*_3_. However, reaction time courses for P91-WT and its mutants showed monophasic kinetics that could be fitted to a single exponential increase in reaction product (i.e., an initially linear saturation curve), even when measured in a stopped-flow apparatus (Figures S10 and S16). Thus, the rate constants of phosphorylation and dephosphorylation, *k*_2_ and *k*_3_ were not directly measurable due to the lack of an observable burst. A very fast burst within the dead time of the stopped-flow instrument (≈1 ms) can be ruled out by the lack of an increasing signal onset with increasing enzyme concentration (Figure S10). This means that P91, in contrast to acetylcholinesterase, is not rate-limited by the breakdown of its intermediate but by the formation of the covalent adduct (so *k*_2_ < *k*_3_).

A nucleophile exchange from cysteine to serine was then made to alter the rate-determining step by offering an alternative nucleophile with different reactivity. This mutant (Cys118Ser) had 10^2^–10^3^-fold lower second-order rate constants, consistent with the idea that better availability of a deprotonated nucleophile (due to the lower p*K*_a_ of cysteine vs serine) is important for catalysis. Cys118Ser mutants revealed biphasic kinetics, suggesting that now intermediate hydrolysis had become rate-limiting (so now *k*_2_ > *k*_3_). Assuming that the active site nucleophile exchange Cys118Ser affects all variants in the same way, these biphasic kinetics also offer the possibility to quantify the relative effects of the directed evolution campaign on *k*_2_ and *k*_3_. Pre-steady-state burst kinetics could be observed for nucleophile mutants of the wild type (P91-WT Cys118Ser) and of the evolved variant (P91-R2 Cys118Ser) (Figures 4b, S11, and S13) and fitted to a two-step model, describing a fast intermediate formation followed by its slower breakdown, thus allowing quantification of *k*_2_ and *k*_3_ (Figures S12 and S14). Notably, the equipment required to measure rate constants of the initial burst phase (*k*_2_) reflected the large differences between the respective wild-type-based (microtiter plate reader) and the evolved variants (stopped-flow apparatus; Figure 4b). The rate constant of intermediate formation *k*_2_ increased by ≈900-fold from the P91-WT to the evolved P91-R2 background (Figure 4c). *k*_2_ was rate-limiting in the original cysteine-bearing wild-type enzyme, and the similar increase of ≈360-fold in *k*_cat_/*K*_M_ in the evolved mutants suggests that adaptive evolution manifested itself predominantly in faster intermediate formation (thus increasing *k*_2_). This provides a molecular explanation for the observed improvements in the directed evolution experiment and implies that the primary adaptive correction for the promiscuously catalyzed reaction was optimizing the encounter of the enzyme’s nucleophile and the substrate, while the reactivity of the cysteine-bound intermediate was already sufficient to break down rapidly enough to avoid its buildup.

### Specificity Analysis of P91 with the Native Cysteine Triad Confirms a Large Increase in the Rate of Intermediate Formation

To scrutinize whether the changes in rate constants measured in nucleophile-exchanged mutants (Cys118Ser) also applied to the native cysteine enzymes, we additionally analyzed changes in substrate specificity. As changes in transition-state geometry or in the leaving group will affect *k*_2_ and *k*_3_ differently, varying the reaction type and the leaving group would allow us to dissect to which degree those steps were affected by directed evolution. Reaction-type specificity is determined by the enzyme’s adaptation to the transition-state geometry of a reaction and thus affects both *k*_2_ and *k*_3_. Leaving group preference, however, is only evident in the rate constants of formation of the Michaelis complex (*k*_*–*1_/*k*_1_) and nucleophilic attack on the substrate (*k*_2_) without having any effect on *k*_3_ (Figure 4a).

Therefore, we determined arylbutylesterase and phosphotriesterase activity for two different leaving groups, *p*-nitrophenol and fluorescein, respectively (Figures S1 and S8 and Table S1). This was possible because P91-WT has comparable catalytic parameters for the hydrolysis of carboxyesters (with a tetrahedral transition state) in addition to its phosphotriesterase activity (with a trigonal–bipyramidal transition state). P91-WT is a slightly better carboxyesterase than phosphotriesterase for both leaving groups. It also has a slight preference for fluorescein over *p*-nitrophenol as a leaving group.

**Figure 5:**
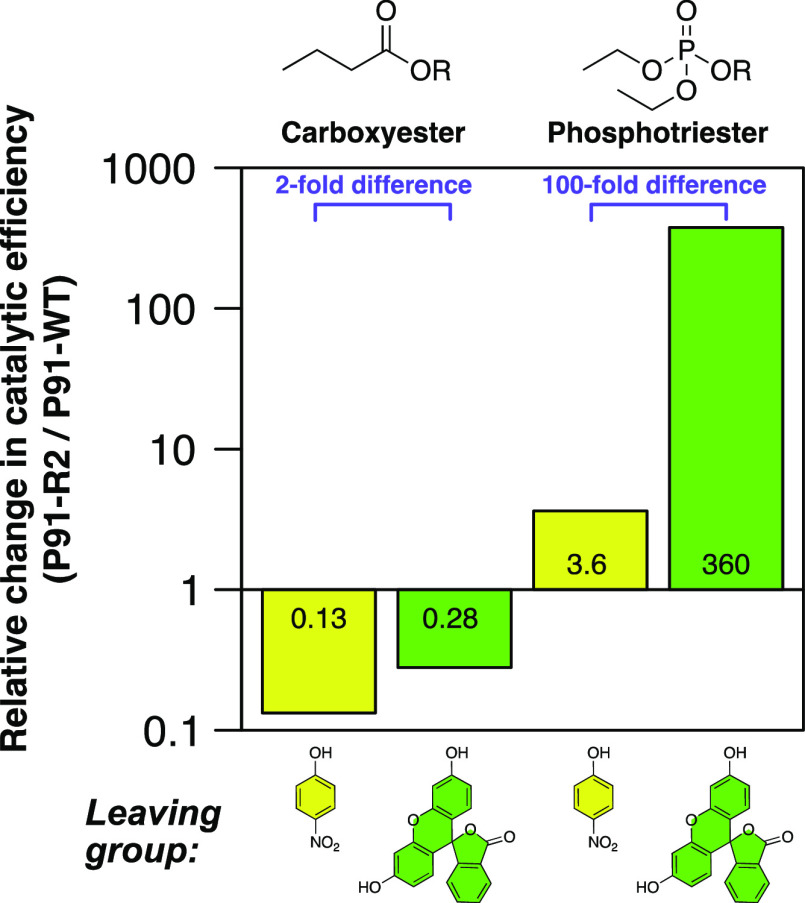
Comparison of reaction-type specificity and leaving group preference for P91-WT and P91-R2 indicates that the main difference is intermediate formation. The relative change in catalytic efficiency (*k*_cat_/*K*_M_) from wild type (P91-WT) to evolved P91 (P91-R2) was measured for carboxyesterase and phosphotriesterase activity with two different leaving groups, *p*-nitrophenol (yellow) and fluorescein (green). Relative changes in catalytic efficiencies were calculated as (*k*_cat_/*K*_M_)_P91-R2_/(*k*_cat_/*K*_M_)_P91-WT_. The *k*_cat_/*K*_M_ values underlying this plot are listed in Table S1.

We observe that over the course of the directed evolution campaign, P91 specializes for both the fluorescein leaving group and for phosphotriester hydrolysis (Figure 5). Remarkably, this specialization toward phosphotriesterase function at the cost of carboxyesterase activity is strongly leaving-group-dependent: the increase in phosphotriesterase activity is about 100-fold more pronounced with fluorescein (≈360-fold increase) than with *p*-nitrophenol (≈3.6-fold increase) as a leaving group. This disparity excludes dephosphorylation (*k*_3_, which is leaving group-independent) as the main improved step, thus validating that the effects observed in the nucleophile-exchanged enzyme variants can be applied to P91 with a cysteine nucleophile. Apparently, the breakdown of the intermediate (*k*_3_) is so fast that it does not need improvement, implying that this step is effectively optimized (compared to an alkoxy leaving group in serine-containing hydrolases). The relative reduction in carboxyesterase activity, however, is not commensurately leaving group-specific (only ≈2-fold difference between leaving groups).

The observation of a preference toward phosphotriesterase activity with both leaving groups suggests that in addition to the recognition of fluorescein, either binding of the phosphotriester moiety in the ground state (i.e., improved substrate binding as the cause of the increased catalytic efficiency) or the phosphotriester transition state (affecting *k*_2_) has been subject to evolution. Both are likely to be involved in the observed behavior. However, as previous rate constant measurements in the nucleophile-exchanged variants were representative of a mechanism in which directed evolution strongly accelerated intermediate formation in P91, the latter may be presumed to be the dominant factor.

### Brønsted Analysis Is Consistent with Rate-Limiting Intermediate Formation

**Figure 6:**
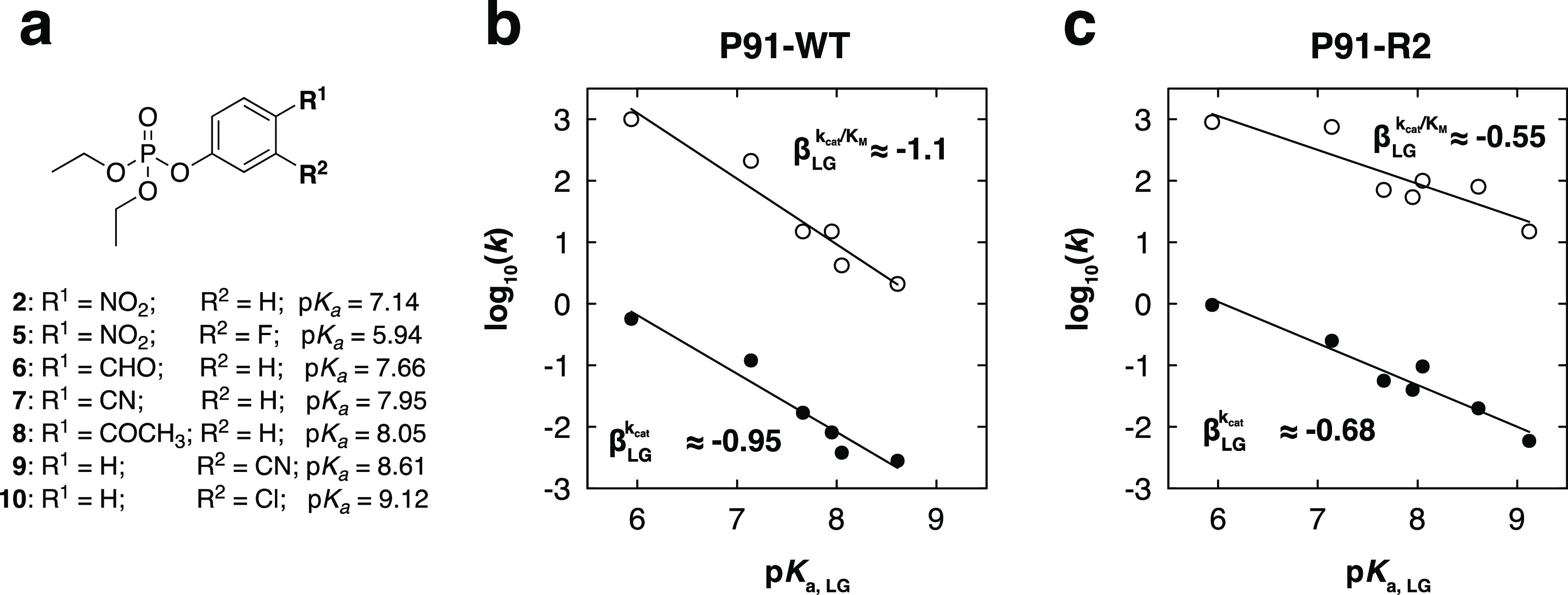
Brønsted analysis shows that the evolved variant P91-R2 accelerates intermediate formation by improved leaving group stabilization. The Brønsted plots show the linear free-energy relationship between the rate constant of hydrolysis of paraoxon derivatives **2** and **5**–**10** (a) and the p*K*_a_ values of their leaving group (also Figure S1 and Table S4) for P91-WT and P91-R2. Filled dots: *k*_cat_ in s^–1^. Open circles: *k*_cat_/*K*_M_ in M^–1^ s^–1^. (b) Brønsted plot for P91-WT: *k*_cat_/*K*_M_: β_LG_ = −1.07, *R*^2^ = 0.93; *k*_cat_: β_LG_ = −0.95, *R*^2^ = 0.94. (c) Brønsted plot for P91-R2: *k*_cat_/*K*_M_: β_LG_ = −0.55, *R*^2^ = 0.80; *k*_cat_: β_LG_ = −0.68, *R*^2^ = 0.94. As the slope of the linear fits (β_LG_) is very similar for both kinetic parameters (*k*_cat_ and *k*_cat_/*K*_M_), intermediate formation (*k*_2_) must be the rate-limiting step. The lower β_LG_ of P91-R2 (≈−0.6) compared to P91-WT (≈−1) indicates that the evolved variant has adapted to offset the charge which accumulates on the leaving group during the transition state. Details on the underlying kinetic measurements can be found in Figure S15 and Tables S5 and S6.

A linear free-energy relationship (LFER) was constructed to quantify the effect of leaving group ability (p*K*_a_, covering the range from 5.9 to 9.1) on the catalytic parameters *k*_cat_ and *k*_cat_/*K*_M_ for a series of paraoxon derivatives (Figures 6a, S1, and S15, and Tables S4–S6).

The Brønsted coefficients β_LG_ were very similar to each other for both Michaelis–Menten parameters, *k*_cat_ and *k*_cat_/*K*_M_, (in both enzymes). Any significant rate-determining influence of the intermediate hydrolysis (*k*_3_) is expected to be independent of leaving group p*K*_a_ and would result in diverging effects on *k*_cat_ and *k*_cat_/*K*_M_ (eqs 5 and 6), as they represent different elemental processes.^40^ The identical effects of leaving group p*K*_a_ on each parameter are consistent with the formation of the covalent intermediate as the rate-limiting step of the reaction and also consistent with the previous conclusions from pre-steady-state kinetics (absence of a burst) and the specificity change analysis (Figure 5).

For *k*_cat_/*K*_M_, this treatment reports on the transition state in the first irreversible step of the reaction, the formation of the intermediate. For P91-WT, we observed a linear relationship, with a slope (β_LG_ ≈ −0.95 and −1.1, respectively; Figure 6b) that was as steep as the uncatalyzed reaction (β_LG_ ≈ −1.0 for spontaneous hydrolysis)^41^ or steeper (0.3 to −0.6 for the hydroxide-catalyzed reaction^41^ and −0.51 with phenolate as a nucleophile),^42^ indicating a substantial charge accumulation on the leaving group during the transition state. The charge compensation by active site groups has been shown to address the challenge of leaving group departure by developing the enzyme’s ability to offset the charge. This charge compensation in the active site leads to shallower Brønsted plots, as previously observed in the evolution^43^ (and also retrospectively by alanine scanning mutations)^44^ in the active site of a sulfatase member of the alkaline phosphatase superfamily. The 360-fold efficiency increase in the evolved variant P91-R2 is likewise accompanied by a shallower Brønsted slope (β_LG_ for *k*_cat_/*K*_M_ ≈ −0.55, for *k*_cat_ ≈ 0.68, Figure 6c). This difference suggests that the leaving group charge offset is at least partially responsible for the acceleration of intermediate formation in P91-R2. This leaving group stabilization leads to a smaller effective change in the transition-state charge at the leaving group oxygen in the enzyme active site (compared to aqueous solution) as the result of improvements after adaptive evolution. The difference in slopes thus reports on the presence or absence of efficient general acid catalysis.

We note that the metal-catalyzed enzymatic reactions in PON1^45^ and *Bd*PTE^46^ were associated with much steeper relationships (β_LG_ ≈ −1.6 and −1.84, respectively), suggesting an entirely different scenario, possibly with a different, larger β_EQ_ (as in protein-free metal complexes)^47^ or more nucleophilic involvement (compared to P91’s thiolate) with little charge compensation on the leaving group.

## Discussion

### Droplet Microfluidics Allow Exploration of New Mechanistic Terrain

Five mutations were sufficient to increase P91’s phosphotriesterase activity by a factor of ≈360, increasing its catalytic efficiency *k*_cat_/*K*_M_ close to 7 × 10^5^ M^–1^ s^–1^ and its turnover rate constant *k*_cat_ into the range of 10–100 s^–1^ (Table 1), although the latter involves a large extrapolation (because substrate inhibition was observed well below the saturation limit in the Michaelis–Menten curve). P91-R2 shows a greater propensity for substrate inhibition than P91-WT, a likely consequence of adaptation to the low substrate concentrations (3 μM) used during screening.

This is the first instance of a catalytic triad turning over organophosphates at high efficiencies and defines a cysteine triad as an evolvable motif for phosphotriesterase activity. As this catalytic motif has no precedent for this reaction and no database sequences with identical functionality exist, prediction of point mutants based on phylogeny is impossible in this case. In the absence of other cysteine-containing catalytic triads in evolved metal-free phosphotriesterases, no sequence comparisons or mechanistic information were available—a knowledge deficit that could be overcome by the focused combinatorial library design combined with the high screening capacity of microfluidic droplet sorting. Screening several orders of magnitude more mutants than possible in multititer plate-based screenings allowed leaps in sequence space, giving access to and establishing new mechanistic territory that, despite strong selective pressure, has not been exploited in nature. Indeed, this work has achieved one of the highest single-round improvements in catalytic efficiency obtained by directed evolution in microfluidic droplets (only surpassed by a single droplet evolution campaign of an oxidase),^34^ underlining the utility of droplet screening. The precision of control over reaction time obtained by switching between off-chip and on-chip droplet incubation allowed flexible fine-tuning of selection stringency over a large range of catalytic efficiencies. Interestingly, the mutations in variant P91-R2 do not correspond to the individually *best* mutations at the respective positions (i.e., their catalytic contributions are not additive). Thus, the combination of mutations in P91-R2 would not have emerged from iterative saturation mutagenesis of single residues (see Figure S19). By contrast, the capacity of droplet screening could accommodate multiple simultaneous mutations in one experiment, tapping the effect of cooperative, epistatic interactions.

### Its Cysteine Triad Predisposes P91 for Promiscuous Phosphotriesterase Activity that Is Evolvable to High Efficiency

Our pre-steady-state measurements are consistent with a covalent mechanism of phosphotriester hydrolysis reminiscent of serine proteases, involving the formation of an intermediate via the cysteine of the catalytic Cys-His-Asp triad. However, in contrast to serine triad enzymes, we find that P91 is already predisposed for fast dephosphorylation (i.e., breakdown of the covalent intermediate) and instead rate-limited by the initial nucleophilic attack on the substrate. This is consistent with a more labile cysteine-connected thiophosphate intermediate compared to a covalent adduct via serine (dissociation energies: P–O bond vs P–S bond: 589 vs 442 kJ/mol),^48^ which leads to the formation of a lower energy P–O bond by subsequent hydrolysis. In energetic terms, introducing a cysteine as a nucleophile instead of a serine increases the ground-state energy of the intermediate thiophosphate and thus lowers the barrier to hydrolyzing it. This increased propensity of the P–S bond for hydrolysis (compared to the P–O bond) in phosphate triesters is well established.^25^ Thus, P91’s predisposition for high dephosphorylation rates can be explained by the intrinsic reactivity of its cysteine triad. Directed evolution then found a solution for the remaining catalytic problem, the rate-limiting nucleophilic attack on the substrate. Indeed, when comparing thiolate (as in a cysteine) and alkoxide (as in a serine) as nucleophiles toward phosphorous, studies on phosphodiesters show that the rate of thiolate attack is 10^7^-fold slower than the attack of the corresponding alkoxide.^49^ This is consistent with our observation that the rate-limiting step in P91 is the initial attack of the cysteine nucleophile on the phosphorous center. The residue changes identified in this directed evolution campaign address the formation of the intermediate, and consequently, the evolved variant P91-R2 achieves its higher efficiency over the wild type by accelerating the initial formation of the intermediate (*k*_2_).

The fact that *k*_cat_ was obtained by extrapolation, and that *K*_M_ is also affected by the curve fit led us to use rate constant comparisons for *k*_cat_/*K*_M_ (see Note S1.14 in the Supporting Information). This second-order regime encompasses the substrate concentration where the screening was carried out ([S]_0_ = 3 μM) and comparisons are unencumbered by substrate inhibition. The measures for rate accelerations that involve comparisons of the catalytic efficiency (*k*_cat_/*K*_M_) are large: (*k*_cat_/*K*_M_)/*k*_uncat_ (catalytic proficiency) is 1.5 × 10^13^ M^–1^ and (*k*_cat_/*K*_M_)/*k*_w_ (second-order rate enhancement) is 8.1 × 10^14^ (based on *k*_uncat_ = 4.4 × 10^–8^ s^–1^ as reported for paraoxon and assuming a similar *k*_uncat_ for FDDEP).^23^ These values suggest substantial transition-state stabilization.

P91-R2 shows a greater propensity for substrate inhibition than P91-WT, possibly a consequence of adaptation to the low substrate concentrations (3 μM) used during screening. Despite the strong substrate inhibition, the improvement is not limited to *k*_cat_/*K*_M_ but extends to practical catalytic parameters: Both at low and high concentrations of the substrate, the total turnover numbers of the evolved variant are higher than that of the wild type (≈88-fold at low concentrations and still ≈20-fold at high concentrations, Figure S9).

The following scenario is consistent with the observation of substrate inhibition: the covalent intermediate without its bulky leaving group is much smaller than the substrate, potentially enabling a second substrate molecule to enter the active site and inhibit the reaction. The faster the first step (intermediate formation), the more pronounced this effect will be—as observed in the evolution from wild type to P91-R2.

### Rationalization of the Effects of the Identified Mutations

**Figure 7:**
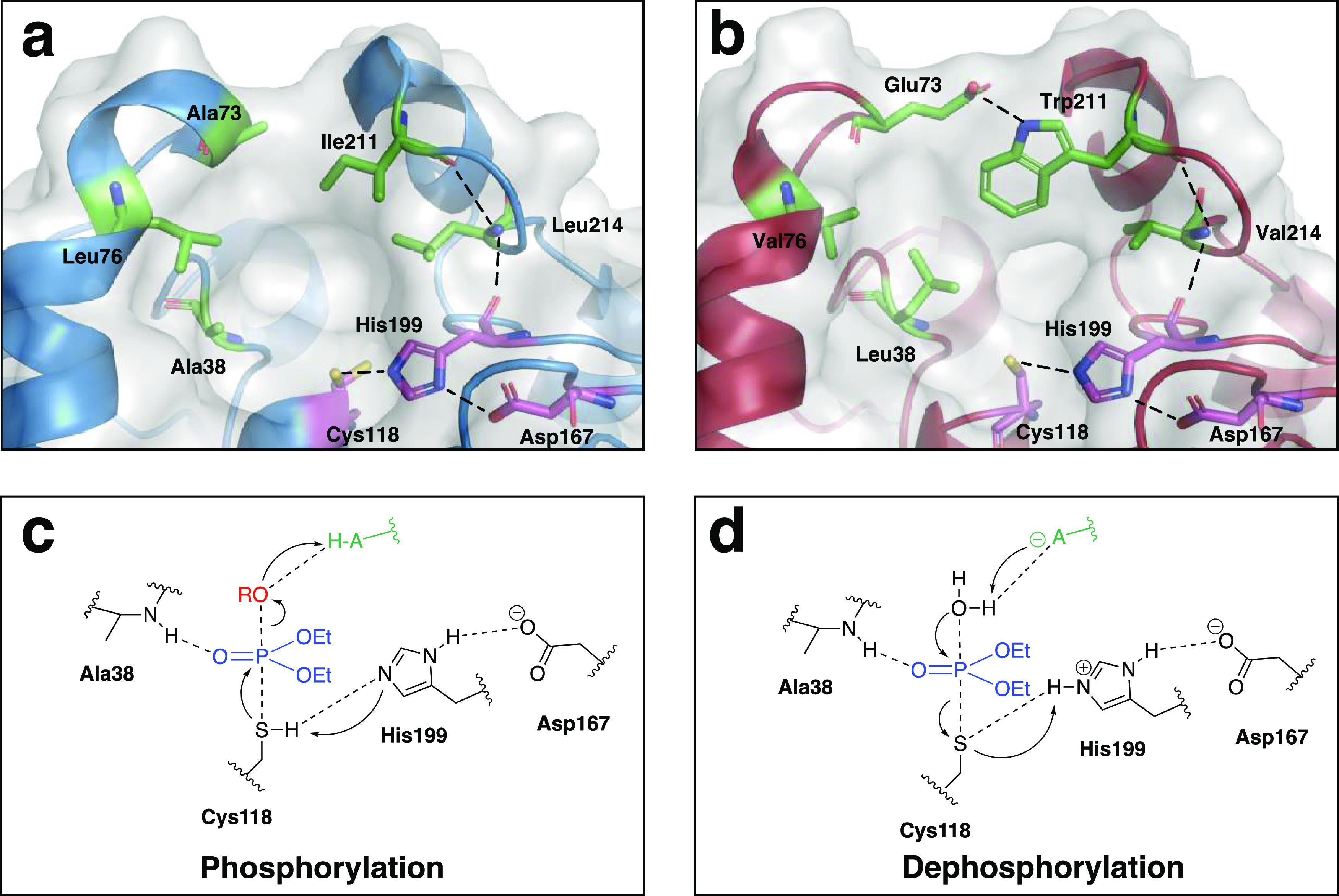
Rationalization of the effects of the identified mutations. (a) Positions identified in the preliminary mutational scanning shown in the structure of P91-WT (blue). The catalytic triad is shown in magenta, and positions chosen for mutation in the library are shown in green. Hydrogen bonds and putative new polar interactions are highlighted as dotted lines. (b) Mutations in P91-R2 (red) are shown in a structure created by AlphaFold2/ColabFold.^50,51^ P91 hydrolyses phosphotriesters via a mechanism involving the (c) formation (phosphorylation at rate constant *k*_2_) and (d) breakdown (dephosphorylation with rate constant *k*_3_) of a covalent intermediate. Both transition states have a trigonal–bipyramidal geometry around the phosphorus center during the formation of the covalent adduct and the breakdown of the covalent intermediate. His199 and Asp167 form a charge relay system, with His199 acting as a general acid/base catalyst. Ala38 contributes with its backbone amide to stabilization of the oxyanion and forms an oxyanion hole. The phosphate moiety is shown in blue, and the leaving group is shown in red. Leaving group charge offset and deprotonation of the water molecule are achieved with the participation of as yet unidentified residues of the enzyme, depicted as “HA/A^–^” in green.

The residues with the highest individual and combined effects upon mutation are Ile211 (Trp211 in the evolved variant) and Leu214 (Val214 in the evolved variant), both located in a loop that is partly covering the active site entrance (Figure 7a). As suggested by an AlphaFold2/ColabFold structural model of P91-R2,^50,51^ the mutated residues in this active site loop are interconnected with the catalytic triad through polar contacts along the backbones of Leu214 and Ile211’s down to His199 (Figure 7b). The likewise mutated residue Glu73 is possibly integrated into these extended interconnections through potential new polar interactions with Trp211 (the exact nature of this interaction is, however, obscured by the limited confidence of the structural model regarding the exact positioning of their side chains).

This new chain of interactions may subtly tweak the positioning of the involved residues to better accommodate the geometry of the phosphate transfer, consistent with the observed acceleration of this step (increase of *k*_2_) through specificity changes and pre-steady-state kinetics (Figures 4 and 5). Specifically, subtle re-positioning of His199 could facilitate leaving group charge offset at the apical position. In addition, the positioning of the new large aromatic residue Trp211 might allow π–π-interactions with the aromatic leaving group fluorescein. Both would increase leaving group stabilization, consistent with the observed flattening of the Brønsted slopes (increase of β_LG_, Figure 6).

In analogy to the canonical mechanism for ester hydrolysis in the same fold, the backbone nitrogen amide NH of Ala38 may stabilize the oxyanion formed during the transition state. The mutation to Leu38 in P91-R2 might thus additionally subtly contribute to the optimization of the transition-state geometry of the phosphorylation step (Figure 7c), while also potentially catalytically contributing to the dephosphorylation step (Figure 7d).

In conclusion, the reshaping of the active site by the mutations identified in the evolved variant P91-R2 might accelerate the rate-limiting phosphorylation step by removing a charge incompatibility in its transition state and thus optimizing for its, compared to the native esterase activity, new geometry. It should be noted that this arrangement is not an exclusive solution to higher affinity: other mutants with high activity—albeit not reaching the efficiency of P91-R2—exist (see Figure S20 and Table S7).

### Comparison with Other Serine Triad Enzymes

Prior to this work, metal catalysis was considered the only efficient mechanistic solution for phosphate triester hydrolysis identified in nature, having convergently evolved in different protein folds on a very short evolutionary time scale. While almost all serine-containing catalytic triads (as in acetylcholinesterase) are irreversibly inhibited by organophosphates, there is also evidence that adaptive evolution can activate these enzymes for multiple turnovers. The only known naturally evolved α/β hydrolase that can escape organophosphate inhibition at significant rates is a single mutant of an insect esterase with a Ser-His-Glu catalytic triad that evolved in response to insecticide exposure.^52,53^ Furthermore, a bacterial esterase,^54^ human butyrylcholineserase,^55,56^ and a snake acetylcholinesterase^57^ have evolved (or have been engineered) to slowly reactivate from organophosphate inhibition by hydrolysis of the covalent intermediate. In each case, the dephosphorylation rate constant (and thus the overall turnover rate constant) of all of those serine triad hydrolases is very slow (≈10^–4^–10^–2^ s^–1^) and in good agreement with the rate constants observed for the nucleophile-exchanged P91 variants (which bear a serine instead of the cysteine, Table S3). In contrast, the evolved P91-R2 variant displays a turnover rate constant that is at least two to four orders of magnitude higher than that of the serine enzymes (≈10^1^–10^2^ s^–1^, Table 1). Conversely, the rate constant of intermediate formation *k*_2_ is reported to be approximately 2 orders of magnitude faster in the insect esterase mutant, a serine enzyme, as compared to P91-WT, a cysteine enzyme (for further details, see the comparison with serine triad enzymes in Note S1.17 in the Supporting Information).^58^ This observation supports the conclusion that the identity of the nucleophile (serine or cysteine) determines the rate-limiting step. In summary, the limited catalysis by these serine enzymes stands in stark contrast to the ready evolvability of the cysteine enzyme P91 and the activity of the improved mutant P91-R2 observed in this work.

This finding also raises the question of whether known targets of organophosphates, such as human acetylcholinesterase and its homologues, would become proficient phosphotriesterases when exchanging their catalytic serine for a cysteine. Nucleophile exchange in a triad is usually deleterious to the native activity but has been shown to be rescuable by directed evolution.^59,60^ Given that such nucleophile exchanges have a low probability in natural nontargeted randomization events and that the known catalytically phosphotriesterase-activating mutations (reviewed above) have modest effects, it can be explained that metal-dependent hydrolases won out in the evolutionary experiment played out over the last decades in response to phosphotriester contamination.

### Comparison with Metal-Dependent Phosphotriesterases

When compared to metal-dependent organophosphate-degrading enzymes that have either naturally evolved (isolated from organophosphate-contaminated environments) or were engineered in the laboratory (by directed evolution or rational choice) from promiscuous enzymes, the rate constants of P91-R2 matches most of them and fall within 2 orders of magnitude of the best. The fastest known metal-dependent phosphotriesterase was isolated from *Brevundimonas diminuta* (*Bd*PTE), which achieves catalytic efficiencies approaching the diffusion limit (Figure 3b, bar l).^61^*Bd*PTE has been the target of numerous directed evolution campaigns, mainly toward chemical warfare agents, but has also been further evolved toward paraoxon hydrolysis, reaching an even higher *k*_cat_, however, with low further increases in overall *k*_cat_/*K*_M_.^62^ It should be noted that the catalytic efficiency of *Bd*PTE is exceptional as most naturally evolved or artificially engineered metal-containing phosphotriesterases have efficiencies in the range of 10^4^–10^6^ M^–1^ s^–1^ (Table S2).^23^ The *k*_cat_/*K*_M_ of P91-R2 is similar to (or even surpasses some of) the catalytic efficiencies of these metal-dependent phosphotriesterases (including their evolved mutants; Figure 3b).

P91 and “conventional” metal-dependent enzymes differ in the extent of substrate inhibition, making use of P91 for bioremediation or detoxification only practical at low substrate concentrations (≪*K*_M_). For higher substrate concentrations, the comparisons are less favorable for P91 because the substrate-inhibited enzyme predominates. This could be an intrinsic limitation but may also just be a reflection of the conditions under which P91 has evolved (i.e., low substrate concentration of 3 μM). In this concentration regime, there is a clear effect of evolution (in Michaelis–Menten parameters but also in the observed turnovers, Figures S7 and S9a). However, currently, metal-containing phosphotriesterases are superior in applications where maximal total turnover under substrate-saturating conditions is required, while P91 retains its practical utility at low substrate concentrations of below 50 μM. This could impede P91’s large-scale direct practical use, e.g., for bioremediation.

In addition, the improvements in catalytic efficiency are restricted to the fluorogenic substrate FDDEP used in the droplet screening. While P91-R2 shows a 360-fold improvement for FDDEP, its improvement for paraoxon is only 3.6-fold (Figure 5). Such (over-)specialization for a model substrate and high selectivity for a specific leaving group are well-known limitations of directed evolution campaigns using high throughput assays with proxy substrates, as summarized in the adage “you get what you screen for.”^63^ Nevertheless, mutants emerging from the screen for FDDEP are also improved in the promiscuous activity for the phosphotriester paraoxon (with a similarly activated leaving group with a p*K*_a_ of 7.14, compared to 6.7 for fluorescein):^64^ While P91-R2 improved only ≈3.6-fold, the variant P91-GGRG found in round 2 has a ≈25-fold improved activity for paraoxon (Figure S20c and Table S7). This suggests that the primary screening with a fluorescein group contains a subset of mutants that turn over smaller but also hydrophobic leaving groups efficiently. This coincidental specificity could be used as a starting point for directed evolution by plate screening for paraoxon turnover. We note that even with the *p*-nitrophenol leaving group, the specificity for the phosphotriesterase activity (over carboxyesterease activity) has increased by more than one order of magnitude for P91-R2 (Figure 5, 3.6/0.13 ≈ 27-fold).

Directed evolution of metalloenzymes with promiscuous activities has been successful: a promiscuous member of the amidohydrolase superfamily (with lactones as the best hydrolytic substrate) has been improved in 10 rounds toward higher organophosphate hydrolase activity, reaching an efficiency of 2 × 10^4^ M^–1^ s^–1^ for paraoxon-ethyl and 1.1 × 10^6^ M^–1^ s^–1^ for a methyl phosphonate (Figure 3b,bars j,k and Table S2).^19^ Promiscuous phosphotriesterases from the β-propeller and the metallo-β-lactamase superfamilies have also been subjected to extensive laboratory evolution, reaching catalytic efficiencies in the range of 10^4^–10^6^ M^–1^ s^–1^ within 6 rounds (Figure 3b,bars e,f,i and Table S2).^18,20^ For diethyl-substituted phosphotriester substrates, improvements between 690-fold for an amidohydrolase,^19^ 1100-fold for a metallo-β-lactamase,^20^ and 130-fold for a β-propeller^18^ were achieved in 10, 6, or 6 rounds, respectively (Table S2, entries k, f, i). Thus, more rounds of evolution were necessary in these previous campaigns to achieve similar improvements compared to only two in this work. Taking the possible differences between the scaffolds and assay conditions aside, the vastly higher throughput of microfluidic droplet screening (as compared to conventional microtiter plate screening) led to similar absolute activities more quickly.

### Outlook

In this work, microfluidic droplet screening identified a P91 variant able to hydrolyze phosphotriesters at catalytic efficiencies matching those of many metal-dependent phosphotriesterases. The success of this adaptation by directed evolution suggests that a Cys-containing catalytic triad is an evolvable motif for this new function, in principle set-up for efficient hydrolysis of organophosphates, even though such adaptation has never been observed in nature.

Substrate inhibition above the substrate concentration used for screening (3 μM) means that for practical purposes, P91 has to be further optimized. Adapting P91 for bioremediation of highly toxic organophosphates with leaving groups that do not resemble fluorescein or nitrophenolate would require screening assays that precisely mirror the conditions of their respective application scenario, even though they implicate a drastically lower screening throughput. This might be achieved using neutral drift libraries, which allow the creation of small yet highly diverse libraries of high evolutionary potential.^43,66^ For an enzyme of practical use, e.g., as a bioremediator or as a therapeutic enzyme, further properties beyond catalytic activity would have to be optimized: broad specificity for different organophosphates of practical relevance, activity across a broad substrate concentration range, stability, and, in case of a therapeutic application, immunogenicity and pharmacokinetic and pharmacodynamic properties. In this context, it might be of interest for application as a catalytic bioscavenger against organophosphate poisoning that P91 has a human homologue, carboxymethylenebutenolidase.^67^ Furthermore, substrate concentrations relevant for this application will presumably be well below P91’s current *K*_i_, so that the observed substrate inhibition might be irrelevant. It may be possible in the future to overcome the substrate inhibition when screening is carried out at higher substrate concentrations (i.e., >200 μM, where substrate inhibition is strong). Practically, one would deal with the shorter reaction times at higher substrate concentrations by reducing the enzyme concentration in the droplets, e.g., using a weaker promoter or less inducer, to ensure stringent selection conditions in the same microfluidic design (integrated chip device, as shown in Figure S5).

The identification of P91 with its unprecedented catalytic motif in metagenomic libraries underlines the untapped catalytic versatility of ecological consortia.^23^ Ultrahigh-throughput methods provide the capacity to explore rare catalytic solutions that are infrequent among extant enzymes. These additional reactivities may form second lines of evolutionary contingency, even against anthropogenic chemical compounds that environments have never “seen.” Here, we show that this contingency is not limited to a transient, low-efficiency catalyst but that initial catalytic solutions are further “evolvable” and exploitable, at least when large, smart libraries in microfluidic droplets with high throughput are used. The evolutionary improvements demonstrated here make them—in *k*_cat_/*K*_M_—similarly efficient as the previously known metal-containing phosphotriesterases. This suggests that not only accidental low-efficiency promiscuous catalysts but also alternative enzymatic contingencies exist in a given metagenome, ready to be recruited for a survival advantage (in natural evolution) and with the potential to become truly proficient. A potential reason why nature did not recruit cysteine enzymes to evolve phosphotriesterases might lie in the high threshold for the initial promiscuous activity that is required for survival. When initial activities are low, and slow and steady evolution will not meet the survival challenge, the level of the promiscuous starting activity matters primarily. Natural repertoires thus hold a variety of solutions for catalytic challenges: even if not explored thus far, they are available to contribute new-to-nature reactions^68^ or mechanisms. For P91, we have been able to play catch-up and generate an enzyme that is broadly as proficient as existing natural (albeit recent) biocatalysts. That this was possible from a sequence previously not identified as a catalyst for this target reaction suggests that many “evolvable” starting points are there to be discovered: droplet microfluidics allows enzyme discovery in a manageable time frame, so that we do not have to rely exclusively on extant, functionally characterized enzymes to enlarge our repertoire of catalysts.

## Methods

### Cloning and Library Construction

Libraries of the *p*91 gene were constructed on the high-copy number (≈800 copies/cell), anhydrotetracyclin-inducible plasmid pASK-IBA5plus (IBA Life Sciences, Germany) bearing an N-terminal StrepII-tag. Single-site saturation libraries were constructed by Golden Gate Assembly^69^ with partly degenerate primers according to the “22-codon trick.”^33^ Multiple-site saturation libraries were constructed by the assembly of gene fragments into the full-length gene by assembly polymerase chain reaction (PCR) (library P91-A) or Golden Gate Assembly (library P91-B). The fragments were created by PCR with primers bearing the degenerate codons NNK (library P91-A) or NDT/VHG/TGG (library P91-B). Further details on cloning and library construction can be found in Note S1.2 in the Supporting Information.

### Library Screening in Microfluidic Droplets

Monodisperse water-in-oil microdroplets were generated with a microfluidic flow-focusing device (Figure S3a). Fluorocarbon oil (Novec HFE-7500, 3M) containing 0.5% (w/w) surfactant (008-FluoroSurfactant; RAN Biotechnologies) served as the oil phase. The two aqueous streams were supplied with the cell solution and with a 3 μM substrate solution containing lysis agents (0.7× BugBuster protein extraction reagent, Merck Millipore; 60 kU/mL rLysozyme, Novagen) in droplet assay buffer, respectively. For long incubation times in evolution round 1, requiring off-chip incubation, droplets were collected into a long polyethylene tubing (0.38 mm ID, 1.09 mm OD; Portex Smiths Medical), which was closed with a syringe needle after collection. For short incubation times in evolution round 2, requiring on-chip incubation, an integrated chip was used, combining a flow-focusing module, a delay line, and a sorting module on a single device (Figure S5). On the sorting chip (round 1) or the sorting module of the integrated chip (round 2), droplets were sorted according to their green fluorescence (excitation wavelength: 488 nm) at a rate of ≈300–1000 Hz. Plasmids from sorted droplets were recovered by de-emulsification with 1*H*,1*H*,2*H*,2*H*-perfluorooctanol (Alfa Aesar) and subsequent column purification and electroporation into highly electrocompetent *E. coli* cells (E. cloni 10G Elite, Lucigen). Further details on the microfluidic screening can be found in Notes S1.3–S1.10 in the Supporting Information.

### Kinetic Measurements and Data Analysis

#### Steady-State Kinetics

For steady-state kinetic measurements, His_6_-tagged P91 variants (expressed and purified as detailed in Note S1.12 in the Supporting Information) were used. The progress of the reaction was monitored by absorbance or fluorescence in a spectrophotometric microplate reader (Tecan Infinite 200PRO, Tecan, Switzerland) at 25 °C. To determine the Michaelis–Menten parameters *k*_cat_, *K*_M_, and (in case of substrate inhibition) *K*_i_, the initial rates were fitted to the Michaelis–Menten equation
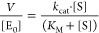
1or, in the case of substrate inhibition
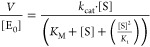
2where *v* is the initial rate of the reaction, [E_0_] is the initial enzyme concentration, and [S] is the substrate concentration.

#### Pre-Steady-State Kinetics

Fast transient-state kinetics for the hydrolysis of phosphotriesters FDDEP and paraoxon-ethyl were measured with a SX20 stopped-flow spectrophotometer (Applied Photophysics, U.K.) at the same temperature, in the same buffer, and with the same substrate concentrations as the steady-state kinetics. Measurement traces were fitted to the following exponential burst equation
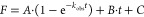
3where *F* is the measured absorbance or fluorescence, *t* is the time, *A* is the amplitude of the burst, *B* is the slope of the second phase of the reaction, and *C* is the offset.

The observed rate *k*_obs_ showed saturation behavior and was then fitted to the following equation to determine *k*_2_ (Figure S12 and Table S3).
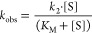
4The dephosphorylation rate constant *k*_3_ was separately determined with the mono-substituted substrate paraoxon-ethyl to exclude potential obfuscating effects due to the complex downstream kinetics of the double-substituted substrate FDDEP (where the original double-substituted substrate FDDEP and the initial reaction product, the mono-substituted FMDEP, compete for turnover). As the reaction with paraoxon-ethyl forms the same diethylphosphate covalent intermediate, *k*_3_ with FDDEP is identical to *k*_3_ with paraoxon-ethyl. Assuming a reaction model with one reversible binding step and two irreversible steps (Figure 4a), the turnover number *k*_cat_ and the catalytic efficiency *k*_cat_/*K*_M_ can be described as
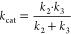
5
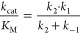
6Given that in this case *k*_2_ ≫ *k*_3_, the term for *k*_cat_ can be simplified to
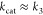
7Thus, the initial slope of the second phase of the burst reaction with paraoxon-ethyl was plotted against substrate concentration and fitted to the Michaelis–Menten equation to determine *k*_3_ (Figure S14 and Table S3). In cases where the burst is less pronounced (as in P91-R1 Cys118Ser), the *k*_cat_ is a lower estimate of *k*_3_, while the real value of *k*_3_ might be higher. Further details on kinetic measurements can be found in Note S1.13 in the Supporting Information.

## Supporting Information Available

Additional experimental procedures (chip design, microfluidic device operation, droplet sorting, DNA recovery, microtiter plate screening, protein purification, substrate synthesis, NMR data, gene sequences), figures (substrate structures, mutational scanning, kinetic data, NMR spectra), and tables (kinetic parameters, comparison of phosphotriesterase literature values, primer sequences) (PDF)
Microfluidic chip designs (ZIP)
