# Supplementary material for: Ultrahigh-Throughput Directed Evolution of a Metal-Free α/β-Hydrolase with a Cys-His-Asp Triad into an Efficient Phosphotriesterase
Source: J Am Chem Soc. 2022 Dec 30;145(2):1083–96. doi: 10.1021/jacs.2c10673 (PMC9853848; doi:10.1021/jacs.2c10673)
Supplement: Supplementary file 1 — ja2c10673_si_001.pdf [file ja2c10673_si_001.pdf]

Ultrahigh-throughput directed evolution of a metal-free α/β-hydrolase with a Cys-His-Asp triad into an efficient phosphotriesterase

Supplementary Information

J. David Schnettler, Oskar James Klein, Tomasz S. Kaminski, Pierre-Yves Colin, Florian Hollfelder*

Department of Biochemistry, University of Cambridge, 80 Tennis Court Road, Cambridge, CB2 1GA, United Kingdom

Correspondence: fh111@cam.ac.uk

Table of Contents

Supplementary Information ... 1

1. Supplementary Methods ... 4

1.1 Materials ... 4

1.2 Cloning and library construction ... 4

1.3 Chip design and preparation of microfluidic devices ... 5

1.4 Photolithographic fabrication of wafer master molds for microfluidic devices ... 5

1.5 Soft lithography protocol for preparation of PDMS chips ... 6

1.6 Preparation of cells for compartmentalisation ... 7

1.7 Compartmentalisation of cells into microdroplets ... 7

1.8 Fluorescence-assisted droplet sorting (FADS) ... 8

1.9 DNA recovery from microdroplets ... 8

1.10 Note on the enrichment achieved by droplet sorting ... 9

1.11 Microtiter plate screening ... 9

1.12 Protein expression and purification ... 9

1.13 Kinetic measurements ... 10

1.14 Note on rate comparisons for k_{cat}/K_M ... 11

1.15 Note regarding the determination of k_2 with the substrate FDDEP ... 11

1.16 Note on the presence of a covalent intermediate and on the ‘ageing’ side reaction ... 12

1.17 Details on kinetic comparisons of P91 with homologous serine triad enzymes ... 13

1.18 Note on alternative conformations of the active-site cysteine ... 14

1.19 Structural modelling with AlphaFold2 ... 14

1.20 Synthesis of phosphotriesters for linear free energy relationship measurements ... 14

2. Supplementary Figures ... 16

Figure S1: Structures of the substrates used in this study. ... 16

Figure S2: Structure-guided mutational active-site scanning. ... 17

Figure S3: Design of microfluidic chips for droplet generation and sorting (off-line droplet incubation). ... 18

Figure S4: Lysate activity distribution of library P91-A (round 1) before and after successive droplet sorting. ... 19

Figure S5: Design of microfluidic chips for on-chip droplet incubation. ... 20

Figure S6: On-chip fluorescence measurements for the adjustment of reaction time and sorting stringency. ... 21

Figure S7: Close-up view of the low concentration range of the Michaelis-Menten plot (Figure 3) of wild-type and evolved P91 with FDDEP. ... 22

Figure S8: Michaelis-Menten plots of steady-state kinetics ... 23

Figure S9: Total turnover numbers 24
Figure S10: Wild-type P91 (containing a cysteine triad) does not show biphasic burst kinetics..24
Figure S11: Stopped-flow reaction traces 25
Figure S12: Determination of the phosphorylation rate k_2. 26
Figure S13: Burst traces of all characterized P91 Cys118Ser variants 27
Figure S14: Determination of the de-phosphorylation rate k_3....................................28
Figure S15: Michaelis-Menten plots for steady-state kinetics of (a) P91-WT and (b) P91-R2 with linear-free energy relationship substrates 29
Figure S16: Example progress curve of FDDEP hydrolysis by the evolved variant P91-R2 30
Figure S17: Inhibition of the P91-R2-catalysed hydrolysis of FDDEP by fluorescein. 30
Figure S18: Quality control of the Brønsted analysis for substrate-specific binding effects. 31
Figure S19: Iterative saturation mutagenesis (ISM) of P91 at the three positions A73, I211 and L214. 32
Figure S20: Michaelis-Menten plots for steady-state kinetics of alternative screening hits 32
3. Kinetic data and comparisons 33
Table S1: Steady-state catalytic parameters 33
Table S2: P92-R2 rivals the efficiencies of engineered and naturally evolved metal-dependent phosphotriesterases. 34
Table S3: Microscopic rate constants 36
Table S4: Properties of phosphotriester substrates (paraoxon-ethyl derivatives 36
Table S5: Steady-state catalytic parameters for linear free-energy relationship of P91-WT 36
Table S6: Steady-state catalytic parameters for linear free-energy relationship of P91-R2 36
Table S7: Steady-state catalytic parameters of other P91 variants identified in round 2 for phosphotriester hydrolysis,...36
4. Sequences 38
4.1 Sequences of P91 variants and plasmid constructs 38
4.2 Primer sequences 45
5. NMR spectra...48
Supplementary References:...54

1. Supplementary Methods

1.1 Materials

All chemicals were purchased from Sigma-Aldrich and all biological reagents from New England Biolabs, unless otherwise stated. The fluorogenic model substrate fluorescein di(diethylphosphate) (FDDEP, 1) was synthesized as previously described.[1] The phosphotriesters **5–10** for linear free energy relationship measurements were synthesized as detailed below (**section 1.20**).

1.2 Cloning and library construction

Single-site saturation libraries were constructed using the 22-codon trick:[2] primers bearing the degenerate codons NDT, VHG and TGG were mixed in the ratio 12:9:1, in order to achieve balanced amino acid representation while avoiding stop codons, allowing to sufficiently oversample the diversity of a single randomized position with a single 96-well microtiter plate. The gene was amplified from the randomized position in a whole-plasmid PCR with Q5 DNA Polymerase and digested and re-circularized in a single step of Golden Gate Assembly using BsaI-HFv2 and T4 DNA ligase.[3] Single-site mutagenesis was carried out according to the same principle.

Multiple-site saturation library (P91-A, round 1): To construct multiple site-saturation libraries, the P91 gene was sub-divided into suitable fragments such that each randomized position was covered by a primer with the degenerate codon NNK. The fragments were created individually by PCR and then assembled by a further assembly PCR. Forward primers for fragment creation contained degenerate codons and ≈ 20 base pair homology arms to the neighboring fragments with an annealing temperature of 55 °C. For assembly PCR, the individual fragment PCR products were pooled and amplified with outer primers by PCR with Phusion DNA Polymerase at the annealing temperature of the homology arms. The resulting PCR product was column-purified (DNA Clean & Concentrator-5 Kit; Zymo Research) and digested with NheI and HindIII. After gel purification, the library insert was ligated into accordingly digested pASK-IBA5plus vector backbone with T4 DNA Ligase at 16 °C for 14 h. For plasmid amplification, the column-purified ligation product was electroporated into highly electrocompetent *E. coli* (E. cloni 10G Elite, Lucigen). After overnight incubation, the bacterial carpet was scratched off the agar plate with 6 ml of LB medium and the DNA was extracted with a plasmid isolation kit (GeneJET Plasmid Miniprep Kit; Thermo Fisher). The quality of the library was assessed by measurement of the peak heights in the sequencing chromatogram of a pooled sample as previously described.[4]

Multiple-site saturation library (P91-B, round 2): For the second round, the library was created by whole-plasmid PCR with degenerate primers containing a type-II restriction site overhang (BsaI: GGTCTCN) to create sticky ends for ligation. At the desired position, forward primers contained the mutagenic trinucleotides NDT, VHG and TGG and were used as a 12:9:1 mixture, previously described as the '22-codon trick'.[2] After whole-plasmid PCR with Phusion DNA Polymerase (ThermoFisher) or Q5 DNA Polymerase the PCR mix was digested with DpnI to selectively remove wild-type template. Subsequently, the amplicons were column-purified (DNA Clean & Concentrator-5 Kit; Zymo Research) and digested with BsaI to create

sticky ends. After a further column purification, amplicons were self-ligated with T4 DNA Ligase. The ligation product was amplified and purified in the same way as the round 1 library.

1.3 Chip design and preparation of microfluidic devices

The channel layout for the microfluidic chips was designed using AutoCAD (Autodesk, USA) and printed out on a high resolution film photomask (Micro Lithography Services, UK). The designs are shown in **Figures S3 and S4** and are deposited as DXF files on DropBase (https://openwetware.org/wiki/DropBase:Devices):

https://openwetware.org/wiki/Dropbase:_3-pL-droplets-01
https://openwetware.org/wiki/DropBase:droplet_electrosorting_3
https://openwetware.org/wiki/DropBase:droplet_electrosorting_4
https://openwetware.org/wiki/DropBase:droplet_electrosorting_5

1.4 Photolithographic fabrication of wafer master molds for microfluidic devices

The microfluidic devices were fabricated following standard photolithography and soft lithography protocols.[5] In brief, a silicon wafer (Prime Grade, 3 inch diameter, Czochralski Silicon (CZ-Si) wafer, thickness = 381 ± 20 µm, one-side polished; purchased from Microchemicals, Germany) was covered with a thin layer of SU-8 2000 series photoresist material using a spin coater (SPIN150i spin coater, Polos by SPS, Germany). Depending on a channel height, SU-8 2010 or SU-8 2025 photoresists were used (Kayaku Advanced Materials, Japan). Next, the wafer was soft-baked on a hot plate and the channel pattern was subsequently patterned into the master by photolithography using a mask aligner (MJB4 mask aligner; Süss MicroTec, Germany). In subsequent steps, the wafer was post-baked and, in the case of two-layer devices, the next layer of SU-8 resist was spincoated, followed by a second round of soft-baking, exposure and post-baking. After the post-baking step, the single- or double-layer chip, was developed in propylene glycol monomethyl ether acetate (Sigma Aldrich). Next, the wafer was hard-baked for 10 min at 200 °C and the hights of the structures were measured using a profilometer (Veeco Dektak 6M Stylus Surface Profilometer; Bruker, USA). Finally, the chip was silanized by deposition of pure trichloro(1H,1H,2H,2H- perfluorooctyl)silane (2 µL) in close proximity to the wafer placed in a Petri dish which was kept for 30 min in the vacuum chamber at 20 mbar to generate vapours of silane. A detailed overview of the steps of the protocol for the different devices is shown in **Table S0**.

Table S0. Protocols of photolithography of master mold of microfluidic devices used in this study.

	**Device type**				
	Flow focusing droplet generation device	FADS device	FF devices with delay lines and FADS module		
			Layer A	Layer B1 (20 loops device)	Layer B2 (5 loops device)
Nominal thickness (in µm)	12	20	15	30	15
Photoresist used	SU8-2010	SU8-2025	SU8-2010	SU8-2025	SU8-2010
Spin coating speed	1st step: 10 s, 500 rpm<br>2nd step: 30 s, 2000 rpm	1st step: 10 s, 500 rpm<br>2nd step: 30 s, 4000 rpm	1st step: 10 s, 500 rpm<br>2nd step: 30 s, 1600 rpm	1st step: 10 s, 500 rpm<br>2nd step: 30 s, 3000 rpm	1st step: 10 s, 500 rpm<br>2nd step: 30 s, 3400 rpm
Soft baking	3 min at 95 ºC	1 min at 65 ºC<br>5 min at 95 ºC	3 min at 95 ºC	1 min at 65 ºC<br>5 min at 95 ºC	3 min at 95 ºC
Exposure (at ~10 mW cm^{-2})	2 x 6 s, 2 s waiting time	2 x 7 s, 2 s waiting time	2 x 7 sec, 2 s waiting time	2 x 7.5 s, 2 s waiting time	2 x 7 s, 2 s waiting time
Post baking	3 min at 95 ºC	1 min at 65 ºC<br>5 min at 95 ºC	3 min at 95 ºC	1 min at 65 ºC<br>5 min at 95 ºC	3 min at 95 ºC
Development in the beaker filled with 30-50ml of PGMEA (Propylene glycol methyl ether acetate, Sigma Aldrich)	Approx. 5 min until all uncured SU-8 is removed from the wafer.<br>The development time depends on the intensity of manual agitation (for 2-layer chips, implemented only after deposition of the layer B)				
Hard baking	10 min at 200 ºC<br>(for 2-layer chips a hard baking step was applied only after development of the final device)				
Measured range of thicknesses (in µm)	11.8–11.9	21.5–23	14.7–15.0	*Total thickness of layer A and B1:* 46–48	*Total thickness of layer A and B2:* 28–29

1.5 Soft lithography protocol for preparation of PDMS chips

For the fabrication of microfluidic PDMS chips, the master was covered with a mixture of poly(dimethyl)siloxane (PDMS) and curing agent (Sylgard 184 Silicone Elastomer Kit, Dow Chemical Company, USA) in a 10:1 ratio (w/w). After degassing and curing at 65 °C for approximately 3 h, the PDMS device was removed from the master and holes for tubing connections were punched using a 1 mm biopsy punch with a plunger (Kai Medical, Japan). The device was then attached to a 1 mm thick microscope glass slide (flow-focusing device) or a 0.13 mm thin glass cover slip (sorting and delay line devices) by treatment with oxygen plasma (Femto plasma system; Diener Electronic, Germany) for 30 s, followed by a baking step of ≈ 20 min incubation at 65 °C. For hydrophobic treatment of the channel surface, a

freshly prepared and filtered solution of 1 % (v/v) trichloro(1H,1H,2H,2H-perfluorooctyl)silane in fluorinated oil (Novec HFE-7500, 3M, USA) was injected into the channels, followed by approximately 45 min of incubation on a hot plate at 65 °C. For small devices, the silane-containing oil was slowly injected by manual operation of a syringe. In contrast, for devices with a delay line, manual injection could lead to damage on the chip (due to high resistance and build-up of back-pressure) and therefore a syringe pump was used at at rate of 200 µL/h to inject the silane-containing oil.

1.6 Preparation of cells for compartmentalisation

Escherichia coli cells (E. cloni 10G Elite; Lucigen, USA) were transformed with 2.5 µL of library plasmids, yielding 10^6–10^7 colonies after overnight incubation on agar plates, as determined by serial dilution. Transformed cells were induced with anhydrotetracycline (final concentration 200 ng/mL; IBA Life Sciences, Germany) and incubated for expression for 14 h in 20 mL LB medium at 20 °C and 220 rpm shaking. After expression, the cells were washed five times with buffer (100 mM MOPS-NaOH, 150 mM NaCl, pH 8.0) and diluted to OD_{600} = 1.0. A 200 µL aliquot of the suspension was diluted 1:2 with 100 µL droplet assay buffer (100 mM MOPS-NaOH, 150 mM NaCl, pH 8.0, cOmplete EDTA-free protease inhibitor (one tablet per 50 mL; Roche, Switzerland) and 100 µL Percoll (a silica nanoparticle solution to reduce cell-cell adhesion and prevent sedimentation of cells in the syringe; Cytiva, USA) to a bacterial density of OD_{600} = 0.5. This bacterial suspension was diluted in order to match the desired final average bacterial droplet occupancy (which determines the specific occupancy of individual droplets according to the Poisson distribution). For instance, in a droplet volume of ≈ 3 pL a final bacterial density of OD_{600} = 0.25 resulted in 16 % of droplets with a single bacterium and 3 % of droplets containing two or more bacteria, as determined by microscopic imaging. All solutions (except for cell suspensions) were previously filtered with 0.2 µm PTFE syringe filters (Acrodisc CR 13 mm syringe filters, PALL Life Sciences, USA) to avoid clogging of microfluidic channels.

1.7 Compartmentalisation of cells into microdroplets

Monodisperse water-in-oil microdroplets were generated with a microfluidic flow-focusing device (design and fabrication, of microfluidic devices are described in the Supplementary Information). The device was connected via PE tubing (0.38 mm inner diameter, 1.09 mm outer diameter; Portex Smiths Medical, USA) to glass syringes (100 µL and 1 mL; SGE Analytics, Australia), which were driven by syringe pumps (neMESYS, Cetoni, Germany). Fluorocarbon oil (Novec HFE-7500, 3M, USA) containing 0.5 % (w/w) surfactant (008-FluoroSurfactant; RAN Biotechnologies, USA) served as oil phase. The two aqueous streams were supplied with the cell suspension and with a 3 µM substrate solution containing lysis agents (0.7× BugBuster protein extraction reagent, Merck Millipore; 60 kU/mL rLysozyme, Novagen) in droplet assay buffer, respectively. The enzymatic reaction was initiated by cell lysis upon droplet formation from the three supply streams. Droplet formation was monitored using an inverted microscope (SP98I, Brunel Microscopes, UK) with a high-speed camera (Phantom Miro eX4, Vision Research, USA). For long incubation times in evolution round 1, requiring off-chip incubation, flow rates of 50 µL/h for the aqueous phases and 500 µL/h for the oil phase were used to generate droplets with a volume of 3 pL at rates of 0.5–3 kHz. Droplets were collected into a

long PE tubing (0.38 mm ID, 1.09 mm OD; Portex Smiths Medical, USA) which was closed with a syringe needle after collection. For short incubation times in evolution round 2, requiring on-chip incubation, an integrated chip was used, combining a flow-focussing module, a delay line, and a sorting module on a single device (**Figure S4**). For tight spacing of the droplets in the delay line, required for even mixing and homogenous incubation times, oil was removed through an oil extractor. At the end of the delay line, droplets were injected into the sorting module. On this chip, flow rates were 7.5 µL/h for the aqueous phases, 25 µL/h for the oil phase, ≈ -10 µL/h for the oil extractor, and ≈ 300 µL/h for the spacing oil, resulting in a droplet volume of ≈ 11 pL.

1.8 Fluorescence-assisted droplet sorting (FADS)

Optics and electronics of the microfluidic on-chip sorting device were set-up as previously described[6,7]. After incubation at room temperature, droplets were reinjected from the collection tubing into the sorting device at a rate of 10–25 µL/h. To enable precise sorting of single droplets, the distance between the droplets was increased by injection of spacing oil (Novec HFE-7500, 3M, USA) into the device at a flow rate of 100–300 µL/h. The asymmetric Y-shaped junction in the device ensures that all droplets automatically flow into the waste channel, unless deviated by an electrical pulse into the sorting channel. A 488-nm laser was focused 100 µm upstream of the sorting junction through a 40× microscope objective (UPlanFLN, Olympus, Japan) for fluorophore excitation and the emitted fluorescent light was collected and amplified using photomultiplier tubes (PMM02, Thorlabs, USA). Whenever the fluorescence peak reached a user-defined threshold, an electric field was applied by the two electrodes on the sorting device, attracting the highly fluorescent droplet towards the narrower sorting channel (**Figure 1**). Droplets were sorted into a collection tube pre-filled with 100 µL nuclease-free water.

1.9 DNA recovery from microdroplets

Plasmids from sorted droplets were recovered by de-emulsification with 1H,1H,2H,2H-perfluorooctanol (Alfa Aesar, USA) and subsequent column purification and electroporation into highly electrocompetent E. coli cells (E. cloni 10G Elite, Lucigen, USA) in a modification of the previously described protocol.[6] In brief, after droplet sorting, 400 ng of salmon sperm DNA (Invitrogen, USA) were added to the collection tube. Subsequently, PFO (approx. half the volume of the collected oil volume; e.g., 400 µL PFO for 800 µL oil in the collection tube) was added. The tube was vortexed for ≈ 60 s and centrifuged for 1 min at 14000 g. The top aqueous layer was completely removed and transferred into a new tube. The oil phase remaining in the collection tube was re-extracted by adding 100 µL nuclease-free water, 400 ng salmon sperm DNA, 100 µL PFO, vortexing for 1 min and subsequent centrifugation for 1 min at 14000 g. The top aqueous layer was completely removed and united with the previously removed aqueous layer. The united aqueous fractions usually contain a small remaining bottom oil phase. This remaining oil phase was then extracted by addition of 100 µL PFO, 1 min of vortexing, and 1 min of centrifugation at 1400 g. The top aqueous phase was then removed without any traces of oil phase and the plasmid DNA was column-purified using a DNA Clean & Concentrator Kit (Zymo Research, USA) according to the manufacturer's instructions, with the following modifications: The ratio of binding buffer to sample volume was 6:1. The wash

buffer was carefully rinsed along the walls. After the second wash step, the flow-through was removed and the column was centrifuged once more for 1 min to dry. For elution, 6 µL of pre-warmed (50 °C) elution buffer were used. The eluted DNA was then electroporated into highly electrocompetent E. coli cells (E. cloni 10G Elite, Lucigen, USA) according to the manufacturer's instructions. Throughout the DNA extraction procedure, low-DNA-binding tubes (1.5 mL DNA LoBind tube; Eppendorf, Germany) and low-retention tips (Axygen Maxymum Recovery Filter Tips; Corning, USA) were used.

1.10 Note on the enrichment achieved by droplet sorting

We carried out an enrichment experiment in which 1 % of droplets containing bacteria that express P91-WT were mixed with 99 % of droplets containing bacteria expressing the inactive variant P91 C118A. After encapsulation, sorting and DNA recovery, the recovered variants showed to consist of 31 % WT, corresponding to a ≈ 30-fold enrichment. This enrichment factor however will increase with the 'dilution rate' of the positive sample (e.g., a 0.01 % positive in 99.99 % negative mixing ratio will yield a much higher enrichment factor).[8] The enrichment factor will also depend on the activity difference of the compared variants and on the stringency of the sorting gate, making it impossible to assign a single enrichment factor to a whole library sorting.

1.11 Microtiter plate screening

To quantify the lysate activity of P91 variants, individual colonies were picked and grown in 96-deep-well plates in 500 µL Luria-Bertani (LB) medium with 100 µg/mL carbenicillin at 37 °C/1050 rpm for ≈ 14 h. Subsequently, 10 µL of overnight cultures were used to inoculate 490 µL of medium for expression cultures in 96-well deep-well plates which were grown at 37 °C/1050 rpm for ≈ 2 h until OD_{600} ≈ 0.5. Expression was then induced with anhydrotetracycline (final concentration 200 ng/mL; IBA Life Sciences, Germany) and carried out for 14 h at 20 °C and 1050 rpm shaking. Cells were pelleted by centrifugation at 3320 g for 60 min, the supernatant was then discarded, and cells were lysed with 100 µL lysis buffer (50 mM HEPES-NaOH, 150 mM NaCl, pH 8.0, 60 kU/mL rLysozyme, 1X BugBuster) for 20 min at 20 °C and 1050 rpm shaking. Cell lysates were diluted 1:20, 1:400, or 1:1600 in assay buffer (50 mM HEPES-NaOH, 150 mM NaCl, pH 8.0). For the reaction, 190 µL of the phosphotriester substrate paraoxon-ethyl (100 µM) or FDDEP in assay buffer (3 µM) were added to 10 µL aliquots of the diluted lysate in microtiter plates and the formation of fluorescein or *p*-nitrophenol was recorded in a plate reader (Infinite M200, Tecan, Switzerland) for 15 min at a wavelength of 405 nm for *p*-nitrophenol and at an excitation wavelength of 480 nm and an emission wavelength of 520 nm for fluorescein.

1.12 Protein expression and purification

Plasmids isolated from single colonies were used to transform BL21(DE3) cells. Expression cultures were then inoculated by a similar 'plating' method as previously described.[9,10] In brief, a dense lawn of freshly transformed BL21(DE3) cells was directly scraped into 500 mL TB medium containing 100 µg/mL carbenicillin. The cells were grown for ≈ 60 min at 37 °C/200 rpm before being induced with anhydrotetracyclin (final concentration 200 ng/mL). Protein was then expressed at 20 °C/200 rpm for 18–20 h. Cells were harvested by centrifugation at

4000 rcf for 10 min, the supernatant was discarded, and the dry pellet was stored at -80 °C. Note that the P91 construct for library screening originally contained an N-terminal StrepII-tag. For high-yield purification (as required for transient-state kinetics), the StrepII-tag was exchanged for an N-terminal 6xHis-tag. For purification, the pellet was resuspended in lysis buffer (50 mM HEPES-NaOH, 150 mM NaCl, pH 8.0, 1 mM TCEP, 20 mM imidazole, 0.5–1 mg/mL lysozyme, 0.1 % Triton X-100, 0.01 % (= 25 units/mL) benzonase nuclease) and rolled for 30–60 min at room temperature. Afterwards, the lysate was cleared by centrifugation at 20000 rcf/4 °C for 20 min and the soluble fraction was loaded onto a Ni-NTA gravity flow column (Super Ni-NTA Agarose Resin, Neo Biotech, France). The protein on the column was washed with 5 × 2.5 column volumes of wash buffer (50 mM HEPES-NaOH, 150 mM NaCl, pH 8.0, 1 mM TCEP, 20 mM imidazole) and eluted with 5 × 0.5 column volumes of elution buffer (50 mM HEPES-NaOH, 150 mM NaCl, pH 8.0, 1 mM TCEP, 250 mM imidazole). Subsequently, the eluate was concentrated with a spin concentrator (Vivaspin 10 000 kDa MWCO, Sartorius, Germany) and subsequently exchanged into imidazole-free assay buffer (50 mM HEPES-NaOH, 150 mM NaCl, pH 8.0, 1 mM TCEP) using PD 10 desalting columns (Cytiva, USA). The typical yield was ≈ 200 mg enzyme per liter of culture. Enzyme purity was controlled by SDS-PAGE and concentrations were determined by measurement of absorption at 280 nm on a NanoDrop 2000 Spectrophotometer (Thermo Fisher Scientific, USA), using an extinction coefficient calculated with the ProtParam web tool (https://web.expasy.org/protparam).[11]

1.13 Kinetic measurements

Steady-state kinetic measurements were carried out with His_6-tagged P91 variants and enzyme concentrations were kept at least 10–100-fold lower than the lowest substrate concentration. Substrate concentrations were chosen to span ≈ 10-fold below and above K_M, as far as not limited by substrate solubility. Optimal starting enzyme concentrations E_0 and substrate concentration ranges were determined for each variant and substrate combination by empirical sampling. Substrates were pre-dissolved in DMSO in stocks of 200-fold the final concentration in order to ensure constant DMSO concentration (0.5 %) across all substrate concentrations. The DMSO content was found to influence the catalytic parameters (while being required as a co-solvent for substrate stocks) and was therefore kept constant. Upon measurement, aliquots of these substrate stocks were diluted 1:100 in assay buffer (50 mM HEPES-NaOH, 150 mM NaCl, pH 8.0, 1 mM TCEP), of which 100 µL were subsequently mixed with 100 µL of 2-fold concentrated enzyme solution (= 2× E_0) in microtiter plate wells. The progress of the reaction was monitored by absorbance (for wavelengths and extinction coefficients for each substrate leaving group see Table S4) or fluorescence (at an excitation wavelength of 480 nm and an emission wavelength of 520 nm for fluorescein) in a spectrophotometric microplate reader (Tecan Infinite 200PRO, Tecan, Switzerland) at 25 °C. Absorbance measurements at wavelengths < 320 nm were recorded in a quartz 96-well plate. Absorbance maxima and extinction coefficients were determined for the leaving group of each substrate by absorbance wavelength scan followed by a calibration curve (see **Table S4**).The initial rates were extracted by linear fit of the first measurements (at < 10 % progress of the reaction) for each substrate concentration and normalized with an extinction coefficient determined from a calibration curve. Fitting of the data was done with R using the non-linear fitting function `nls()`.[12] In the

case of the kinetics of P91-WT and P91-R2 with FDDEP that were used for rate comparisons with other phosphotriesterases, three enzyme batches were measured and compared (as biological triplicates). The error indicated for these kinetic measurements thus represents the standard error of *three* replicates of independent enzyme preparations and purifications. The batch-to-batch variation in the measurements was shown to be small: the triplicates for FDDEP, where the three biological replicates of P91-WT yielded k_{cat}/K_M values of 1690, 1700, and 2000 M^{-1} s^{-1}. In the face of such small batch-to-batch variations that would not influence fits on a logarithmic Brønsted plot, no biological replicates were explicitly taken into account in the LFER analyses. Instead of a biological replicate, errors of the Michaelis-Menten parameters were derived from the non-linear curve fits and, in the Michaelis-Menten plots, estimated typical errors were plotted (9 %, derived from the mean measurement-to measurement error of the biological triplicate measurements of P91-WT and P91-R2 with FDDEP). When comparing kinetics of variants, samples were purified and assayed in parallel, with the same tags and buffers, to minimize possible variations due to purification method, affinity tag and buffer choice (that might influence kinetics, as seen in previous kinetic measurements of P91.[13]

1.14 Note on rate comparisons for k_{cat}/K_M

In order to test whether the curve fit for substrate inhibition distorts the k_{cat}/K_M we made additional comparisons: **Figure S7** shows the linear part of the Michaelis-Menten curves (v_0 vs. [S]) at sub-saturating concentrations (< K_M) for P91-R2 and P91-WT. This regime encompasses the substrate concentration where the screening was carried out ($[S]_0 = 3$ μM). When the slopes of the linear parts or the actual v_0 values at 3 μM are compared, accelerations of 380-fold and 450-fold, respectively, are obtained. This is similar to the 360-fold improvement in k_{cat}/K_M calculated by nonlinear fit across the entire substrate concentration. So, the extrapolations by curve fitting (**Figure 3**) and these more direct comparisons lead to identical conclusions about the acceleration that has been achieved, and the corresponding transition state stabilisation.

1.15 Note regarding the determination of k_2 with the substrate FDDEP

For the double-substituted phosphotriester FDDEP, the apparent k_2 as measured from the burst phase represents the hydrolysis of one of the two phosphotriester groups on the substrate and thus the release of the mono-phosphorylated reaction product, fluorescein mono(diethylphosphate), which has an unknown extinction coefficient. However, the observed burst amplitude (0.4–1.0, thus corresponding to the value expected for fluorescein release) indicates that most of the substrate fluorescence is generated by the release of this first group, thus allowing the use of a fluorescein calibration curve for the approximate quantification of k_2.

In brief, fast transient-state kinetics for the hydrolysis of phosphotriesters FDDEP were measured with a stopped-flow spectrophotometer. Measurement traces were fitted to the following exponential burst equation:

$$F = A \cdot (1 - e^{-k_{obs}t}) + B \cdot t + C \qquad \text{(equation 3)}$$

where F is the measured absorbance or fluorescence, t is the time, A is the amplitude of the burst, B is the slope of the second phase of the reaction and C is the offset.
The observed rate k_{obs} showed saturation behavior and was then fitted to the following equation to determine k_2 (**Figure S12, Table S3).**

$$k_{obs} = \frac{k_2 \cdot [S]}{(K_M + [S])} \quad \text{(equation 4)}$$

The substrate inhibition (P91-WT: $K_i \approx 290$ µM; P91-R2: $K_i \approx 7$ µM) may complicate the interpretation of this saturation behavior. We consider two minimal models:

(1) The second substrate binding occurs with the *un*phosphorylated enzyme.
In this case the second substrate binding event will be responsible for the saturation, so that the extrapolated k_2 is an underestimation of the true k_2 (and provides a lower limit for it). This means that the acceleration as a consequence of directed evolution is also *under*estimated.

(2) The second substrate binding occurs with the phosphorylated enzyme.
In this case the second substrate binding is not competing with the first binding event that then is solely responsible for the saturation.

If the first case were true, then substrate inhibition would be identical between cysteine and serine enzymes. This is, however, not the case (see **Figure 3a** and **Figure S12**). Conversely, assuming that serine and cysteine variants behave similarly in terms of Michaelis complex formation, the absence of substrate inhibition in the serine enzyme (in pre-steady state measurements in **Figure S12**; but also in the steady-state parts of the curves in **Figure S11**) suggests that the second model is more plausible. We therefore favor the second scenario, meaning that substrate inhibition does not interfere with the determination of k_2.

1.16 Note on the presence of a covalent intermediate and on the 'ageing' side reaction

In principle, the complete absence of an intermediate in the mechanism of P91 is also a possibility. However, the following arguments support the presence of a covalent intermediate:

1. In contrast to other known phosphotriesterases, which have a metal-assisted instead of a nucleophilic mechanism , P91 has no metal in the active site.[13]
2. Dienelactone hydrolase, the closest functionally characterized homolog of P91, shares the same active site triad (Cys-His-Asp) with P91 and forms a covalent intermediate via its cysteine nucleophile in the hydrolysis of esters and lactones.[14]
3. The phosphotriesterase and esterase activities of P91 compete for the same active site, suggesting that they use the same mechanism.[13]
4. Mutation of the active-site cysteine Cys118 to alanine abolishes both, esterase and phosphotriesterase activity in P91.[13]
5. Mutation of the active-site cysteine Cys118 to serine induces burst kinetics in P91 which are simplest explained by the presence of a covalent intermediate.

The difference in pK_a between serine and cysteine in different catalytic triads could potentially have consequences on the susceptibility for a side reaction, hydrolytic de-alkylation of the phosphotriester adduct, dubbed ageing: upon water/hydroxide attack at the phosphorylated intermediate, the transition state collapses with preferential loss of the best leaving group. In the case of a serine triad, one of the two ethoxy side groups of the phosphotriester is prone to leave instead of the serine, resulting in a negatively charged adduct which is resistant to further hydrolysis. As the cysteine thiol group is a much better leaving group than any of the phosphate's ethoxy side groups, P91 should be much more resistant to ageing than serine triad enzymes. This is reflected in the number of total turnovers per enzyme active site which is ≈ 12000 total turnovers for P91-R2 but drops to only ≈ 5 when its active-site cysteine is exchanged for serine (**Figure S9**).

1.17 Details on kinetic comparisons of P91 with homologous serine triad enzymes

The only known naturally evolved α/β hydrolase that can escape organophosphate inhibition at significant rates is an insect esterase with a Ser-His-Glu catalytic triad that is distantly related to P91 (sequence similarity to P91 ≈ 14 %, structural similarity: RMSD ≈ 4.3 Å). This Gly137Asp mutant of α-carboxylesterase αE7 (*Lc*αE7 Gly137Asp) has evolved very low phosphotriesterase activity in the blow fly *Lucilia cuprina* in response to insecticide exposure ($k_{cat}/K_M \approx 10^2$ $M^{-1}s^{-1}$).[15,16] However, this enzyme is mainly characterized by high affinity to pesticides and a very slow reactivation rate ($k_{cat} \approx 10^{-3}$ s^{-1}), its dephosphorylation rate being roughly four orders of magnitude lower than that of P91-R2.[17] It is thus fundamentally still a stoichiometric scavenger of pesticides rather than as fast-turnover enzyme. Even wild-type P91 is already 100-fold faster as a phosphotriesterase ($k_{cat} \approx 0.1$ s^{-1}).[13]
Similarly, human butyrylcholinesterase, a close homolog of synaptic acetylcholinesterase, has been engineered at the corresponding site (Gly117His) into a slow turnover enzyme, but also shows similar, slow turnover ($k_{cat} \approx 0.09$ s^{-1}).[18,19] A bacterial homolog, *p*-nitrobenzyl esterase from *Bacillus subtilis*, bearing the homologous mutation to BChE Gly117His (*Bs*pNBE Ala107His) showed very slow reactivation and could be further evolved to reach turnover rates of ≈ 1 h^{-1} (≈ 0.0003 s^{-1}) after treatment with paraoxon.[20] A triple mutant of a snake acetylcholinesterase (from *Bungarus fasciatus*) has been reported to display slow promiscuous organophosphate hydrolysis, albeit with very low catalytic efficiencies in the range of 10^{-1} to 10^1 $M^{-1}s^{-1}$, depending on the substrate.[21]
In each case the slow de-phosphorylation rate of all those serine triad hydrolases (≈ 10^{-4} to 10^{-2} s^{-1}) is in good agreement with the rates observed for the nucleophile-exchanged P91 variants (which bear a serine instead of the cysteine). The turnover rate of the evolved P91-R2 variant (≈ 10^1–10^2 s^{-1}) is two to four orders of magnitude higher than of the serine enzymes. Conversely, the rate of intermediate formation k_2 is reported to be 1.3 s^{-1} for the serine enzyme *Lc*αE7 mutant[17] and, inferring from k_{cat}, about two orders of magnitude lower in the cysteine enzyme P91-WT.

1.18 Note on alternative conformations of the active-site cysteine

Like in homologous dienelactone hydrolases, the catalytic cysteine of P91 assumes two conformations: an inwards-pointing protected and an outwards-pointing active conformation. For dienelactone hydrolases, it has been proposed that the inwards-pointing conformation is inactive and protects the catalytic cysteine from oxidation.[22] In P91, this alternative conformation is stabilized by hydrogen bonds to residues Glu37 and His144.[13]

1.19 Structural modelling with AlphaFold2

The structure of P91-R2 was modeled using AlphaFold2[23] via the ColabFold implementation[24] (https://colab.research.google.com/github/deepmind/alphafold/blob/main/notebooks/AlphaFold.ipynb).

1.20 Synthesis of phosphotriesters for linear free energy relationship measurements.

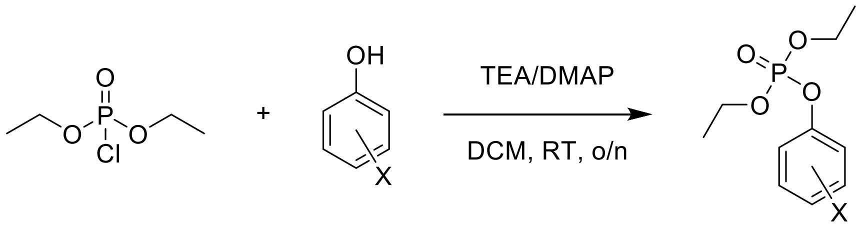

Synthesis procedures and pK_a values were adapted from Khersonsky & Tawfik 2005.[25] In brief, the respective phenol derivative (1 g) and diethyl phosphochloridate (1.2 equivalents) were dissolved in dichloromethane. Base (1 mol eq., triethylamine for all but 3-fluoro-4-nitrophenyl, where dimethylaminopyridine (DMAP) was used) was added dropwise and the reaction mixture stirred at room temperature (≈ 23 °C) overnight (≈ 16 h). Reaction progress was followed by thin layer chromatography (silica gel 60, EtOAc:Hex, 2:1). The reaction mixture was washed with HCl (100 mM, 50 mL), saturated Na_2HCO_3 (50 mL) and NaCl brine (50 mL, pH ≈ 7). Products contaminated by starting material were purified by flash chromatography (silica gel 60, EtOAc:Hex 2:1). Products were characterized by ^{1}H-NMR. NMR spectra can be found in section 5 of the Supplementary Information. All NMR data were collected at 298 K using Bruker Avance spectrometers with ^{1}H resonance frequencies of 400 MHz. Chemical shifts (δH) are reported in parts per million (ppm), to the nearest 0.01 ppm and are referenced to the residual non-deuterated solvent peak. Coupling constants (*J*) are reported in Hertz (Hz) to the nearest 0.1 Hz. Data are reported in the order: (i) chemical shift, (ii) multiplicity (s = singlet; d = doublet; t = triplet; q = quartet; m = multiplet; or as a combination of these, e.g., dd, dt etc.), (iii) coupling constant(s) and (iv) integration. Peak integrals were used to produce correction factors for residual solvent contamination.

3-fluoro-4-nitrophenyl diethyl phosphate (5)

^{1}H NMR (399.6 MHz, $CDCl_3$): δ (ppm) 8.14 (t, $^3J_{HH}$/$^4J_{HF}$ = 8.8 Hz, 1H), 7.24 (dd, $^3J_{HF}$ = 11.5, $^4J_{HH}$ = 2.4 Hz, 1H), 7.20 (m, 1H), 4.29 (m, 4H), 1.41 (td, $^3J_{HH}$ = 7.1 Hz, $^4J_{HP}$ 1.1 Hz, 6H).

4-formylphenyl diethyl phosphate (6)

^{1}H NMR (399.6 MHz, $CDCl_3$): δ (ppm) 10.00 (s, 1H), 7.92 (d, $^3J_{HH}$ = 8.5 Hz, 2H), 7.41 (d, $^3J_{HH}$ = 8.5 Hz, 2H), 4.27 (m, 4H), 1.39 (td, $^3J_{HH}$ = 7.1 Hz, $^4J_{HP}$ 1.0 Hz, 6H).

4-cyanophenyl diethyl phosphate (7)

^{1}H NMR (399.6 MHz, $CDCl_3$): δ (ppm) 7.68 (dd, $^3J_{HH}$ = 8.9, $^4J_{HP}$ = 0.5 Hz, 2H), 7.37 (dd, $^3J_{HH}$ = 8.9 Hz, $^4J_{HP}$ 0.9 Hz, 2H), 4.26 (m, 4H), 1.39 (td, $^3J_{HH}$ = 7.1 Hz, $^4J_{HP}$ 1.1 Hz, 6H).

4-acetylphenyl diethyl phosphate (8)

^{1}H NMR (399.6 MHz, $CDCl_3$): δ (ppm) 7.99 (d, $^3J_{HH}$ = 8.4 Hz, 2H), 7.34 (dd, $^3J_{HH}$ = 8.9, $^4J_{HP}$ 0.9 Hz, 2H), 4.26 (m, 4H), 2.61 (s, 3H), 1.39 (td, $^3J_{HH}$ = 7.1 Hz, $^4J_{HP}$ 1.1 Hz, 6H).

3-cyanophenyl diethyl phosphate (9)

^{1}H NMR (399.6 MHz, $CDCl_3$): δ (ppm) 7.56-7.45 (m, 4H), 4.26 (m, 4H), 1.40 (td, $^3J_{HH}$ = 7.1 Hz, $^4J_{HP}$ 1.1 Hz, 6H).

3-chlorophenyl diethyl phosphate (10)

^{1}H NMR (399.6 MHz, $CDCl_3$): δ (ppm) 7.30 (d, $^3J_{HH}$ = 8.2 Hz, 1H), 7.27 (m, 1H), 7.20-7.14 (m, 2H), 4.25 (m, 4H), 2.61 (s, 3H), 1.38 (td, $^3J_{HH}$ = 7.1 Hz, $^4J_{HP}$ 1.0 Hz 6H).

2. Supplementary Figures

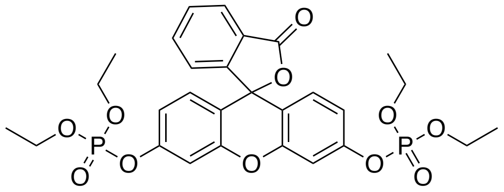

1

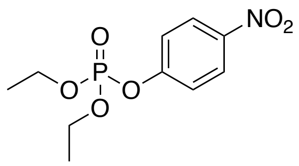

2

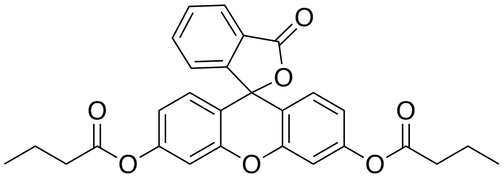

3

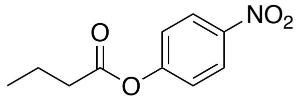

4

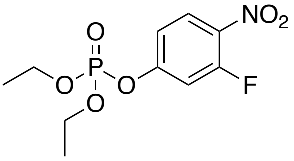

5

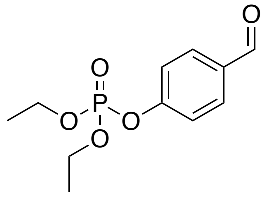

6

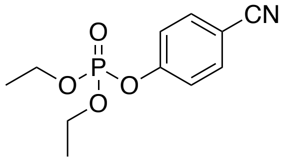

7

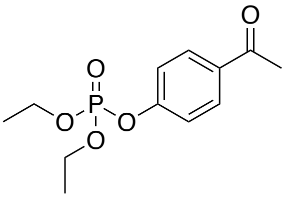

8

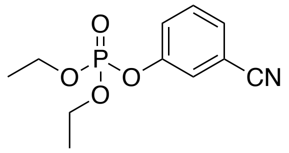

9

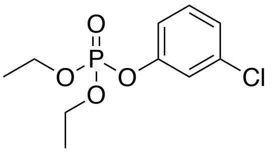

10

Figure S1: Structures of the substrates used in this study. 1: Fluorescein di(diethylphosphate) (FDDEP); **2:** Paraoxon-ethyl (PXN); **3:** Fluorescein dibutyrate; **4:** *p*-Nitrophenyl butyrate; **5**: 3-Fluoro-4-nitrophenyl diethylphosphate; **6**: 4-Formylphenyl diethylphosphate, **7**: 4-Cyanophenyl diethylphosphate, **8**: 4-Acetylphenyl diethylphosphate; **9**: 3-Cyanophenyl diethylphosphate; **10**: 3-Chlorophenyl diethylphosphate

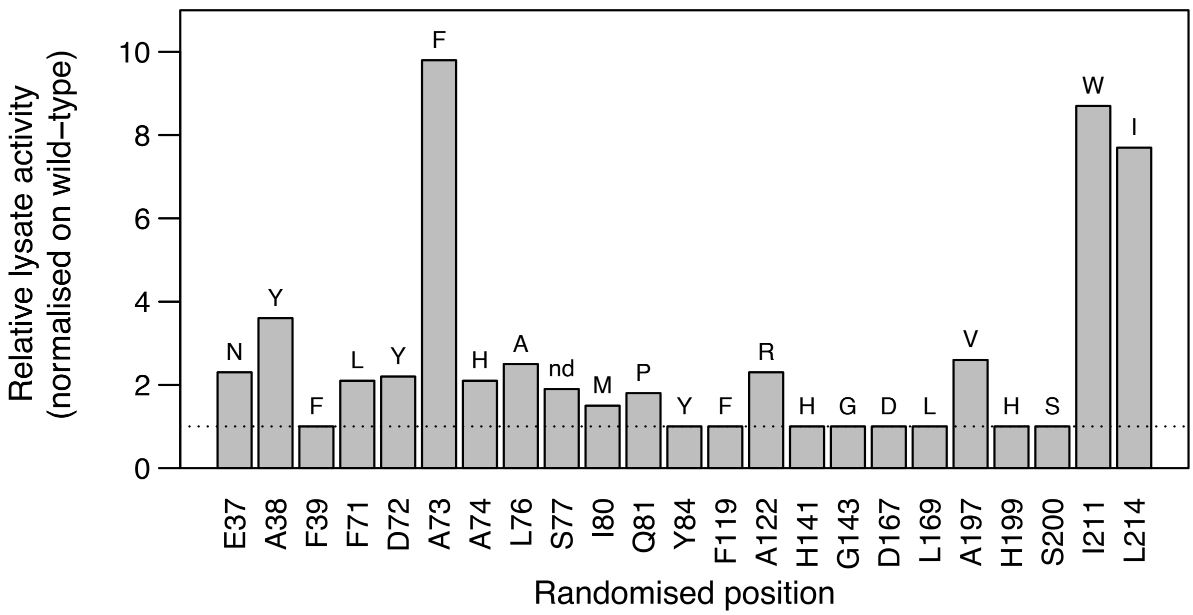

Figure S2: Structure-guided mutational active-site scanning. Positions lining the active site of P91 were individually randomized and screened for phosphotriesterase activity in microtiter plates using 1 μM FDDEP. Over four-fold oversampling of the theoretical diversity at each position ensures that with high probability every single amino acid substitution appears in the screen. The bars show the lysate activity (rate of product formation) of the respective best-performing clone at each position (indicated in single-letter amino acid code), relative to the wild type. The dotted line indicates wild-type level activity. The substituting amino acid of the respective most active clone is indicated on top of the bar; nd: not determined. Note that the triad residues Asp167 and His199 were also included but did not show tolerance to mutation.

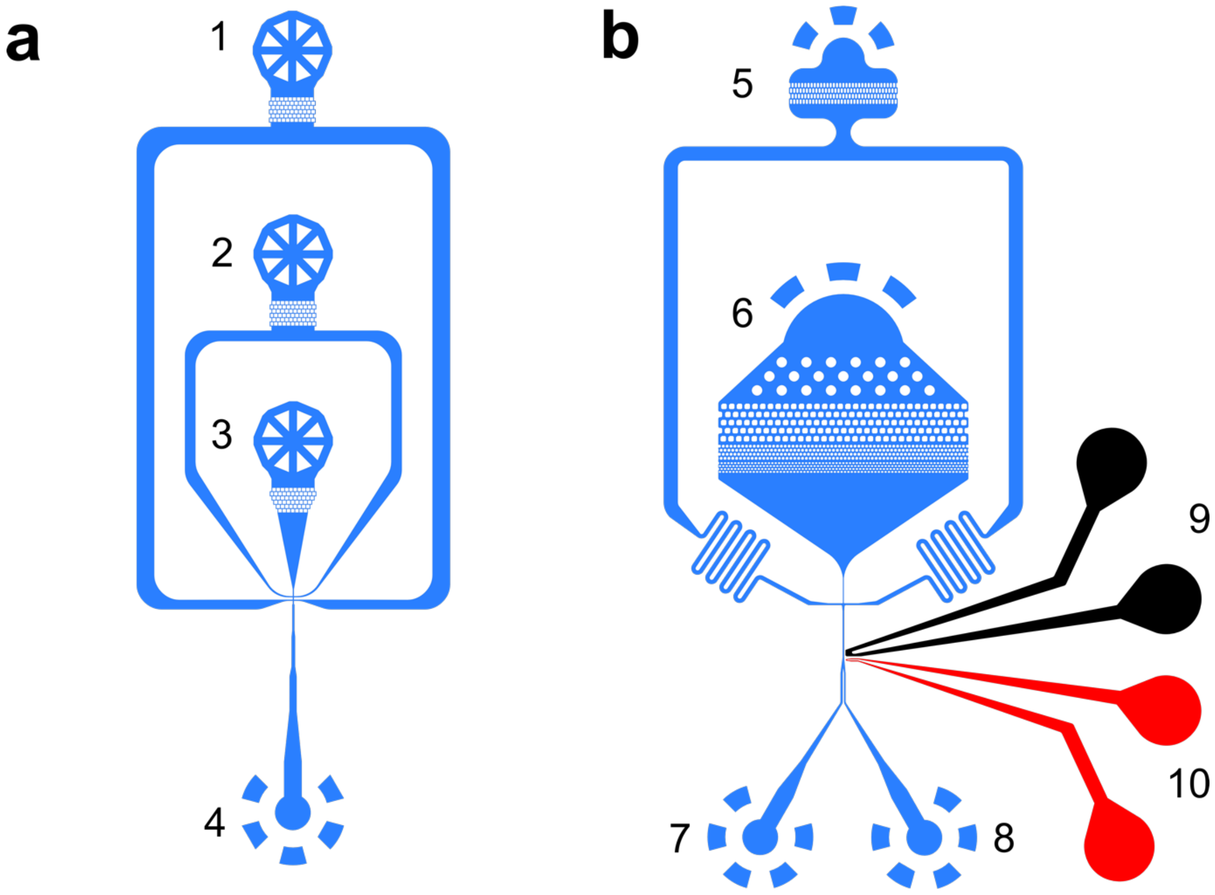

Figure S3: Design of microfluidic chips for droplet generation and sorting (off-line droplet incubation). (a) Flow-focussing chip (depth: 12 µm) for droplet generation with (1) oil/surfactant mixture inlet, (2) inlet for substrate/lysis agent mixture, (3) inlet for cell suspension, and (4) outlet for droplet collection. **(b)** Droplet sorting chip (depth: 21.5–23 µm) for fluorescence-activated droplet sorting with (5) inlet for spacing oil, (6) inlet for droplets, (7) waste outlet, (8) hit outlet, (9) ground electrode (+, black), and (10) signal electrode (–, red). This figure is adapted from Neun *et al.* 2019.[6] Copyright 2022 American Chemical Society.

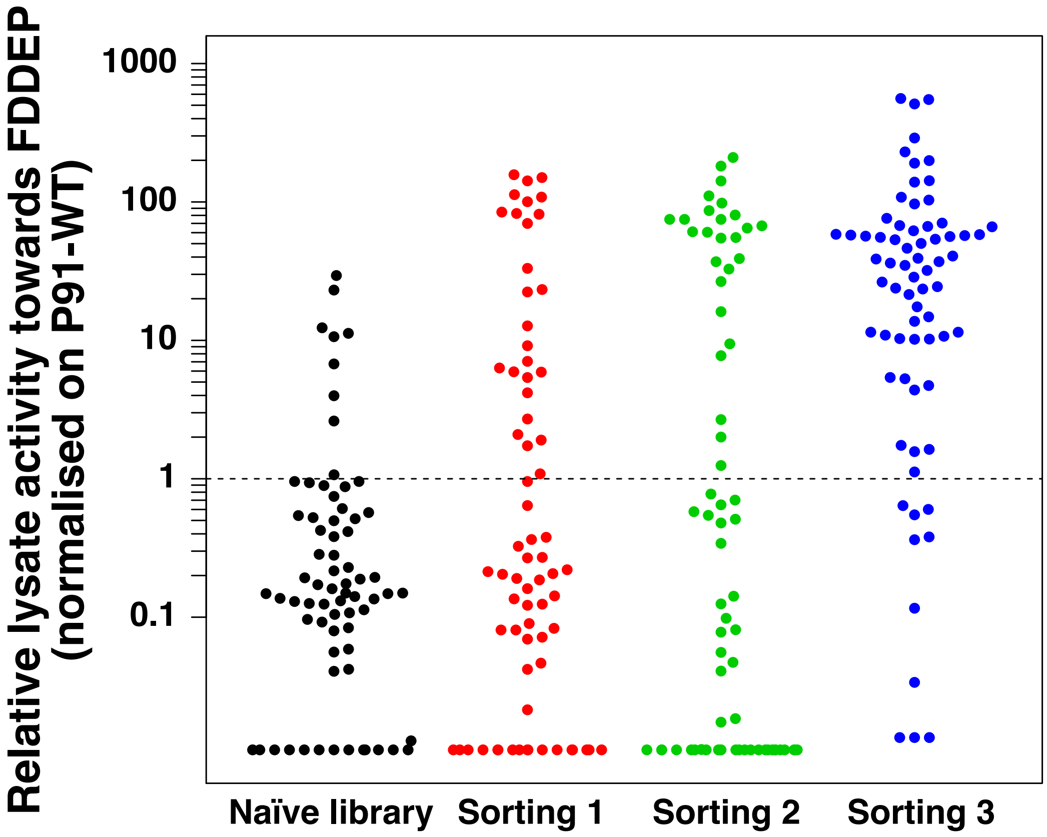

Figure S4: Lysate activity distribution of library P91-A (round 1) before and after successive droplet sorting. For each condition, 70 clones were randomly picked and assayed for lysate activity at 3 µM FDDEP in multititer plates. The dotted line indicates wildtype-level activity.

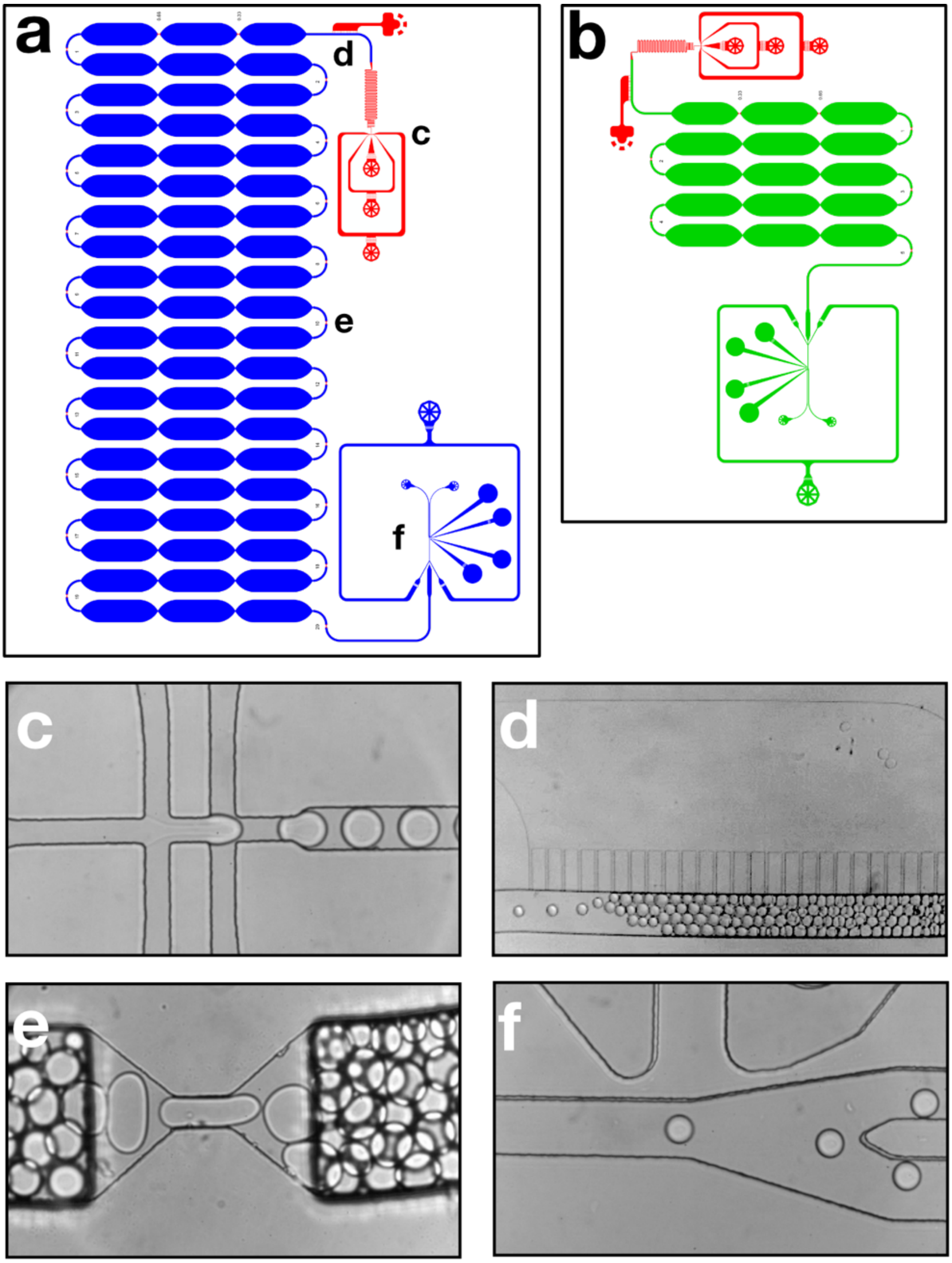

Figure S5: Design of microfluidic chips for on-chip droplet incubation. For short incubation times in evolution round 2, requiring on-chip incubation, an integrated chip was used, combining a flow-focussing module, a delay line, and a sorting module on a single device. The chips are two-layered such that the deeper delay line reduces backpressure. Red areas are 15 µm deep, green areas 28–29 µm, and blue areas 46–48 µm. **(a)** For initial stringency adjustments, a chip with a long delay line of 20 loops was used. **(b)** For library sorting, a shorter delay line consisting of five loops was used. **(c)** Monodisperse water-in-oil droplets are generated in a **flow-focusing nozzle**, co-encapsulating bacteria, the fluorogenic substrate and lysis agent. **(d)** An **oil extractor** ensures dense packing and equal incubation time for all droplets in the **(e) delay line chamber.** The delay line chambers possess mixing constrictions and shallow windows to monitor droplet fluorescence during incubation. **(f)** In the **sorting junction** droplets are electrophoretically sorted according to their fluorescence with up to kHz frequencies.

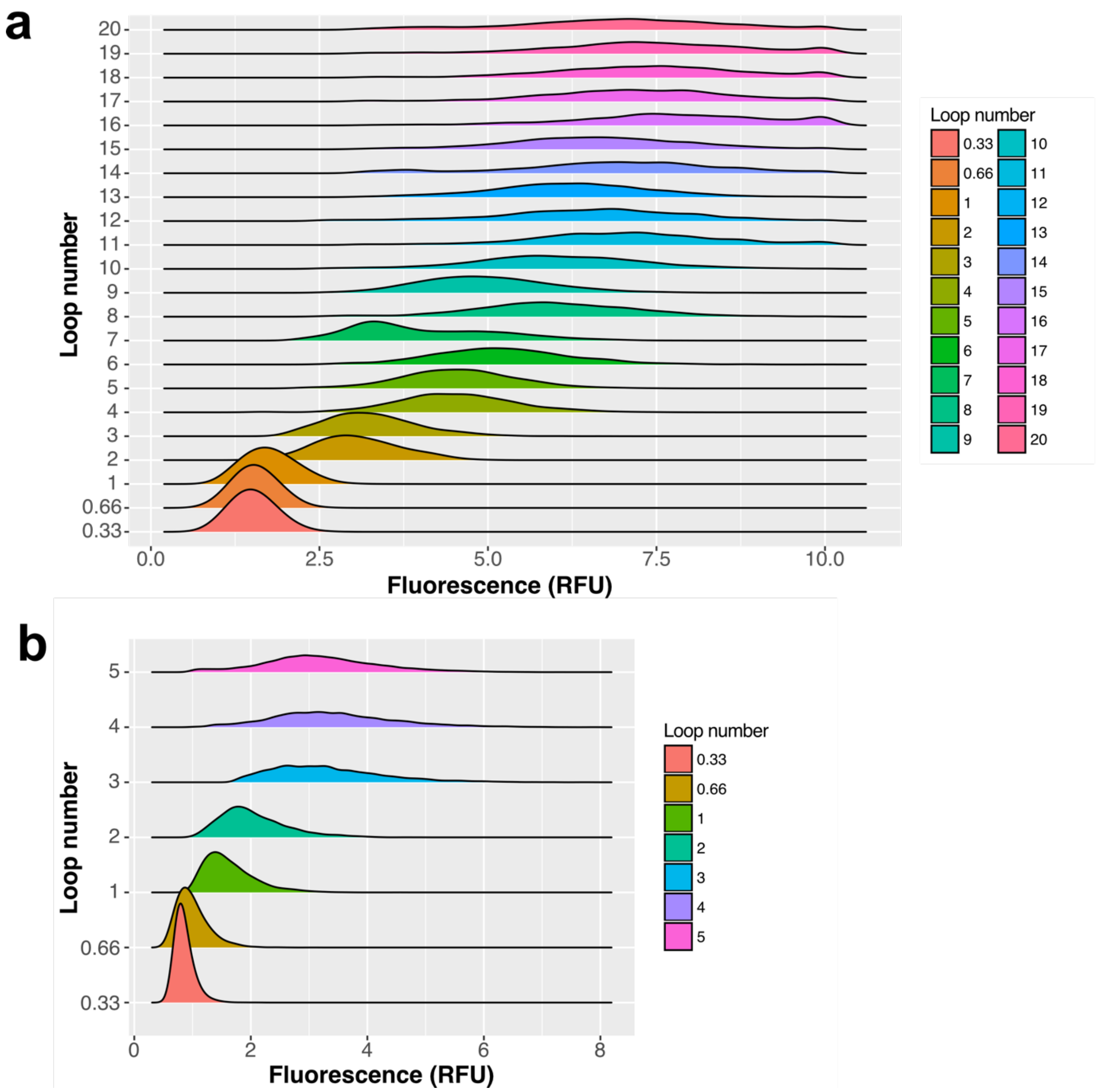

Figure S6: On-chip fluorescence measurements for the adjustment of reaction time and sorting stringency. Fluorescence distribution of 10 000 droplets in relative fluorescence units (RFU) at different points in the delay line. Fluorescence measurements were taken at the outer constrictions of the delay line loops (**Figure S5e**) in order to on follow the progress of the reaction in droplets. The loop number indicates the place of the constriction along the delay line where the laser was placed for measurements and is a proxy of reaction time. 0.33 and 0.66 refer to measurements at constrictions within the first loop of the delay line (after a third and two thirds of the first loop length, respectively). With the chosen flow rates, 20 loops correspond to ≈ 28 min in the long chip and five loops correspond to ≈ 4.5 min incubation time in the shorter and shallower library sorting chip. For initial stringency adjustments, a chip with a long delay line of 20 loops **(a)** was designed and cells expressing the parent variant for round 2, P91-R1, were encapsulated and incubated in the delay line. Fluorescence distribution increases linearly in the early loops and the reaction saturates in the later loops. With the aim to sort the library within the early linear phase of the reaction, this extent of reaction progress provided the basis for choosing five loops for the library sorting chip **(b).**

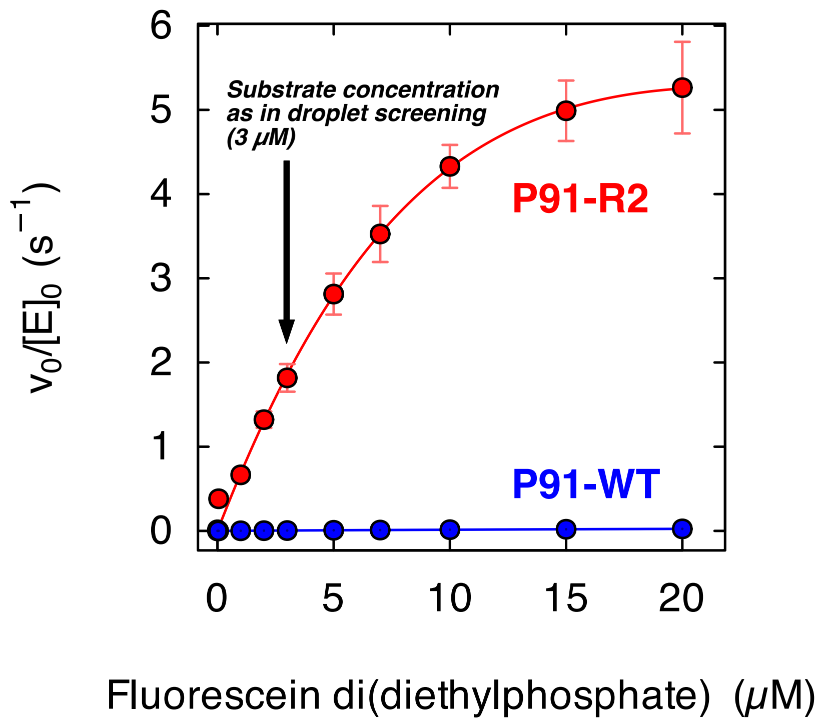

Figure S7: Close-up view of the low concentration range of the Michaelis-Menten plot (Figure 3) of wild-type and evolved P91 with FDDEP. The slope of the linear fit of measurements at low substrate concentrations (between 0 and 5 μM) allow a separate estimate of the k_{cat}/K_M improvement of P91-R2 (red) over P91-WT (blue). This approximation (at low [S], $\Delta v_0/\Delta[S] \approx k_{cat}/K_M$) yields an improvement of approx. 380-fold, confirming the approx. 360-fold improvement in k_{cat}/K_M calculated by nonlinear fit across the entire substrate concentration range. At the substrate concentration used in the droplet screening (3 μM, highlighted with black arrow), P91-R2 is approx. 450-fold improved. Error bars represent the standard error of three biological replicates.

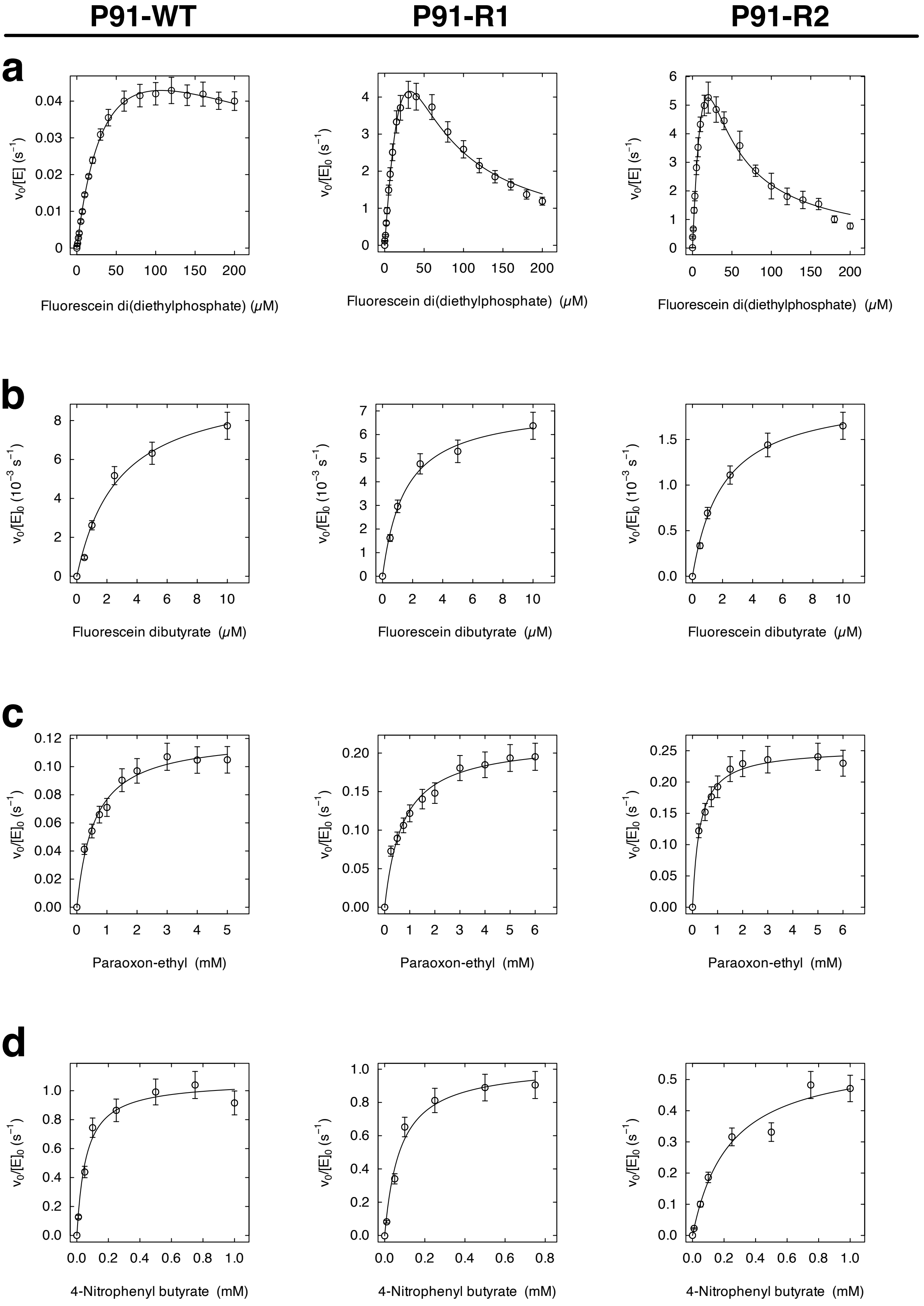

Figure S8: Michaelis-Menten plots of steady-state kinetics of P91-WT, P91-R1, and P91-R2 with **(a)** fluorescein di(diethylphosphate) **1, (b)** fluorescein dibutyrate **3**, **(c)** paraoxon-ethyl **2**, and **(d)** *p*-nitrophenyl butyrate **4.** Measured in 50 mM HEPES-NaOH, 150 mM NaCl, 1 mM TCEP, pH 8.0 at 25 °C. Error bars in Michelis-Menten plots of P91-WT and P91-R2 with fluorescein di(diethylphosphate) represent the standard error of three biological replicates. On other kinetics, a single measurement was taken and the error bars represent the typical error (9 %, estimated from the mean standard error between biological replicates of previous measurements).

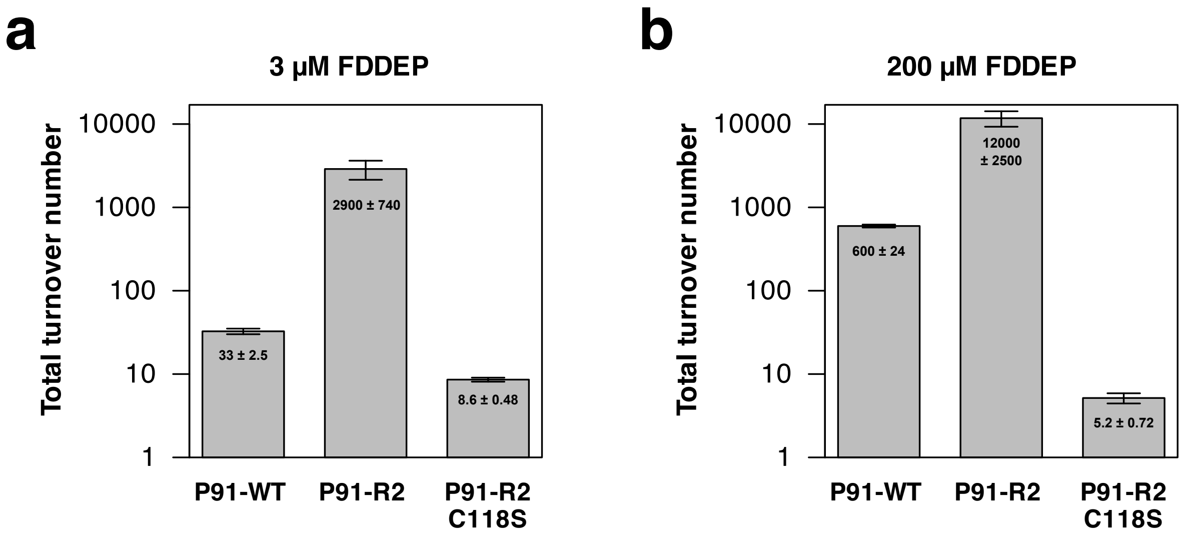

Figure S9: Total turnover numbers per enzyme active site of P91-WT, P91-R2, and P91-R2 Cys118Ser towards FDDEP. Turnover was measured with **(a)** 3 µM and **(b)** 200 µM FDDEP and an enzyme concentration of 0.5 nM (P91-WT), 0.5 pM (P91-R2), or 50 nM (P91-R2 C118S) in 50 mM HEPES-NaOH, 150 mM NaCl, 1 mM TCEP, pH 8.0 at 25 °C for 36 h. Plotted values are the mean of technical duplicates (P91-R2 at 200 µM FDDEP) or triplicates (all other measurements). Error bars represent the standard error of the mean.

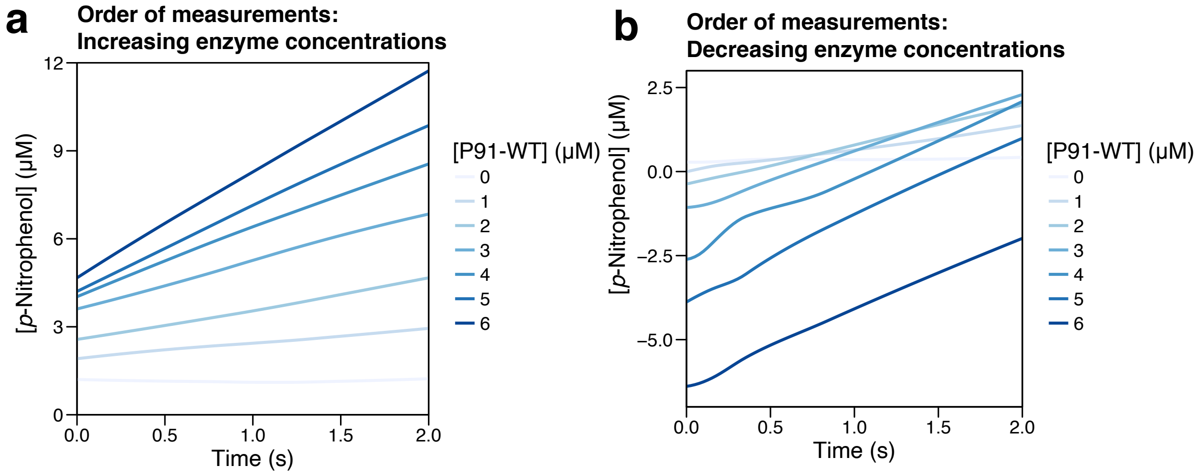

Figure S10: Wild-type P91 (containing a cysteine triad) does not show biphasic burst kinetics. Stopped-flow kinetic traces of P91-WT reacting with 2 mM paraoxon-ethyl, measured in 50 mM HEPES-NaOH, 150 mM NaCl, 1 mM TCEP, pH 8.0 at 25 °C at varying enzyme concentrations (0–6 µM, blue shades). Absorbance values are shown in units of corresponding concentration of released *p*-nitrophenol (PNP). **(a)** Time course measurements taken in order of *increasing* enzyme concentrations. **(b)** Time course measurements taken in order of *decreasing* enzyme concentrations. Although an absorbance offset is observable its amplitude does not depend on the enzyme concentration used but rather on the order of measurements (time-dependent or measurement-dependent, not enzyme concentration-dependent). The absorbance offset is therefore a measurement artifact (potential reasons could be, e.g., an untight seal or successive warm-up of the lamp), refuting the hypothesis that a very fast burst could be occurring in the dead time of the stopped-flow instrument (≈ 1–5 ms).

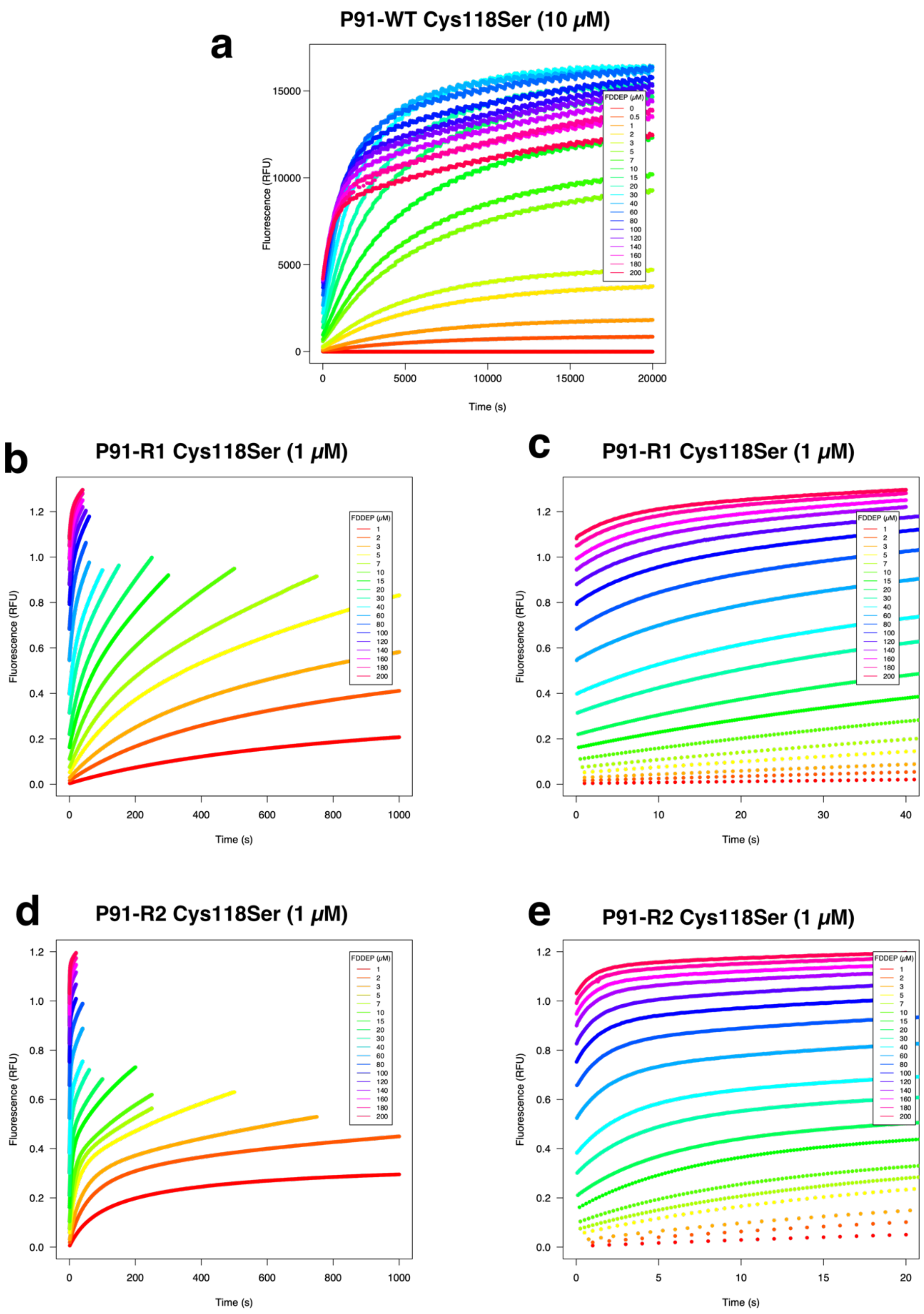

Figure S11: Stopped-flow reaction traces of all characterized P91 Cys118Ser variants with a concentration range of FDDEP (1–200 µM), measured in 50 mM HEPES-NaOH, 150 mM NaCl, 1 mM TCEP, pH 8.0 at 25 °C. Measurement time was varied with different substrate concentrations and was increased at low substrate concentrations according to reaction rate. **(a)** P91-WT Cys118Ser (10 µM). **(b)** P91-R1 Cys118Ser (1 µM), full time range. **(c)** P91-R1 Cys118Ser (1 µM), close-up of the initial 40 s. **d)** P91-R2 Cys118Ser (1 µM), full time range. **e)** P91-R1 Cys118Ser (1 µM), close-up of the initial 20 s.

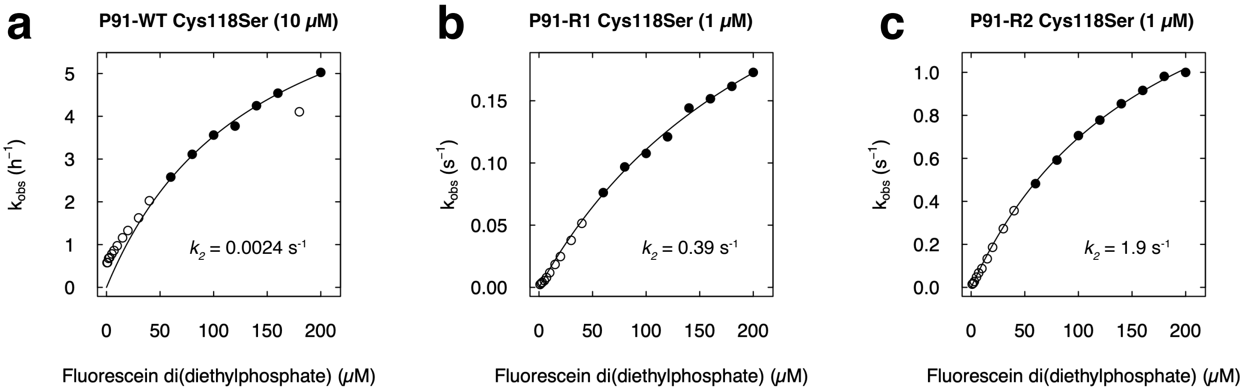

Figure S12: Determination of the phosphorylation rate k_2. The parameter k_{obs}, determined from exponential fit of the kinetic burst traces measured with the substrate FDDEP, was plotted against substrate concentration and fitted to a modified Michaelis-Menten equation as described in the Methods section. Only substrate concentrations of ≥ 60 µM FDDEP (where substrate concentration >> enzyme concentration) were considered for the fit (filled dots). Phosphorylation rates and standard errors of the fit were determined as **(a)** P91-WT Cys118Ser: (2.4 ± 0.10) · 10^{-3} s^{-1}. **(b)** P91-R1 Cys118Ser: (3.9 ± 0.32) · 10^{-1} s^{-1}. **(c)** P91-R2 Cys118Ser: (1.9 ± 0.067) s^{-1}. As the curves do not reach saturation due to low substrate solubilities, the values determined for k_2 may contain an error larger than that of the fit.

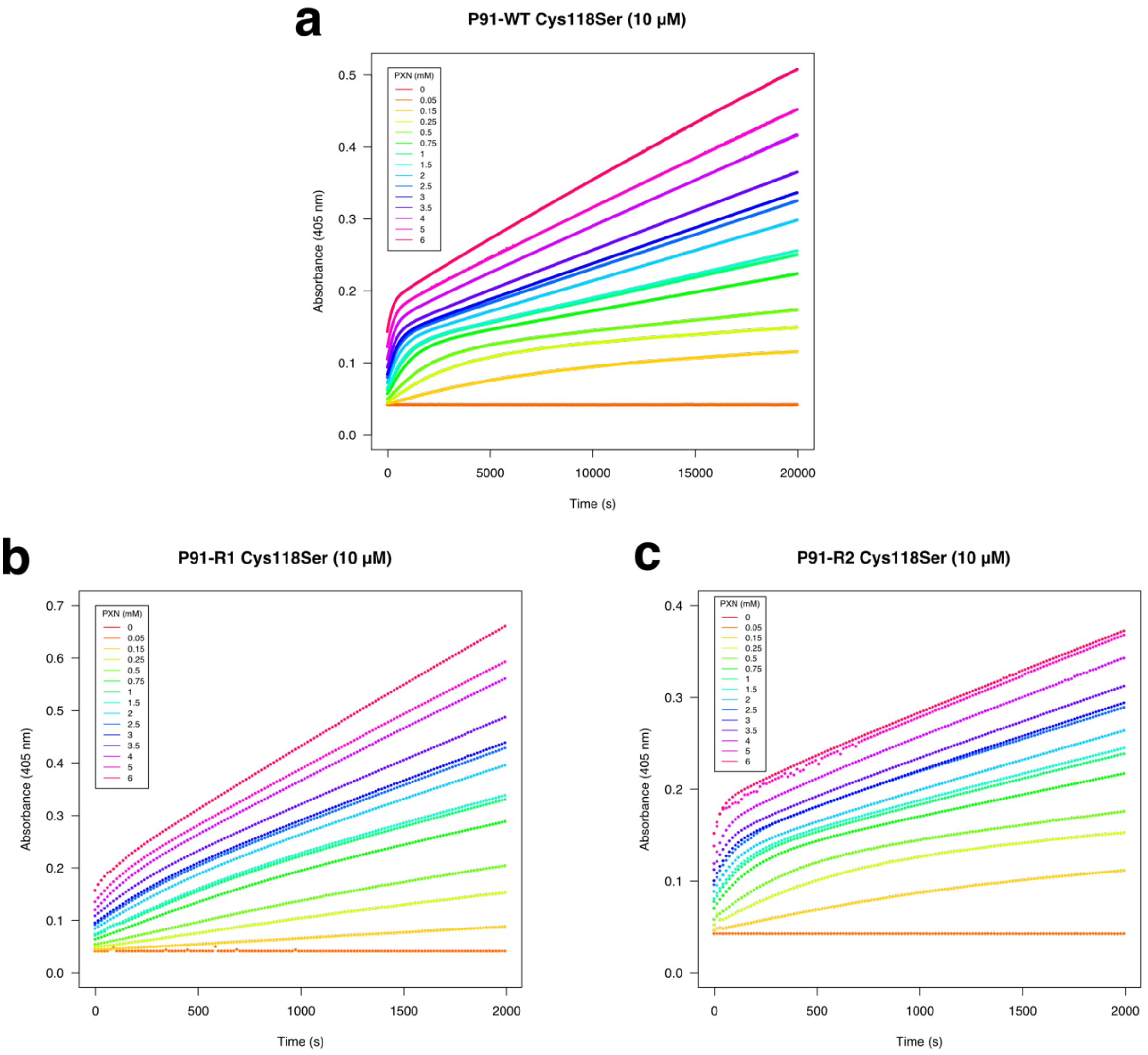

Figure S13: Burst traces of all characterized P91 Cys118Ser variants with a concentration range of paraoxon-ethyl (0.05–6 mM), measured in 50 mM HEPES-NaOH, 150 mM NaCl, 1 mM TCEP, pH 8.0 at 25 °C. **(a)** P91-WT Cys118Ser (10 µM). **(b)** P91-R1 Cys118Ser (10 µM). **(c)** P91-R2 Cys118Ser (10 µM).

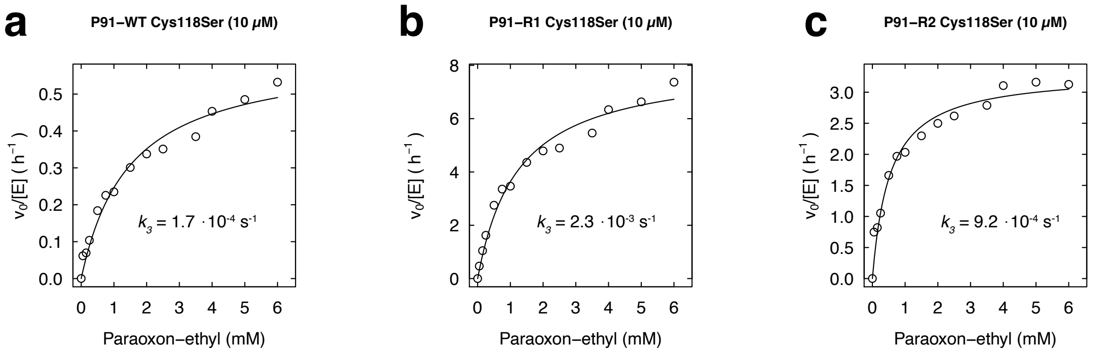

Figure S14: Determination of the de-phosphorylation rate k_3. The initial rate v_0 from the second phase of the burst kinetics with the substrate paraoxon-ethyl was fitted to the Michaelis-Menten equation to determine k_{cat} and thus k_3, as described in the Methods section. In the case of P91-R1 Cys118Ser, where the burst is less pronounced (probably due to a lower k_2 with the substate paraoxon), k_{cat} gives an upper boundary and thus k_3 might be lower. De-phosphorylation rates and standard error of the fit were determined as **(a)** P91-WT Cys118Ser: (1.7 ± 0.10) × 10^{-4} s^{-1}. **(b)** P91-R1 Cys118Ser: (2.3 ± 0.11) × 10^{-3} s^{-1}. **(c)** P91-R2 Cys118Ser: (9.2 ± 0.34) × 10^{-4} s^{-1}.

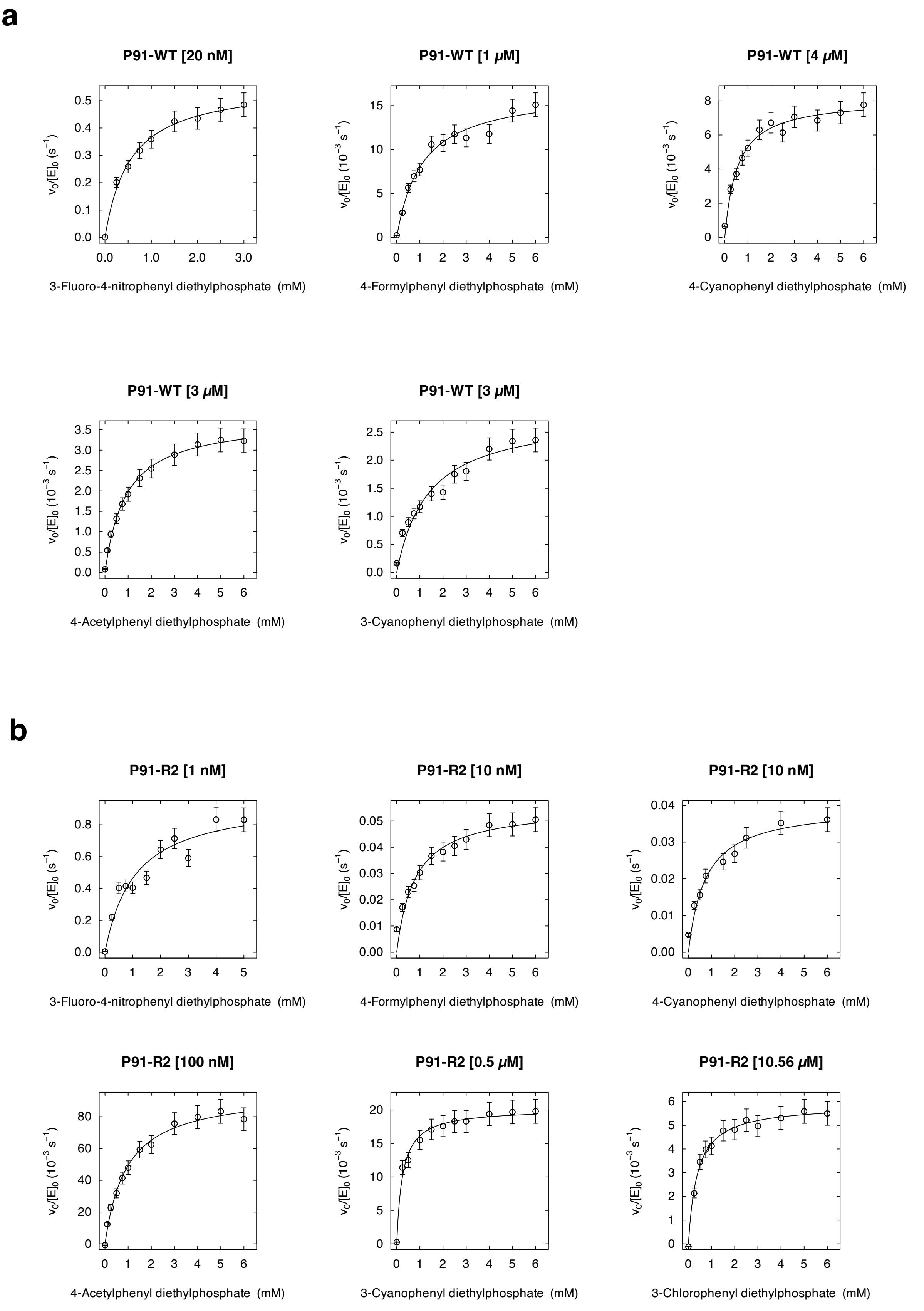

Figure S15: Michaelis-Menten plots for steady-state kinetics of (a) P91-WT and (b) P91-R2 with linear-free energy relationship substrates 5–10 (see axis labels), measured in 50 mM HEPES-NaOH, 150 mM NaCl, pH 8.0 at 25 °C. Measurement points represent a single measurement while the error bars represent the typical standard error of a biological triplicate from previous measurements.

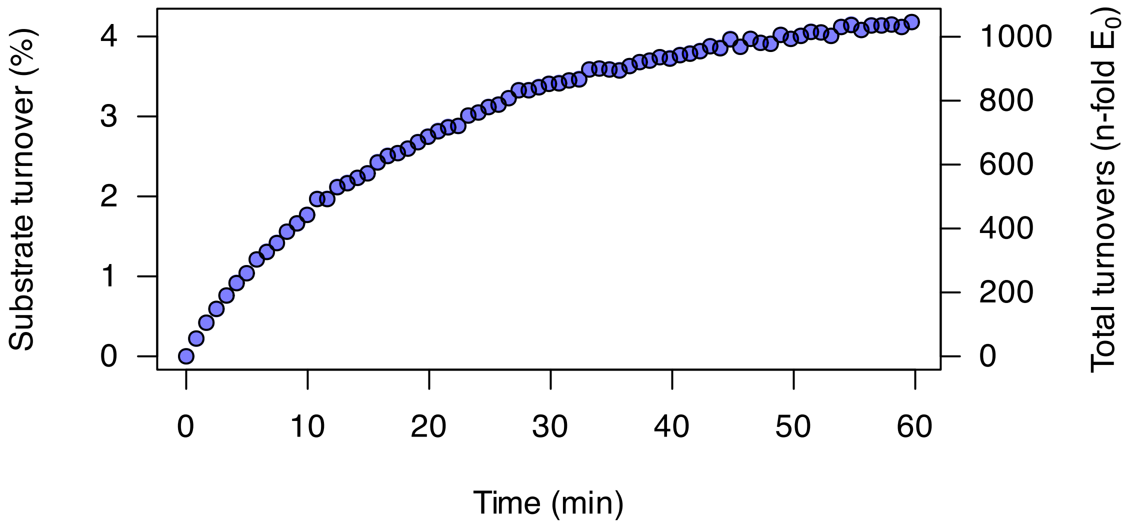

Figure S16: Example progress curve of FDDEP hydrolysis by the evolved variant P91-R2, measured in 50 mM HEPES-NaOH, 150 mM NaCl, pH 8.0 at 25 °C at a substrate concentration of 5 µM and an enzyme concentration of 0.2 nM. Early curvature is consistent with the existence of product inhibition. Since fluorescein does not inhibit the enzyme (**Figure S17**), either diethyl phosphate or fluorescein mono(diethylphosphate) is likely the inhibiting product.

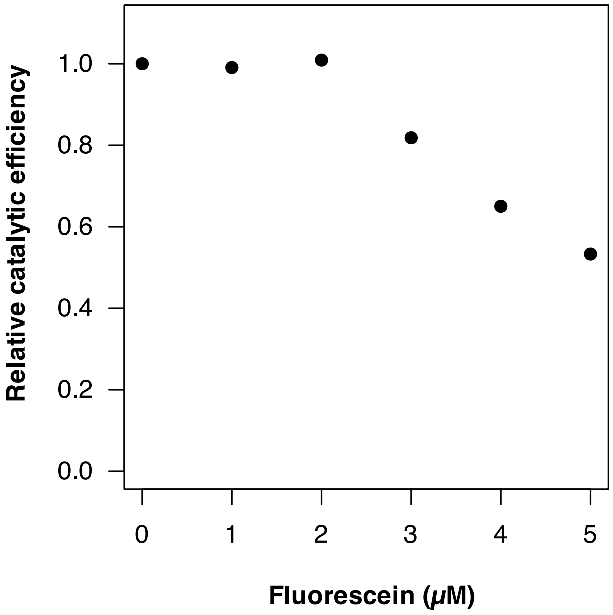

Figure S17: Inhibition of the P91-R2-catalysed hydrolysis of FDDEP by fluorescein. k_{cat}/K_M for FDDEP hydrolysis was measured at different concentrations of the reaction product fluorescein and normalized on activity without added fluorescein. For each measurement, k_{cat}/K_M was extrapolated from a linear fit to the low concentration range of a Michaelis-Menten plot where $v_0 \approx V_{max}/K_M$. Measurements were taken in 50 mM HEPES-NaOH, 150 mM NaCl, pH 8.0 at 25 °C at substrate concentrations of 0–5 µM and an enzyme concentration of 0.2 nM. The plot indicates no significant inhibition by fluorescein in the relevant concentration range. As the maximal fluorescein concentration reached in the measurements for Michaelis-Menten kinetics is ≈ 2 µM, the observed product inhibition (**Figure S16**) is likely caused by one of the other reaction products, diethyl phosphate or fluorescein mono(diethylphosphate).

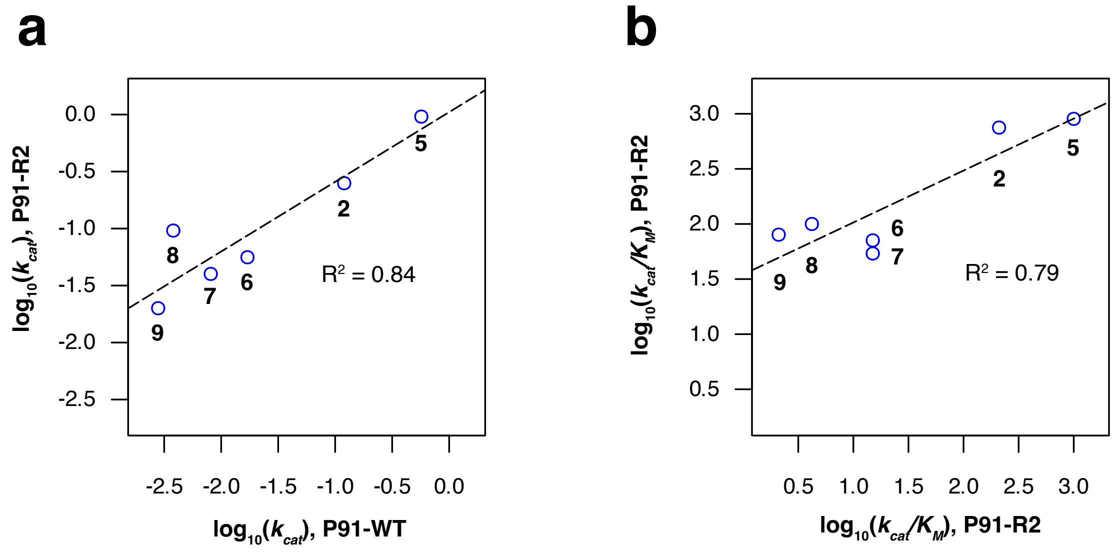

Figure S18: Quality control of the Brønsted analysis for substrate-specific binding effects. The kinetic constants **(a)** k_{cat} and **(b)** k_{cat}/K_M from the linear free-energy relationship analysis of P91-WT were plotted against those of P91-R2 and fitted to a linear function. The linearity of the fit with $R^2 = 0.84$ (k_{cat}) and $R^2 = 0.79$ (k_{cat}/K_M) indicate that there are no significant substrate-specific binding effects. Only substrates 4-acetylphenyl diethylphosphate (substrate **8**, for k_{cat}) and 4-cyanophenyl diethylphosphate (substrate 7, for k_{cat}/K_M) are slight outliers. The overall linearity of the fit validates the assumption that differences in kinetic constants between the substrates are largely due to differences in the leaving group pK_a and only to a small extent due to idiosyncratic differences in binding.

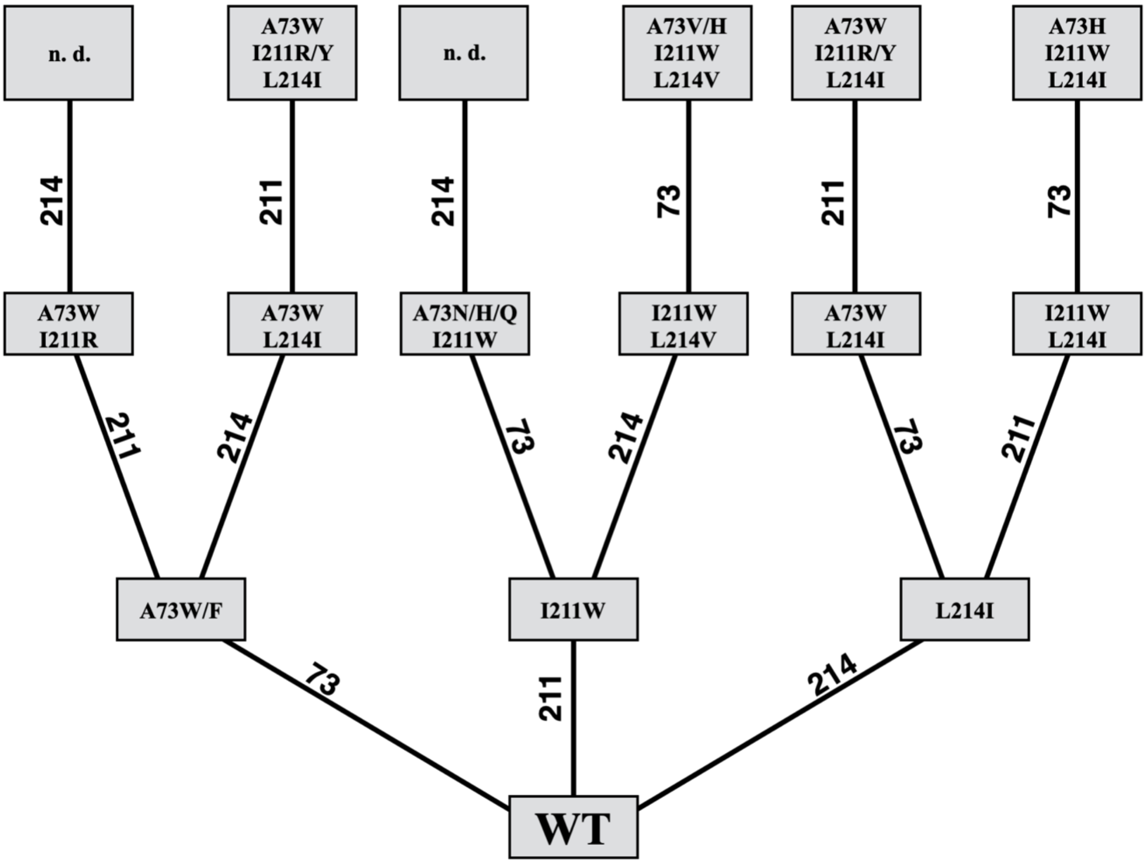

Figure S19: Iterative saturation mutagenesis (ISM) of P91 at the three positions A73, I211 and L214. Each position was randomized and screened for activity towards substrate **1** (FDDEP) in 96-well plates. Edges represent a screening campaign with the respective randomized residue indicated on the line, boxes represent the best identified mutant which served as starting variant for the next randomisation. In some cases, results were slightly ambiguous due to approximately equally improved mutants. n.d.; not determined.

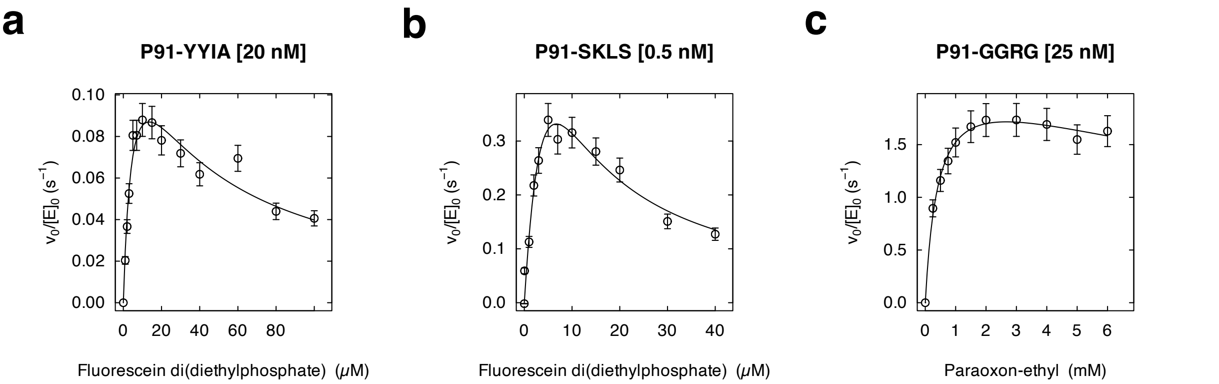

Figure S20: Michaelis-Menten plots for steady-state kinetics of alternative screening hits (a) P91-YYIA and **(b)** P91-SKLS with fluorescein di(diethylphosphate) **1** and **(c)** P91-GGRG with paraoxon-ethyl **2**, measured in 50 mM HEPES-NaOH, 150 mM NaCl, 1 mM TCEP, pH 8.0 at 25 °C. Error bars represent the typical error from biological replicates (9 %). Kinetic parameters are reported in **Table S7**.

3. Kinetic data and comparisons

Table S1: Steady-state catalytic parameters for P91-WT, P91-R1, and P91-R2, measured in 50 mM HEPES-NaOH, 150 mM NaCl, 1 mM TCEP, pH 8.0 at 25 °C.

	P91-WT[b]				P91-R1				P91-R2			
**Substrate**	k_{cat} (s^{-1})	K_M (μM)	K_i (μM)	k_{cat}/K_M ($M^{-1}\cdot s^{-1}$)	k_{cat} (s^{-1})	K_M (μM)	K_i (μM)	k_{cat}/K_M ($M^{-1}\cdot s^{-1}$)	k_{cat} (s^{-1})	K_M (μM)	K_i (μM)	k_{cat}/K_M ($M^{-1}\cdot s^{-1}$)
**FDDEP**	0.081	46	290	$1.8 \cdot 10^3$	37[a]	120[a]	7.9	$3.0 \cdot 10^5$	150[a]	290[a]	7.1	$6.5 \cdot 10^5$
**Fluorescein dibutyrate**	0.010	2.8	--	$3.6 \cdot 10^3$	0.0072	1.5	--	$4.8 \cdot 10^3$	0.0020	2.0	--	$1.0 \cdot 10^3$
**Paraoxon-ethyl**	0.12	580	--	$2.1 \cdot 10^2$	0.22	720	--	$3.0 \cdot 10^2$	0.25	290	--	$7.5 \cdot 10^2$
***p*-Nitrophenyl butyrate**	1.1	58	--	$1.9 \cdot 10^4$	1.0	77	--	$1.3 \cdot 10^4$	0.58	230	--	$2.5 \cdot 10^3$

[a] Due to strong substrate inhibition, estimates of k_{cat} and K_M are large extrapolations; only k_{cat}/K_M can be regarded as precise (see Michaelis-Menten plot, Figure **S8a**).

[b] Values for FDDEP and paraoxon-ethyl differ slightly from previously published values[13], presumably due to differences in affinity tag and buffer conditions.

Table S2: P92-R2 rivals the efficiencies of engineered and naturally evolved metal-dependent phosphotriesterases. Comparison of kinetic parameters of promiscuous, engineered, or naturally evolved phosphotriesterases from different protein superfamilies: ABH, α/β-hydrolases; BP, β-propellers; MBL, metallo-β-lactamases; AH, amidohydrolases. Substrates between enzymes differ in leaving group but are all diethyl-substituted phosphotriesters. n.d., not determined; n.a., not applicable.

Label in figure 4	Enzyme	Protein superfamily	Catalytic mechanism	Organism of origin	Substrate	Leaving group (LG)	$pK_{a,\mathrm{LG}}$	k_{cat}/K_M ($10^3 \cdot M^{-1} \cdot s^{-1}$)	Relative increase in k_{cat}/K_M	Rounds[a]	Reference
a	P91-WT	ABH	nucleophilic	metagenomic	Fluorescein di(diethylphosphate)	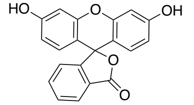	6.7	2.0	n.a.	n.a.	this work
b	P91-R2	ABH	nucleophilic	metagenomic	Fluorescein di(diethylphosphate)	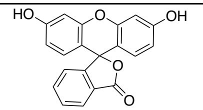	6.7	780	400	2	this work
c	BChE G117H	ABH	nucleophilic	*Homo sapiens*	Echothiophate	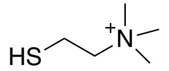	7.7	0.45	n.d.[b]	1[b]	[19]
d	*Lc*αE7 G137D	ABH	nucleophilic	*Lucilia cuprina*	4-Methylumbelliferyl diethylphosphate	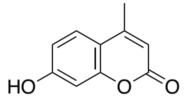	7.8	0.16	n.a.	n.a.	[17]
e	AiiA-WT	MBL	metal	*Bacillus thuringiensis*	Paraoxon-ethyl	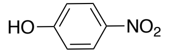	7.1	0.51	n.a.	n.a.	[26]
	AiiA-WT	MBL	metal	*Bacillus thuringiensis*	Parathion-methyl[c]	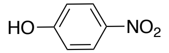	7.1	70	n.a.	n.a.	[26]
f	AiiA-R6	MBL	metal	*Bacillus thuringiensis*	Paraoxon-ethyl	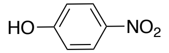	7.1	540	1100	6	[26]
g	MPH	MBL	metal	*Pseudomonas sp. WBC-3*	Paraoxon-ethyl	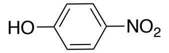	7.1	16	n.a.	n.a.	[26]
h	rePON1-G3C9	BP	metal	*Oryctolagus cuniculus*[d]	DEPCyc	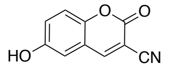	6.3[h]	11	n.a.	n.a.	[27]
i	rePON1-G3C9-3.2PC	BP	metal	*Oryctolagus cuniculus*[d]	DEPCyc	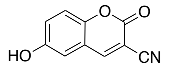	6.3[h]	1,400	130	6	[28]
j	*Dr*PLL-WT	AH	metal	*Deinococcus radiodurans*	Paraoxon-ethyl	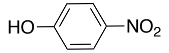	7.1	0.029	n.a.	n.a.	[29]

k	*Dr*PLL.10	AH	metal	*Deinococcus radiodurans*	Paraoxon-ethyl	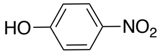	7.1	20	690	10	[29]
	*Dr*PLL.10	AH	metal	*Deinococcus radiodurans*	O-isopbutyl-O-4-nitrophenyl-R_P-methylphosphonate[e]	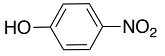	7.1	1100	37000	10	[29]
l	*Bd*PTE	AH	metal	*Brevundimonas diminuta*	Paraoxon-ethyl	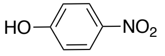	7.1	120000	n.a.	n.a.	[29]
	BdPTE	AH	metal	*Brevundimonas diminuta*	Paraoxon-ethyl	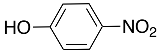	7.1	180000	1.8[f]	1	[30]
	Dr-OPH w.t.	AH	metal	*Deinococcus radiodurans*	Paraoxon-ethyl	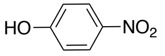	7.1	0.0014	n.a.	n.a.	[31]
	Dr-OPH D71G/E101G/V235L	AH	metal	*Deinococcus radiodurans*	Paraoxon-ethyl	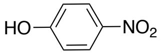	7.1	0.77	560	3	[31]
	*Gka*P-PLL WT	AH	metal	*Geobacillus kaustophilus*	Paraoxon-ethyl	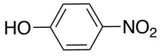	7.1	0.13	n.a.	n.a.	[32,33]
	*Gka*P-PLL ML7-B6	AH	metal	*Geobacillus kaustophilus*	Paraoxon-ethyl	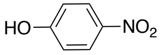	7.1	79	610	6	[32,33]
	OpdA WT	AH	metal	*Agrobacterium radiobacter*	Z-Chlorfenvinphos	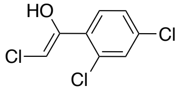	7.3[h]	0.0096	n.a.	n.a.	[34]
	OpdA W131H/F132A	AH	metal	*Agrobacterium radiobacter*	Z-Chlorfenvinphos	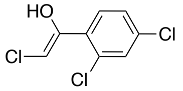	7.3[h]	4.6	480	2[g]	[34]

a) Number of laboratory evolution rounds, meaning successive iterations of diversification and screening.
b) A single substitution was rationally engineered into the wild-type precursor which has no detectable activity.
c) Substrate with highest final activity in this evolution campaign.
d) Engineered rabbit gene with contributions from the respective human, mouse, and rat genes through DNA shuffling during directed evolution.
e) Substrate with highest final activity and highest relative activity increase in this evolution campaign.
f) 1.8-fold increase in k_{cat}/K_M as compared to a wild-type k_{cat}/K_M of $9.9 \cdot 10^7$ $M^{-1} \cdot s^{-1}$, as measured in this study. In addition, the selected *Bd*PTE variant displayed a 63-fold increase in k_{cat}.
g) Two steps of rational design
h) pK_a predicted with XGBoost[35] (http://pka.luoszgroup.com/prediction)

Table S3: Microscopic rate constants for the formation (k_2) and the breakdown (k_3) of the covalent intermediate with nucleophile-exchanged variants. k_2 was determined with the substrate FDDEP, k_3 was determined with the substrate paraoxon-ethyl (PXN).

Enzyme variant	k_2 (s^{-1})	k_3 (s^{-1})	k_3/K_M (*PXN*, M^{-1} s^{-1})
P91-WT Cys118Ser	$2.4 \cdot 10^{-3}$	$1.7 \cdot 10^{-4}$	0.11
P91-R1 Cys118Ser	$3.9 \cdot 10^{-1}$	$2.3 \cdot 10^{-3}$	1.8
P91-R2 Cys118Ser	$1.9 \cdot 10^{0}$	$9.2 \cdot 10^{-4}$	1.7

Table S4: Properties of phosphotriester substrates (paraoxon-ethyl derivatives) used for the linear free-energy relationships.

Substrate	Leaving group	pK_a	Detection wavelength (nm)	Extinction coefficient (M^{-1})
5	3-fluoro-4-nitrophenol	5.94	390	10473.2
2	4-nitrophenol	7.14	405	10038.1
6	4-hydroxybenzaldehyde	7.66	330	12483.8
7	4-cyanophenol	7.95	275	6721.7
8	4-hydroxyacetophenone	8.05	320	7242.1
9	3-cyanophenol	8.61	295	1246.2
10	3-chlorophenol	9.12	276	796.0

Table S5: Steady-state catalytic parameters for linear free-energy relationship of P91-WT, measured in 50 mM HEPES-NaOH, 150 mM NaCl, 1 mM TCEP, pH 8.0.

Substrate	pK_a of leaving group	Enzyme concentration in the measurement	k_{cat} (s^{-1})	K_M (mM)	k_{cat}/K_M ($M^{-1}\cdot s^{-1}$)
5	5.94	20 nM	0.57	0.55	1000
2	7.14	0.5 µM	0.12	0.58	210
6	7.66	1 µM	0.017	1.1	15
7	7.95	4 µM	0.0081	0.54	15
8	8.05	3 µM	0.0038	0.9	4.2
9	8.61	3 µM	0.0028	1.4	2.1
10	9.12	12.56 µM	no turnover could be detected		

Table S6: Steady-state catalytic parameters for linear free-energy relationship of P91-R2, measured in 50 mM HEPES-NaOH, 150 mM NaCl, 1 mM TCEP, pH 8.0.

Substrate	pK_a of leaving group	Enzyme concentration in the measurement	k_{cat} (s^{-1})	K_M (mM)	k_{cat}/K_M ($M^{-1}\cdot s^{-1}$)
5	5.94	1 nM	0.96	1.10	900
2	7.14	500 nM	0.25	0.29	750
6	7.66	10 nM	0.056	0.79	71
7	7.95	10 nM	0.04	0.74	54
8	8.05	100 nM	0.096	0.94	100
9	8.61	500 nM	0.02	0.25	80
10	9.12	10.56 µM	0.0059	0.39	15

Table S7: Steady-state catalytic parameters of other P91 variants identified in round 2 for phosphotriester hydrolysis, measured in 50 mM HEPES-NaOH, 150 mM NaCl, 1 mM TCEP, pH 8.0

at 25 °C. Enzyme concentrations were: 20 nM for P91-YYIA, 0.5 nM for P91-SKSL and 25 nM for P91-GGRG. Due to strong substrate inhibition, estimates of k_{cat} and K_M for both variants measured with FDDEP are extrapolations and only k_{cat}/K_M can be regarded as precise.

Enzyme variant	Mutations	Substrate	k_{cat} (s^{-1})	K_M (μM)	K_i (μM)	k_{cat}/K_M ($M^{-1}s^{-1}$)
P91-YYIA	Ala38Tyr, Ala73Tyr, Leu76Ile, Ile211Trp, Leu214Val	FDDEP	0.15	5.1	3.6	3.0×10^4
P91-SKLS	Ala38Ser, Ala73Lys, Leu76Leu, Ile211Trp, Leu214Val	FDDEP	1.1	7.9	5.7	1.4×10^5
P91-GGRG	Ala38Gly, Ala73Gly, Leu76Arg, Ala122Gly, Ile211Trp, Leu214Val	Paraoxon	2.3	430	17000	5.3×10^3

4. Sequences

4.1 Sequences of P91 variants and plasmid constructs

Notable codons are highlighted in bold, the coding region for the respective P91 variant is highlighted in green. XXX; randomized with degenerate codons NDT, VHG, TGG (22-codon trick).

```
>6xHis-P91-WT
MHHHHHHGGSMTARKVDYTDGATRCIGEFHWDEGKSGPRPGVVVFPEAFGLNDHAKERARRL
ADLGFAALAADMHGDAQVFDAASLSSTIQGYYGDRAHWRRRAQAALDALTAQPEVDGSKVAA
IGFCFGGATCLELARTGAPLTAIVTFHGGLLPEMAGDAGRIQSSVLVCHGADDPLVQDETMK
AVMDEFRRDKVDWQVLYLGNAVHSFTDPLAGSHGIPGLAYDATAEARSWTAMCNLFSELFG
```

```
>6xHis-P91-R1
MHHHHHHGGSMTARKVDYTDGATRCIGEFHWDEGKSGPRPGVVVFPEAFGLNDHAKERARRL
ADLGFAALAADMHGDAQVFDAASLSSTIQGYYGDRAHWRRRAQAALDALTAQPEVDGSKVAA
IGFCFGGATCLELARTGAPLTAIVTFHGGLLPEMAGDAGRIQSSVLVCHGADDPLVQDETMK
AVMDEFRRDKVDWQVLYLGNAVHSFTDPLAGSHGWPGVAYDATAEARSWTAMCNLFSELFG
```

```
>6xHis-P91-R2
MHHHHHHGGSMTARKVDYTDGATRCIGEFHWDEGKSGPRPGVVVFPELFGLNDHAKERARRL
ADLGFAALAADMHGDAQVFDEASVSSTIQGYYGDRAHWRRRAQAALDALTAQPEVDGSKVAA
IGFCFGGATCLELARTGAPLTAIVTFHGGLLPEMAGDAGRIQSSVLVCHGADDPLVQDETMK
AVMDEFRRDKVDWQVLYLGNAVHSFTDPLAGSHGWPGVAYDATAEARSWTAMCNLFSELFG
```

```
>pASK-IBA5plus_P91-A (library, round 1)
CTGGCAAGATTTTTTACGTAATAACGCTAAAAGTTTTAGATGTGCTTTACTAAGTCATCGCG
ATGGAGCAAAAGTACATTTAGGTACACGGCCTACAGAAAAACAGTATGAAACTCTCGAAAAT
CAATTAGCCTTTTTATGCCAACAAGGTTTTTCACTAGAGAATGCATTATATGCACTCAGCGC
AGTGGGGCATTTTACTTTAGGTTGCGTATTGGAAGATCAAGAGCATCAAGTCGCTAAAGAAG
AAAGGGAAACACCTACTACTGATAGTATGCCGCCATTATTACGACAAGCTATCGAATTATTT
GATCACCAAGGTGCAGAGCCAGCCTTCTTATTCGGCCTTGAATTGATCATATGCGGATTAGA
AAAACAACTTAAATGTGAAAGTGGGTCTTAAAAGCAGCATAACCTTTTTCCGTGATGGTAAC
TTCACTAGTTTAAAAGGATCTAGGTGAAGATCCTTTTTGATAATCTCATGACCAAAATCCCT
TAACGTGAGTTTTCGTTCCACTGAGCGTCAGACCCCGTAGAAAAGATCAAAGGATCTTCTTG
AGATCCTTTTTTTCTGCGCGTAATCTGCTGCTTGCAAACAAAAAAACCACCGCTACCAGCGG
TGGTTTGTTTGCCGGATCAAGAGCTACCAACTCTTTTTCCGAAGGTAACTGGCTTCAGCAGA
GCGCAGATACCAAATACTGTCCTTCTAGTGTAGCCGTAGTTAGGCCACCACTTCAAGAACTC
TGTAGCACCGCCTACATACCTCGCTCTGCTAATCCTGTTACCAGTGGCTGCTGCCAGTGGCG
ATAAGTCGTGTCTTACCGGGTTGGACTCAAGACGATAGTTACCGGATAAGGCGCAGCGGTCG
GGCTGAACGGGGGGTTCGTGCACACAGCCCAGCTTGGAGCGAACGACCTACACCGAACTGAG
ATACCTACAGCGTGAGCTATGAGAAAGCGCCACGCTTCCCGAAGGGAGAAAGGCGGACAGGT
ATCCGGTAAGCGGCAGGGTCGGAACAGGAGAGCGCACGAGGGAGCTTCCAGGGGGAAACGCC
TGGTATCTTTATAGTCCTGTCGGGTTTCGCCACCTCTGACTTGAGCGTCGATTTTTGTGATG
CTCGTCAGGGGGGCGGAGCCTATGGAAAAACGCCAGCAACGCGGCCTTTTTACGGTTCCTGG
CCTTTTGCTGGCCTTTTGCTCACATGACCCGACACCATCGAATGGCCAGATGATTAATTCCT
AATTTTTGTTGACACTCTATCATTGATAGAGTTATTTTACCACTCCCTATCAGTGATAGAGA
```

AAAGTGAAATGAATAGTTCGACAAAAATCTAGAAATAATTTTGTTTAACTTTAAGAAGGAGA
TATACAAATGGCTAGCTGGAGCCACCCGCAGTTCGAAAAAGGCGCCATGACAGCAAGAAAAG
TCGACTACACAGACGGTGCAACCCGCTGTATCGGTGAGTTTCATTGGGATGAAGGCAAGTCG
GGCCCGCGTCCCGGCGTGGTGGTCTTTCCCGAGGCTTTCGGCCTCAACGACCATGCCAAGGA
GCGCGCGCGGCGCCTTGCCGACCTCGGCTTTGCAGCCCTGGCGGCGGATATGCACGGAGACG
CCCAGGTTTTCGAT**NNK**GCGAGTCTCTCATCAACCATACAGGGCTACTACGGCGACCGCGCC
CACTGGCGACGTCGTGCGCAGGCAGCGCTCGATGCACTGACGGCACAGCCAGAGGTGGACGG
CAGCAAGGTGGCGGCCATCGGCTTTTGTTTCGGCGGTGCCACCTGCCTTGAACTGGCCCGCA
CAGGTGCGCCGCTGACCGCCATTGTCACCTTCCACGGCGGTTTGCTGCCGGAGATGGCAGGC
GATGCCGGACGGATCCAGTCCAGTGTTCTGGTGTGCCATGGCGCTGATGATCCGCTCGTACA
GGACGAAACCATGAAGGCCGTCATGGACGAGTTTCGTCGCGACAAGGTGGATTGGCAGGTGC
TCTACCTCGGAAATGCGGTACACAGTTTCACCGATCCACTCGCTGGCAGTCACGGC**NNK**CCC
GGG**NNK**GCCTATGACGCCACTGCCGAAGCCCGGTCGTGGACGGCCATGTGCAATCTGTTCAG
TGAACTGTTCGGCTGATGATATCTAACTAAGCTTGACCTGTGAAGTGAAAAATGGCGCACAT
TGTGCGACATTTTTTTTGTCTGCCGTTTACCGCTACTGCGTCACGGATCTCCACGCGCCCTG
TAGCGGCGCATTAAGCGCGGCGGGTGTGGTGGTTACGCGCAGCGTGACCGCTACACTTGCCA
GCGCCCTAGCGCCCGCTCCTTTCGCTTTCTTCCCTTCCTTTCTCGCCACGTTCGCCGGCTTT
CCCCGTCAAGCTCTAAATCGGGGGCTCCCTTTAGGGTTCCGATTTAGTGCTTTACGGCACCT
CGACCCCAAAAAACTTGATTAGGGTGATGGTTCACGTAGTGGGCCATCGCCCTGATAGACGG
TTTTTCGCCCTTTGACGTTGGAGTCCACGTTCTTTAATAGTGGACTCTTGTTCCAAACTGGA
ACAACACTCAACCCTATCTCGGTCTATTCTTTTGATTTATAAGGGATTTTGCCGATTTCGGC
CTATTGGTTAAAAAATGAGCTGATTTAACAAAAATTTAACGCGAATTTTAACAAAATATTAA
CGCTTACAATTTCAGGTGGCACTTTTCGGGGAAATGTGCGCGGAACCCCTATTTGTTTATTT
TTCTAAATACATTCAAATATGTATCCGCTCATGAGACAATAACCCTGATAAATGCTTCAATA
ATATTGAAAAAGGAAGAGTATGAGTATTCAACATTTCCGTGTCGCCCTTATTCCCTTTTTTG
CGGCATTTTGCCTTCCTGTTTTTGCTCACCCAGAAACGCTGGTGAAAGTAAAAGATGCTGAA
GATCAGTTGGGTGCACGAGTGGGTTACATCGAACTGGATCTCAACAGCGGTAAGATCCTTGA
GAGTTTTCGCCCCGAAGAACGTTTTCCAATGATGAGCACTTTTAAAGTTCTGCTATGTGGCG
CGGTATTATCCCGTATTGACGCCGGGCAAGAGCAACTCGGTCGCCGCATACACTATTCTCAG
AATGACTTGGTTGAGTACTCACCAGTCACAGAAAAGCATCTTACGGATGGCATGACAGTAAG
AGAATTATGCAGTGCTGCCATAACCATGAGTGATAACACTGCGGCCAACTTACTTCTGACAA
CGATCGGAGGACCGAAGGAGCTAACCGCTTTTTTGCACAACATGGGGGATCATGTAACTCGC
CTTGATCGTTGGGAACCGGAGCTGAATGAAGCCATACCAAACGACGAGCGTGACACCACGAT
GCCTGTAGCAATGGCAACAACGTTGCGCAAACTATTAACTGGCGAACTACTTACTCTAGCTT
CCCGGCAACAATTGATAGACTGGATGGAGGCGGATAAAGTTGCAGGACCACTTCTGCGCTCG
GCCCTTCCGGCTGGCTGGTTTATTGCTGATAAATCTGGAGCCGGTGAGCGTGGCTCTCGCGG
TATCATTGCAGCACTGGGGCCAGATGGTAAGCCCTCCCGTATCGTAGTTATCTACACGACGG
GGAGTCAGGCAACTATGGATGAACGAAATAGACAGATCGCTGAGATAGGTGCCTCACTGATT
AAGCATTGGTAGGAATTAATGATGTCTCGTTTAGATAAAAGTAAAGTGATTAACAGCGCATT
AGAGCTGCTTAATGAGGTCGGAATCGAAGGTTTAACAACCCGTAAACTCGCCCAGAAGCTAG
GTGTAGAGCAGCCTACATTGTATTGGCATGTAAAAAATAAGCGGGCTTTGCTCGACGCCTTA
GCCATTGAGATGTTAGATAGGCACCATACTCACTTTTGCCCTTTAGAAGGGGAAAG

>pASK-IBA5plus_P91-B (library, round 2)
CTGGCAAGATTTTTTACGTAATAACGCTAAAAGTTTTAGATGTGCTTTACTAAGTCATCGCG
ATGGAGCAAAAGTACATTTAGGTACACGGCCTACAGAAAAACAGTATGAAACTCTCGAAAAT
CAATTAGCCTTTTTATGCCAACAAGGTTTTTCACTAGAGAATGCATTATATGCACTCAGCGC
AGTGGGGCATTTTACTTTAGGTTGCGTATTGGAAGATCAAGAGCATCAAGTCGCTAAAGAAG
AAAGGGAAACACCTACTACTGATAGTATGCCGCCATTATTACGACAAGCTATCGAATTATTT
GATCACCAAGGTGCAGAGCCAGCCTTCTTATTCGGCCTTGAATTGATCATATGCGGATTAGA
AAAACAACTTAAATGTGAAAGTGGGTCTTAAAAGCAGCATAACCTTTTTCCGTGATGGTAAC
TTCACTAGTTTAAAAGGATCTAGGTGAAGATCCTTTTTGATAATCTCATGACCAAAATCCCT

TAACGTGAGTTTTCGTTCCACTGAGCGTCAGACCCCGTAGAAAAGATCAAAGGATCTTCTTG
AGATCCTTTTTTTCTGCGCGTAATCTGCTGCTTGCAAACAAAAAAACCACCGCTACCAGCGG
TGGTTTGTTTGCCGGATCAAGAGCTACCAACTCTTTTTCCGAAGGTAACTGGCTTCAGCAGA
GCGCAGATACCAAATACTGTCCTTCTAGTGTAGCCGTAGTTAGGCCACCACTTCAAGAACTC
TGTAGCACCGCCTACATACCTCGCTCTGCTAATCCTGTTACCAGTGGCTGCTGCCAGTGGCG
ATAAGTCGTGTCTTACCGGGTTGGACTCAAGACGATAGTTACCGGATAAGGCGCAGCGGTCG
GGCTGAACGGGGGGTTCGTGCACACAGCCCAGCTTGGAGCGAACGACCTACACCGAACTGAG
ATACCTACAGCGTGAGCTATGAGAAAGCGCCACGCTTCCCGAAGGGAGAAAGGCGGACAGGT
ATCCGGTAAGCGGCAGGGTCGGAACAGGAGAGCGCACGAGGGAGCTTCCAGGGGGAAACGCC
TGGTATCTTTATAGTCCTGTCGGGTTTCGCCACCTCTGACTTGAGCGTCGATTTTTGTGATG
CTCGTCAGGGGGGCGGAGCCTATGGAAAAACGCCAGCAACGCGGCCTTTTTACGGTTCCTGG
CCTTTTGCTGGCCTTTTGCTCACATGACCCGACACCATCGAATGGCCAGATGATTAATTCCT
AATTTTTGTTGACACTCTATCATTGATAGAGTTATTTTACCACTCCCTATCAGTGATAGAGA
AAAGTGAAATGAATAGTTCGACAAAAATCTAGAAATAATTTTGTTTAACTTTAAGAAGGAGA
TATACAAATGGCTAGCTGGAGCCACCCGCAGTTCGAAAAAGGCGCCATGACAGCAAGAAAAG
TCGACTACACAGACGGTGCAACCCGCTGTATCGGTGAGTTTCATTGGGATGAAGGCAAGTCG
GGCCCGCGTCCCGGCGTGGTGGTCTTTCCCGAG**XXX**TTCGGCCTCAACGACCATGCCAAGGA
GCGCGCGCGGCGCCTTGCCGACCTCGGCTTTGCAGCCCTGGCGGCGGATATGCACGGAGACG
CCCAGGTTTTCGAT**XXX**GCGAGT**XXX**TCATCAACCATACAGGGCTACTACGGCGACCGCGCC
CACTGGCGACGTCGTGCGCAGGCAGCGCTCGATGCACTGACGGCACAGCCAGAGGTGGACGG
CAGCAAGGTGGCGGCCATCGGCTTTTGTTTCGGCGGT**XXX**ACCTGCCTTGAACTGGCCCGCA
CAGGTGCGCCGCTGACCGCCATTGTCACCTTCCACGGCGGTTTGCTGCCGGAGATGGCAGGC
GATGCCGGACGGATCCAGTCCAGTGTTCTGGTGTGCCATGGCGCTGATGATCCGCTCGTACA
GGACGAAACCATGAAGGCCGTCATGGACGAGTTTCGTCGCGACAAGGTGGATTGGCAGGTGC
TCTACCTCGGAAATGCGGTACACAGTTTCACCGATCCACTCGCTGGCAGTCACGGC**TGG**CCC
GGG**GTT**GCCTATGACGCCACTGCCGAAGCCCGGTCGTGGACGGCCATGTGCAATCTGTTCAG
TGAACTGTTCGGCTGATGATATCTAACTAAGCTTGACCTGTGAAGTGAAAAATGGCGCACAT
TGTGCGACATTTTTTTTGTCTGCCGTTTACCGCTACTGCGTCACGGATCTCCACGCGCCCTG
TAGCGGCGCATTAAGCGCGGCGGGTGTGGTGGTTACGCGCAGCGTGACCGCTACACTTGCCA
GCGCCCTAGCGCCCGCTCCTTTCGCTTTCTTCCCTTCCTTTCTCGCCACGTTCGCCGGCTTT
CCCCGTCAAGCTCTAAATCGGGGGCTCCCTTTAGGGTTCCGATTTAGTGCTTTACGGCACCT
CGACCCCAAAAAACTTGATTAGGGTGATGGTTCACGTAGTGGGCCATCGCCCTGATAGACGG
TTTTTCGCCCTTTGACGTTGGAGTCCACGTTCTTTAATAGTGGACTCTTGTTCCAAACTGGA
ACAACACTCAACCCTATCTCGGTCTATTCTTTTGATTTATAAGGGATTTTGCCGATTTCGGC
CTATTGGTTAAAAAATGAGCTGATTTAACAAAAATTTAACGCGAATTTTAACAAAATATTAA
CGCTTACAATTTCAGGTGGCACTTTTCGGGGAAATGTGCGCGGAACCCCTATTTGTTTATTT
TTCTAAATACATTCAAATATGTATCCGCTCATGAGACAATAACCCTGATAAATGCTTCAATA
ATATTGAAAAAGGAAGAGTATGAGTATTCAACATTTCCGTGTCGCCCTTATTCCCTTTTTTG
CGGCATTTTGCCTTCCTGTTTTTGCTCACCCAGAAACGCTGGTGAAAGTAAAAGATGCTGAA
GATCAGTTGGGTGCACGAGTGGGTTACATCGAACTGGATCTCAACAGCGGTAAGATCCTTGA
GAGTTTTCGCCCCGAAGAACGTTTTCCAATGATGAGCACTTTTAAAGTTCTGCTATGTGGCG
CGGTATTATCCCGTATTGACGCCGGGCAAGAGCAACTCGGTCGCCGCATACACTATTCTCAG
AATGACTTGGTTGAGTACTCACCAGTCACAGAAAAGCATCTTACGGATGGCATGACAGTAAG
AGAATTATGCAGTGCTGCCATAACCATGAGTGATAACACTGCGGCCAACTTACTTCTGACAA
CGATCGGAGGACCGAAGGAGCTAACCGCTTTTTTGCACAACATGGGGGATCATGTAACTCGC
CTTGATCGTTGGGAACCGGAGCTGAATGAAGCCATACCAAACGACGAGCGTGACACCACGAT
GCCTGTAGCAATGGCAACAACGTTGCGCAAACTATTAACTGGCGAACTACTTACTCTAGCTT
CCCGGCAACAATTGATAGACTGGATGGAGGCGGATAAAGTTGCAGGACCACTTCTGCGCTCG
GCCCTTCCGGCTGGCTGGTTTATTGCTGATAAATCTGGAGCCGGTGAGCGTGGCTCTCGCGG
TATCATTGCAGCACTGGGGCCAGATGGTAAGCCCTCCCGTATCGTAGTTATCTACACGACGG
GGAGTCAGGCAACTATGGATGAACGAAATAGACAGATCGCTGAGATAGGTGCCTCACTGATT
AAGCATTGGTAGGAATTAATGATGTCTCGTTTAGATAAAAGTAAAGTGATTAACAGCGCATT

AGAGCTGCTTAATGAGGTCGGAATCGAAGGTTTAACAACCCGTAAACTCGCCCAGAAGCTAG
GTGTAGAGCAGCCTACATTGTATTGGCATGTAAAAAATAAGCGGGCTTTGCTCGACGCCTTA
GCCATTGAGATGTTAGATAGGCACCATACTCACTTTTGCCCTTTAGAAGGGGAAAG

>pASK-IBA5plus_6xHis-P91-WT
CTGGCAAGATTTTTTACGTAATAACGCTAAAAGTTTTAGATGTGCTTTACTAAGTCATCGCG
ATGGAGCAAAAGTACATTTAGGTACACGGCCTACAGAAAAACAGTATGAAACTCTCGAAAAT
CAATTAGCCTTTTTATGCCAACAAGGTTTTTCACTAGAGAATGCATTATATGCACTCAGCGC
AGTGGGGCATTTTACTTTAGGTTGCGTATTGGAAGATCAAGAGCATCAAGTCGCTAAAGAAG
AAAGGGAAACACCTACTACTGATAGTATGCCGCCATTATTACGACAAGCTATCGAATTATTT
GATCACCAAGGTGCAGAGCCAGCCTTCTTATTCGGCCTTGAATTGATCATATGCGGATTAGA
AAAACAACTTAAATGTGAAAGTGGGTCTTAAAAGCAGCATAACCTTTTTCCGTGATGGTAAC
TTCACTAGTTTAAAAGGATCTAGGTGAAGATCCTTTTTGATAATCTCATGACCAAAATCCCT
TAACGTGAGTTTTCGTTCCACTGAGCGTCAGACCCCGTAGAAAAGATCAAAGGATCTTCTTG
AGATCCTTTTTTTCTGCGCGTAATCTGCTGCTTGCAAACAAAAAAACCACCGCTACCAGCGG
TGGTTTGTTTGCCGGATCAAGAGCTACCAACTCTTTTTCCGAAGGTAACTGGCTTCAGCAGA
GCGCAGATACCAAATACTGTCCTTCTAGTGTAGCCGTAGTTAGGCCACCACTTCAAGAACTC
TGTAGCACCGCCTACATACCTCGCTCTGCTAATCCTGTTACCAGTGGCTGCTGCCAGTGGCG
ATAAGTCGTGTCTTACCGGGTTGGACTCAAGACGATAGTTACCGGATAAGGCGCAGCGGTCG
GGCTGAACGGGGGGTTCGTGCACACAGCCCAGCTTGGAGCGAACGACCTACACCGAACTGAG
ATACCTACAGCGTGAGCTATGAGAAAGCGCCACGCTTCCCGAAGGGAGAAAGGCGGACAGGT
ATCCGGTAAGCGGCAGGGTCGGAACAGGAGAGCGCACGAGGGAGCTTCCAGGGGGAAACGCC
TGGTATCTTTATAGTCCTGTCGGGTTTCGCCACCTCTGACTTGAGCGTCGATTTTTGTGATG
CTCGTCAGGGGGGCGGAGCCTATGGAAAAACGCCAGCAACGCGGCCTTTTTACGGTTCCTGG
CCTTTTGCTGGCCTTTTGCTCACATGACCCGACACCATCGAATGGCCAGATGATTAATTCCT
AATTTTTGTTGACACTCTATCATTGATAGAGTTATTTTACCACTCCCTATCAGTGATAGAGA
AAAGTGAAATGAATAGTTCGACAAAAATCTAGAAATAATTTTGTTTAACTTTAAGAAGGAGA
TATACAAATGCATCACCATCATCACCACGGTGGAAGTATGACAGCAAGAAAAGTCGACTACA
CAGACGGTGCAACCCGCTGTATCGGTGAGTTTCATTGGGATGAAGGCAAGTCGGGCCCGCGT
CCCGGCGTGGTGGTCTTTCCCGAGGCTTTCGGCCTCAACGACCATGCCAAGGAGCGCGCGCG
GCGCCTTGCCGACCTCGGCTTTGCAGCCCTGGCGGCGGATATGCACGGAGACGCCCAGGTTT
TCGATGCGGCGAGTCTCTCATCAACCATACAGGGCTACTACGGCGACCGCGCCCACTGGCGA
CGTCGTGCGCAGGCAGCGCTCGATGCACTGACGGCACAGCCAGAGGTGGACGGCAGCAAGGT
GGCGGCCATCGGCTTTTGTTTCGGCGGTGCCACCTGCCTTGAACTGGCCCGCACAGGTGCGC
CGCTGACCGCCATTGTCACCTTCCACGGCGGTTTGCTGCCGGAGATGGCAGGCGATGCCGGA
CGGATCCAGTCCAGTGTTCTGGTGTGCCATGGCGCTGATGATCCGCTCGTACAGGACGAAAC
CATGAAGGCCGTCATGGACGAGTTTCGTCGCGACAAGGTGGATTGGCAGGTGCTCTACCTCG
GAAATGCGGTACACAGTTTCACCGATCCACTCGCTGGCAGTCACGGCATACCCGGGCTGGCC
TATGACGCCACTGCCGAAGCCCGGTCGTGGACGGCCATGTGCAATCTGTTCAGTGAACTGTT
CGGCTGATGATATCTAACTAAGCTTGACCTGTGAAGTGAAAAATGGCGCACATTGTGCGACA
TTTTTTTTGTCTGCCGTTTACCGCTACTGCGTCACGGATCTCCACGCGCCCTGTAGCGGCGC
ATTAAGCGCGGCGGGTGTGGTGGTTACGCGCAGCGTGACCGCTACACTTGCCAGCGCCCTAG
CGCCCGCTCCTTTCGCTTTCTTCCCTTCCTTTCTCGCCACGTTCGCCGGCTTTCCCCGTCAA
GCTCTAAATCGGGGGCTCCCTTTAGGGTTCCGATTTAGTGCTTTACGGCACCTCGACCCCAA
AAAACTTGATTAGGGTGATGGTTCACGTAGTGGGCCATCGCCCTGATAGACGGTTTTTCGCC
CTTTGACGTTGGAGTCCACGTTCTTTAATAGTGGACTCTTGTTCCAAACTGGAACAACACTC
AACCCTATCTCGGTCTATTCTTTTGATTTATAAGGGATTTTGCCGATTTCGGCCTATTGGTT
AAAAAATGAGCTGATTTAACAAAAATTTAACGCGAATTTTAACAAAATATTAACGCTTACAA
TTTCAGGTGGCACTTTTCGGGGAAATGTGCGCGGAACCCCTATTTGTTTATTTTTCTAAATA
CATTCAAATATGTATCCGCTCATGAGACAATAACCCTGATAAATGCTTCAATAATATTGAAA
AAGGAAGAGTATGAGTATTCAACATTTCCGTGTCGCCCTTATTCCCTTTTTTGCGGCATTTT
GCCTTCCTGTTTTTGCTCACCCAGAAACGCTGGTGAAAGTAAAAGATGCTGAAGATCAGTTG

GGTGCACGAGTGGGTTACATCGAACTGGATCTCAACAGCGGTAAGATCCTTGAGAGTTTTCG
CCCCGAAGAACGTTTTCCAATGATGAGCACTTTTAAAGTTCTGCTATGTGGCGCGGTATTAT
CCCGTATTGACGCCGGGCAAGAGCAACTCGGTCGCCGCATACACTATTCTCAGAATGACTTG
GTTGAGTACTCACCAGTCACAGAAAAGCATCTTACGGATGGCATGACAGTAAGAGAATTATG
CAGTGCTGCCATAACCATGAGTGATAACACTGCGGCCAACTTACTTCTGACAACGATCGGAG
GACCGAAGGAGCTAACCGCTTTTTTGCACAACATGGGGGATCATGTAACTCGCCTTGATCGT
TGGGAACCGGAGCTGAATGAAGCCATACCAAACGACGAGCGTGACACCACGATGCCTGTAGC
AATGGCAACAACGTTGCGCAAACTATTAACTGGCGAACTACTTACTCTAGCTTCCCGGCAAC
AATTGATAGACTGGATGGAGGCGGATAAAGTTGCAGGACCACTTCTGCGCTCGGCCCTTCCG
GCTGGCTGGTTTATTGCTGATAAATCTGGAGCCGGTGAGCGTGGCTCTCGCGGTATCATTGC
AGCACTGGGGCCAGATGGTAAGCCCTCCCGTATCGTAGTTATCTACACGACGGGGAGTCAGG
CAACTATGGATGAACGAAATAGACAGATCGCTGAGATAGGTGCCTCACTGATTAAGCATTGG
TAGGAATTAATGATGTCTCGTTTAGATAAAAGTAAAGTGATTAACAGCGCATTAGAGCTGCT
TAATGAGGTCGGAATCGAAGGTTTAACAACCCGTAAACTCGCCCAGAAGCTAGGTGTAGAGC
AGCCTACATTGTATTGGCATGTAAAAAATAAGCGGGCTTTGCTCGACGCCTTAGCCATTGAG
ATGTTAGATAGGCACCATACTCACTTTTGCCCTTTAGAAGGGGAAAG

> pASK-IBA5plus_6xHis-P91-R1
CTGGCAAGATTTTTTACGTAATAACGCTAAAAGTTTTAGATGTGCTTTACTAAGTCATCGCG
ATGGAGCAAAAGTACATTTAGGTACACGGCCTACAGAAAAACAGTATGAAACTCTCGAAAAT
CAATTAGCCTTTTTATGCCAACAAGGTTTTTCACTAGAGAATGCATTATATGCACTCAGCGC
AGTGGGGCATTTTACTTTAGGTTGCGTATTGGAAGATCAAGAGCATCAAGTCGCTAAAGAAG
AAAGGGAAACACCTACTACTGATAGTATGCCGCCATTATTACGACAAGCTATCGAATTATTT
GATCACCAAGGTGCAGAGCCAGCCTTCTTATTCGGCCTTGAATTGATCATATGCGGATTAGA
AAAACAACTTAAATGTGAAAGTGGGTCTTAAAAGCAGCATAACCTTTTTCCGTGATGGTAAC
TTCACTAGTTTAAAAGGATCTAGGTGAAGATCCTTTTTGATAATCTCATGACCAAAATCCCT
TAACGTGAGTTTTCGTTCCACTGAGCGTCAGACCCCGTAGAAAAGATCAAAGGATCTTCTTG
AGATCCTTTTTTTCTGCGCGTAATCTGCTGCTTGCAAACAAAAAAACCACCGCTACCAGCGG
TGGTTTGTTTGCCGGATCAAGAGCTACCAACTCTTTTTCCGAAGGTAACTGGCTTCAGCAGA
GCGCAGATACCAAATACTGTCCTTCTAGTGTAGCCGTAGTTAGGCCACCACTTCAAGAACTC
TGTAGCACCGCCTACATACCTCGCTCTGCTAATCCTGTTACCAGTGGCTGCTGCCAGTGGCG
ATAAGTCGTGTCTTACCGGGTTGGACTCAAGACGATAGTTACCGGATAAGGCGCAGCGGTCG
GGCTGAACGGGGGGTTCGTGCACACAGCCCAGCTTGGAGCGAACGACCTACACCGAACTGAG
ATACCTACAGCGTGAGCTATGAGAAAGCGCCACGCTTCCCGAAGGGAGAAAGGCGGACAGGT
ATCCGGTAAGCGGCAGGGTCGGAACAGGAGAGCGCACGAGGGAGCTTCCAGGGGGAAACGCC
TGGTATCTTTATAGTCCTGTCGGGTTTCGCCACCTCTGACTTGAGCGTCGATTTTTGTGATG
CTCGTCAGGGGGGCGGAGCCTATGGAAAAACGCCAGCAACGCGGCCTTTTTACGGTTCCTGG
CCTTTTGCTGGCCTTTTGCTCACATGACCCGACACCATCGAATGGCCAGATGATTAATTCCT
AATTTTTGTTGACACTCTATCATTGATAGAGTTATTTTACCACTCCCTATCAGTGATAGAGA
AAAGTGAAATGAATAGTTCGACAAAAATCTAGAAATAATTTTGTTTAACTTTAAGAAGGAGA
TATACAAATGCATCACCATCATCACCACGGTGGAAGTATGACAGCAAGAAAAGTCGACTACA
CAGACGGTGCAACCCGCTGTATCGGTGAGTTTCATTGGGATGAAGGCAAGTCGGGCCCGCGT
CCCGGCGTGGTGGTCTTTCCCGAGGCTTTCGGCCTCAACGACCATGCCAAGGAGCGCGCGCG
GCGCCTTGCCGACCTCGGCTTTGCAGCCCTGGCGGCGGATATGCACGGAGACGCCCAGGTTT
TCGATGCGGCGAGTCTCTCATCAACCATACAGGGCTACTACGGCGACCGCGCCCACTGGCGA
CGTCGTGCGCAGGCAGCGCTCGATGCACTGACGGCACAGCCAGAGGTGGACGGCAGCAAGGT
GGCGGCCATCGGCTTTTGTTTCGGCGGTGCCACCTGCCTTGAACTGGCCCGCACAGGTGCGC
CGCTGACCGCCATTGTCACCTTCCACGGCGGTTTGCTGCCGGAGATGGCAGGCGATGCCGGA
CGGATCCAGTCCAGTGTTCTGGTGTGCCATGGCGCTGATGATCCGCTCGTACAGGACGAAAC
CATGAAGGCCGTCATGGACGAGTTTCGTCGCGACAAGGTGGATTGGCAGGTGCTCTACCTCG
GAAATGCGGTACACAGTTTCACCGATCCACTCGCTGGCAGTCACGGC**TGG**CCCGGG**GTT**GCC

TATGACGCCACTGCCGAAGCCCGGTCGTGGACGGCCATGTGCAATCTGTTCAGTGAACTGTT
CGGCTGATGATATCTAACTAAGCTTGACCTGTGAAGTGAAAAATGGCGCACATTGTGCGACA
TTTTTTTTGTCTGCCGTTTACCGCTACTGCGTCACGGATCTCCACGCGCCCTGTAGCGGCGC
ATTAAGCGCGGCGGGTGTGGTGGTTACGCGCAGCGTGACCGCTACACTTGCCAGCGCCCTAG
CGCCCGCTCCTTTCGCTTTCTTCCCTTCCTTTCTCGCCACGTTCGCCGGCTTTCCCCGTCAA
GCTCTAAATCGGGGGCTCCCTTTAGGGTTCCGATTTAGTGCTTTACGGCACCTCGACCCCAA
AAAACTTGATTAGGGTGATGGTTCACGTAGTGGGCCATCGCCCTGATAGACGGTTTTTCGCC
CTTTGACGTTGGAGTCCACGTTCTTTAATAGTGGACTCTTGTTCCAAACTGGAACAACACTC
AACCCTATCTCGGTCTATTCTTTTGATTTATAAGGGATTTTGCCGATTTCGGCCTATTGGTT
AAAAAATGAGCTGATTTAACAAAAATTTAACGCGAATTTTAACAAAATATTAACGCTTACAA
TTTCAGGTGGCACTTTTCGGGGAAATGTGCGCGGAACCCCTATTTGTTTATTTTTCTAAATA
CATTCAAATATGTATCCGCTCATGAGACAATAACCCTGATAAATGCTTCAATAATATTGAAA
AAGGAAGAGTATGAGTATTCAACATTTCCGTGTCGCCCTTATTCCCTTTTTTGCGGCATTTT
GCCTTCCTGTTTTTGCTCACCCAGAAACGCTGGTGAAAGTAAAAGATGCTGAAGATCAGTTG
GGTGCACGAGTGGGTTACATCGAACTGGATCTCAACAGCGGTAAGATCCTTGAGAGTTTTCG
CCCCGAAGAACGTTTTCCAATGATGAGCACTTTTAAAGTTCTGCTATGTGGCGCGGTATTAT
CCCGTATTGACGCCGGGCAAGAGCAACTCGGTCGCCGCATACACTATTCTCAGAATGACTTG
GTTGAGTACTCACCAGTCACAGAAAAGCATCTTACGGATGGCATGACAGTAAGAGAATTATG
CAGTGCTGCCATAACCATGAGTGATAACACTGCGGCCAACTTACTTCTGACAACGATCGGAG
GACCGAAGGAGCTAACCGCTTTTTTGCACAACATGGGGGATCATGTAACTCGCCTTGATCGT
TGGGAACCGGAGCTGAATGAAGCCATACCAAACGACGAGCGTGACACCACGATGCCTGTAGC
AATGGCAACAACGTTGCGCAAACTATTAACTGGCGAACTACTTACTCTAGCTTCCCGGCAAC
AATTGATAGACTGGATGGAGGCGGATAAAGTTGCAGGACCACTTCTGCGCTCGGCCCTTCCG
GCTGGCTGGTTTATTGCTGATAAATCTGGAGCCGGTGAGCGTGGCTCTCGCGGTATCATTGC
AGCACTGGGGCCAGATGGTAAGCCCTCCCGTATCGTAGTTATCTACACGACGGGGAGTCAGG
CAACTATGGATGAACGAAATAGACAGATCGCTGAGATAGGTGCCTCACTGATTAAGCATTGG
TAGGAATTAATGATGTCTCGTTTAGATAAAAGTAAAGTGATTAACAGCGCATTAGAGCTGCT
TAATGAGGTCGGAATCGAAGGTTTAACAACCCGTAAACTCGCCCAGAAGCTAGGTGTAGAGC
AGCCTACATTGTATTGGCATGTAAAAAATAAGCGGGCTTTGCTCGACGCCTTAGCCATTGAG
ATGTTAGATAGGCACCATACTCACTTTTGCCCTTTAGAAGGGGAAAG

> pASK-IBA5plus_6xHis-P91-R2
CTGGCAAGATTTTTTACGTAATAACGCTAAAAGTTTTAGATGTGCTTTACTAAGTCATCGCG
ATGGAGCAAAAGTACATTTAGGTACACGGCCTACAGAAAAACAGTATGAAACTCTCGAAAAT
CAATTAGCCTTTTTATGCCAACAAGGTTTTTCACTAGAGAATGCATTATATGCACTCAGCGC
AGTGGGGCATTTTACTTTAGGTTGCGTATTGGAAGATCAAGAGCATCAAGTCGCTAAAGAAG
AAAGGGAAACACCTACTACTGATAGTATGCCGCCATTATTACGACAAGCTATCGAATTATTT
GATCACCAAGGTGCAGAGCCAGCCTTCTTATTCGGCCTTGAATTGATCATATGCGGATTAGA
AAAACAACTTAAATGTGAAAGTGGGTCTTAAAAGCAGCATAACCTTTTTCCGTGATGGTAAC
TTCACTAGTTTAAAAGGATCTAGGTGAAGATCCTTTTTGATAATCTCATGACCAAAATCCCT
TAACGTGAGTTTTCGTTCCACTGAGCGTCAGACCCCGTAGAAAAGATCAAAGGATCTTCTTG
AGATCCTTTTTTTCTGCGCGTAATCTGCTGCTTGCAAACAAAAAAACCACCGCTACCAGCGG
TGGTTTGTTTGCCGGATCAAGAGCTACCAACTCTTTTTCCGAAGGTAACTGGCTTCAGCAGA
GCGCAGATACCAAATACTGTCCTTCTAGTGTAGCCGTAGTTAGGCCACCACTTCAAGAACTC
TGTAGCACCGCCTACATACCTCGCTCTGCTAATCCTGTTACCAGTGGCTGCTGCCAGTGGCG
ATAAGTCGTGTCTTACCGGGTTGGACTCAAGACGATAGTTACCGGATAAGGCGCAGCGGTCG
GGCTGAACGGGGGGTTCGTGCACACAGCCCAGCTTGGAGCGAACGACCTACACCGAACTGAG
ATACCTACAGCGTGAGCTATGAGAAAGCGCCACGCTTCCCGAAGGGAGAAAGGCGGACAGGT
ATCCGGTAAGCGGCAGGGTCGGAACAGGAGAGCGCACGAGGGAGCTTCCAGGGGGAAACGCC
TGGTATCTTTATAGTCCTGTCGGGTTTCGCCACCTCTGACTTGAGCGTCGATTTTTGTGATG
CTCGTCAGGGGGGCGGAGCCTATGGAAAAACGCCAGCAACGCGGCCTTTTTACGGTTCCTGG
CCTTTTGCTGGCCTTTTGCTCACATGACCCGACACCATCGAATGGCCAGATGATTAATTCCT

AATTTTTGTTGACACTCTATCATTGATAGAGTTATTTTACCACTCCCTATCAGTGATAGAGA
AAAGTGAAATGAATAGTTCGACAAAAATCTAGAAATAATTTTGTTTAACTTTAAGAAGGAGA
TATACAAATGCATCACCATCATCACCACGGTGGAAGTATGACAGCAAGAAAAGTCGACTACA
CAGACGGTGCAACCCGCTGTATCGGTGAGTTTCATTGGGATGAAGGCAAGTCGGGCCCGCGT
CCCGGCGTGGTGGTCTTTCCCGAG**CTG**TTCGGCCTCAACGACCATGCCAAGGAGCGCGCGCG
GCGCCTTGCCGACCTCGGCTTTGCAGCCCTGGCGGCGGATATGCACGGAGACGCCCAGGTTT
TCGAT**GAG**GCGAGT**GTG**TCATCAACCATACAGGGCTACTACGGCGACCGCGCCCACTGGCGA
CGTCGTGCGCAGGCAGCGCTCGATGCACTGACGGCACAGCCAGAGGTGGACGGCAGCAAGGT
GGCGGCCATCGGCTTTTGTTTCGGCGGTGCGACCTGCCTTGAACTGGCCCGCACAGGTGCGC
CGCTGACCGCCATTGTCACCTTCCACGGCGGTTTGCTGCCGGAGATGGCAGGCGATGCCGGA
CGGATCCAGTCCAGTGTTCTGGTGTGCCATGGCGCTGATGATCCGCTCGTACAGGACGAAAC
CATGAAGGCCGTCATGGACGAGTTTCGTCGCGACAAGGTGGATTGGCAGGTGCTCTACCTCG
GAAATGCGGTACACAGTTTCACCGATCCACTCGCTGGCAGTCACGGC**TGG**CCCGGG**GTT**GCC
TATGACGCCACTGCCGAAGCCCGGTCGTGGACGGCCATGTGCAATCTGTTCAGTGAACTGTT
CGGCTGATGATATCTAACTAAGCTTGACCTGTGAAGTGAAAAATGGCGCACATTGTGCGACA
TTTTTTTTGTCTGCCGTTTACCGCTACTGCGTCACGGATCTCCACGCGCCCTGTAGCGGCGC
ATTAAGCGCGGCGGGTGTGGTGGTTACGCGCAGCGTGACCGCTACACTTGCCAGCGCCCTAG
CGCCCGCTCCTTTCGCTTTCTTCCCTTCCTTTCTCGCCACGTTCGCCGGCTTTCCCCGTCAA
GCTCTAAATCGGGGGCTCCCTTTAGGGTTCCGATTTAGTGCTTTACGGCACCTCGACCCCAA
AAAACTTGATTAGGGTGATGGTTCACGTAGTGGGCCATCGCCCTGATAGACGGTTTTTCGCC
CTTTGACGTTGGAGTCCACGTTCTTTAATAGTGGACTCTTGTTCCAAACTGGAACAACACTC
AACCCTATCTCGGTCTATTCTTTTGATTTATAAGGGATTTTGCCGATTTCGGCCTATTGGTT
AAAAAATGAGCTGATTTAACAAAAATTTAACGCGAATTTTAACAAAATATTAACGCTTACAA
TTTCAGGTGGCACTTTTCGGGGAAATGTGCGCGGAACCCCTATTTGTTTATTTTTCTAAATA
CATTCAAATATGTATCCGCTCATGAGACAATAACCCTGATAAATGCTTCAATAATATTGAAA
AAGGAAGAGTATGAGTATTCAACATTTCCGTGTCGCCCTTATTCCCTTTTTTGCGGCATTTT
GCCTTCCTGTTTTTGCTCACCCAGAAACGCTGGTGAAAGTAAAAGATGCTGAAGATCAGTTG
GGTGCACGAGTGGGTTACATCGAACTGGATCTCAACAGCGGTAAGATCCTTGAGAGTTTTCG
CCCCGAAGAACGTTTTCCAATGATGAGCACTTTTAAAGTTCTGCTATGTGGCGCGGTATTAT
CCCGTATTGACGCCGGGCAAGAGCAACTCGGTCGCCGCATACACTATTCTCAGAATGACTTG
GTTGAGTACTCACCAGTCACAGAAAAGCATCTTACGGATGGCATGACAGTAAGAGAATTATG
CAGTGCTGCCATAACCATGAGTGATAACACTGCGGCCAACTTACTTCTGACAACGATCGGAG
GACCGAAGGAGCTAACCGCTTTTTTGCACAACATGGGGGATCATGTAACTCGCCTTGATCGT
TGGGAACCGGAGCTGAATGAAGCCATACCAAACGACGAGCGTGACACCACGATGCCTGTAGC
AATGGCAACAACGTTGCGCAAACTATTAACTGGCGAACTACTTACTCTAGCTTCCCGGCAAC
AATTGATAGACTGGATGGAGGCGGATAAAGTTGCAGGACCACTTCTGCGCTCGGCCCTTCCG
GCTGGCTGGTTTATTGCTGATAAATCTGGAGCCGGTGAGCGTGGCTCTCGCGGTATCATTGC
AGCACTGGGGCCAGATGGTAAGCCCTCCCGTATCGTAGTTATCTACACGACGGGGAGTCAGG
CAACTATGGATGAACGAAATAGACAGATCGCTGAGATAGGTGCCTCACTGATTAAGCATTGG
TAGGAATTAATGATGTCTCGTTTAGATAAAAGTAAAGTGATTAACAGCGCATTAGAGCTGCT
TAATGAGGTCGGAATCGAAGGTTTAACAACCCGTAAACTCGCCCAGAAGCTAGGTGTAGAGC
AGCCTACATTGTATTGGCATGTAAAAAATAAGCGGGCTTTGCTCGACGCCTTAGCCATTGAG
ATGTTAGATAGGCACCATACTCACTTTTGCCCTTTAGAAGGGGAAAG

4.2 Primer sequences

Annealing parts are shown in uppercase, overhangs in lowercase. Mutagenic or degenerate codons are highlighted in bold.

Sequencing primers for pASK-IBA5+ constructs:

Label	Sequence (5'→3')
IF	GAGTTATTTTACCACTCCCT
IR	CGCAGTAGCGGTAAACG

Saturation mutagenesis for mutational scanning:

Label	Sequence (5'→3')
P91 SM 37 NDT fwd	gattacaggtctcgTCCC**NDT**GCTTTCGGCCTCAACG
P91 SM 37 VHG fwd	gattacaggtctcgTCCC**VHG**GCTTTCGGCCTCAACG
P91 SM 37 TGG fwd	gattacaggtctcgTCCC**TGG**GCTTTCGGCCTCAACG
P91 SM 37 rev	gattacaggtctcgGGGAAAGACCACCACGC
P91 SM 38 NDT fwd	gattacaggtctcgCGAG**NDT**TTCGGCCTCAACGACC
P91 SM 38 VHG fwd	gattacaggtctcgCGAG**VHG**TTCGGCCTCAACGACC
P91 SM 38 TGG fwd	gattacaggtctcgCGAG**TGG**TTCGGCCTCAACGACC
P91 SM 38 rev	gattacaggtctcgCTCGGGAAAGACCACCAC
P91 SM 39 NDT fwd	gattacaggtctcgGGCT**NDT**GGCCTCAACGACCATGC
P91 SM 39 VHG fwd	gattacaggtctcgGGCT**VHG**GGCCTCAACGACCATGC
P91 SM 39 TGG fwd	gattacaggtctcgGGCT**TGG**GGCCTCAACGACCATGC
P91 SM 39 rev	gattacaggtctcgAGCCTCGGGAAAGACCAC
P91 SM 71 NDT fwd	ggtctcgGGTT**NDT**GATGCGGCGAGTCTCTC
P91 SM 71 VHG fwd	ggtctcgGGTT**VHG**GATGCGGCGAGTCTCTC
P91 SM 71 TGG fwd	ggtctcgGGTT**TGG**GATGCGGCGAGTCTCTC
P91 SM 71 rev	ggtctcgAACCTGGGCGTCTCCG
P91 SM 72 NDT fwd	ggtctcgTTTC**NDT**GCGGCGAGTCTCTCATC
P91 SM 72 VHG fwd	ggtctcgTTTC**VHG**GCGGCGAGTCTCTCATC
P91 SM 72 TGG fwd	ggtctcgTTTC**TGG**GCGGCGAGTCTCTCATC
P91 SM 72 rev	ggtctcgGAAAACCTGGGCGTCTCC
P91 SM 73 NDT fwd	ggtctcgCGAT**NDT**GCGAGTCTCTCATCAACCATAC
P91 SM 73 VHG fwd	ggtctcgCGAT**VHG**GCGAGTCTCTCATCAACCATAC
P91 SM 73 TGG fwd	ggtctcgCGAT**TGG**GCGAGTCTCTCATCAACCATAC
P91 SM 71-74 rev	ggtctcgATCGAAAACCTGGGCG
P91 SM 74 NDT fwd	ggtctcgTGCG**NDT**AGTCTCTCATCAACCATACAGGG
P91 SM 74 VHG fwd	ggtctcgTGCG**VHG**AGTCTCTCATCAACCATACAGGG
P91 SM 74 TGG fwd	ggtctcgTGCG**TGG**AGTCTCTCATCAACCATACAGGG
P91 SM 71-74 rev	ggtctcgCGCATCGAAAACCTGG
P91 SM 76 NDT fwd	gattacaggtctcgGAGT**NDT**TCATCAACCATACAGGGCTACTAC
P91 SM 76 VHG fwd	gattacaggtctcgGAGT**VHG**TCATCAACCATACAGGGCTACTAC
P91 SM 76 TGG fwd	gattacaggtctcgGAGT**TGG**TCATCAACCATACAGGGCTACTAC
P91 SM 76 rev	gattacaggtctccACTCGCCGCATCGAA
P91 SM 77 NDT fwd	gattacaggtctctTCTC**NDT**TCAACCATACAGGGCTACTACG
P91 SM 77 VHG fwd	gattacaggtctctTCTC**VHG**TCAACCATACAGGGCTACTACG
P91 SM 77 TGG fwd	gattacaggtctctTCTC**TGG**TCAACCATACAGGGCTACTACG
P91 SM 77 rev	gattacaggtctcgGAGACTCGCCGCATCG
P91 SM 80 NDT fwd	ggtctcgAACC**NDT**CAGGGCTACTACGGCGAC
P91 SM 80 VHG fwd	ggtctcgAACC**VHG**CAGGGCTACTACGGCGAC
P91 SM 80 TGG fwd	ggtctcgAACC**TGG**CAGGGCTACTACGGCGAC
P91 SM 80 rev	ggtctcgGGTTGATGAGAGACTCGCC
P91 SM 81 NDT fwd	gattacaggtctcgCATA**NDT**GGCTACTACGGCGACCG
P91 SM 81 VHG fwd	gattacaggtctcgCATA**VHG**GGCTACTACGGCGACCG
P91 SM 81 TGG fwd	gattacaggtctcgCATA**TGG**GGCTACTACGGCGACCG
P91 SM 81 rev	gattacaggtctccTATGGTTGATGAGAGACTCGCC
P91 SM 84 NDT fwd	gattacaggtctcaCTAC**NDT**GGCGACCGCGC

P91 SM 84 VHG fwd	gattacaggtctcaCTAC**VHG**GGCGACCGCGC
P91 SM 84 TGG fwd	gattacaggtctcaCTAC**TGG**GGCGACCGCGC
P91 SM 84 rev	gattacaggtctcgGTAGCCCTGTATGGTTGATGAG
P91 SM 119 NDT fwd	gattacaggtctcgTTGT**NDT**GGCGGTGCCACCT
P91 SM 119 VHG fwd	gattacaggtctcgTTGT**VHG**GGCGGTGCCACCT
P91 SM 119 TGG fwd	gattacaggtctcgTTGT**TGG**GGCGGTGCCACCT
P91 SM 119 rev	gattacaggtctcgACAAAAGCCGATGGCC
P91 SM 122 NDT fwd	gattacaggtctcaCGGT**NDT**ACCTGCCTTGAACTGGC
P91 SM 122 VHG fwd	gattacaggtctcaCGGT**VHG**ACCTGCCTTGAACTGGC
P91 SM 122 TGG fwd	gattacaggtctcaCGGT**TGG**ACCTGCCTTGAACTGGC
P91 SM 122 rev	gattacaggtctcgACCGCCGAAACAAAAGC
P91 SM 141 NDT fwd	gattacaggtctcgCTTC**NDT**GGCGGTTTGCTGCC
P91 SM 141 VHG fwd	gattacaggtctcgCTTC**VHG**GGCGGTTTGCTGCC
P91 SM 141 TGG fwd	gattacaggtctcgCTTC**TGG**GGCGGTTTGCTGCC
P91 SM 141 rev	gattacaggtctcaGAAGGTGACAATGGCGG
P91 SM 143 NDT fwd	gattacaggtctcgCGGC**NDT**TTGCTGCCGGAGATGG
P91 SM 143 VHG fwd	gattacaggtctcgCGGC**VHG**TTGCTGCCGGAGATGG
P91 SM 143 TGG fwd	gattacaggtctcgCGGC**TGG**TTGCTGCCGGAGATGG
P91 SM 143 rev	gattacaggtctcgGCCGTGGAAGGTGACAAT
P91 SM 167 NDT fwd	gattacaggtctcgTGAT**NDT**CCGCTCGTACAGGACG
P91 SM 167 VHG fwd	gattacaggtctcgTGAT**VHG**CCGCTCGTACAGGACG
P91 SM 167 TGG fwd	gattacaggtctcgTGAT**TGG**CCGCTCGTACAGGACG
P91 SM 167 rev	gattacaggtctcgATCAGCGCCATGGCA
P91 SM 169 NDT fwd	gattacaggtctcgTCCG**NDT**GTACAGGACGAAACCATGAAGG
P91 SM 169 VHG fwd	gattacaggtctcgTCCG**VHG**GTACAGGACGAAACCATGAAGG
P91 SM 169 TGG fwd	gattacaggtctcgTCCG**TGG**GTACAGGACGAAACCATGAAGG
P91 SM 169 rev	gattacaggtctcaCGGATCATCAGCGCC
P91 SM 197 NDT fwd	GGTCTCCAAAT**NDT**GTACACAGTTTCACCGATCCAC
P91 SM 197 VHG fwd	GGTCTCCAAAT**VHG**GTACACAGTTTCACCGATCCAC
P91 SM 197 TGG fwd	GGTCTCCAAAT**TGG**GTACACAGTTTCACCGATCCAC
P91 SM 197 rev	ggtctcgATTTCCGAGGTAGAGCACCTG
P91 SM 199 NDT fwd	gattacaggtctcgGGTA**NDT**AGTTTCACCGATCCACTCG
P91 SM 199 VHG fwd	gattacaggtctcgGGTA**VHG**AGTTTCACCGATCCACTCG
P91 SM 199 TGG fwd	gattacaggtctcgGGTA**TGG**AGTTTCACCGATCCACTCG
P91 SM 199 rev	GATTACAGGTCTCCTACCGCATTTCCGAGGTAG
P91 SM 200 NDT fwd	gattacaggtctcgACAC**NDT**TTCACCGATCCACTCGC
P91 SM 200 VHG fwd	gattacaggtctcgACAC**VHG**TTCACCGATCCACTCGC
P91 SM 200 TGG fwd	gattacaggtctcgACAC**TGG**TTCACCGATCCACTCGC
P91 SM 200 rev	gattacaggtctcgGTGTACCGCATTTCCGAG
P91 SM 211 NDT fwd	gattacaggtctcgCGGC**NDT**CCCGGGCTGGCC
P91 SM 211 VHG fwd	gattacaggtctcgCGGC**VHG**CCCGGGCTGGCC
P91 SM 211 TGG fwd	gattacaggtctcgCGGC**TGG**CCCGGGCTGGCC
P91 SM 211 rev	gattacaggtctcgGCCGTGACTGCCAGC
P91 SM 214 NDT fwd	gattacaggtctcgCGGG**NDT**GCCTATGACGCCACTGC
P91 SM 214 VHG fwd	gattacaggtctcgCGGG**VHG**GCCTATGACGCCACTGC
P91 SM 214 TGG fwd	gattacaggtctcgCGGG**TGG**GCCTATGACGCCACTGC
P91 SM 214 rev	gattacaggtctcaCCCGGGTATGCCGT

Nucleophile exchange:

Label	Sequence (5'→3')
P91 WT/R1 C118S fwd	gattacaggtctccCTTT**AGC**TTCGGCGGTGCCAC
P91 R2 C118S fwd	gattacaggtctccCTTT**AGC**TTCGGCGGTGCG
P91 C118S rev	gattacaggtctcgAAAGCCGATGGCCG

Construction of library P91-A (round 1)

Fragment	Label	Sequence (5'→3')
1	P91-A Frag 1 A73NNK fwd	ACGCCCAGGTTTTCGAT**NNK**GCGAGTCTCTCATCAACCATAC

	P91-A Frag1 rev	ATCGAAAACCTGGGCGT
2	P91-A Frag2 211NNK 214NNK fwd	GCTGGCAGTCACGGC**NNK**CCCGGG**NNK**GCCTATGACGCCACTGC
	P91-A Frag2 rev	GCCGTGACTGCCAGC

Construction of library P91-B (round 2)

Fragment	Label	Sequence (5'→3')
1	P91 SM 38 NDT fwd	gattacaggtctcgCGAG**NDT**TTCGGCCTCAACGACC
	P91 SM 38 VHG fwd	gattacaggtctcgCGAG**VHG**TTCGGCCTCAACGACC
	P91 SM 71-74 rev	ggtctcgATCGAAAACCTGGGCG
	P91 SM 38 TGG fwd	gattacaggtctcgCGAG**TGG**TTCGGCCTCAACGACC
2	P91 A73NDT L76NDT fwd	gattacaggtctcgCGAT**NDT**GCGAGT**NDT**TCATCAACCATACAGGGCTACTAC
	P91 A73NDT L76VHG fwd	gattacaggtctcgCGAT**NDT**GCGAGT**VHG**TCATCAACCATACAGGGCTACTAC
	P91 A73NDT L76TGG fwd	gattacaggtctcgCGAT**NDT**GCGAGT**TGG**TCATCAACCATACAGGGCTACTAC
	P91 A73VHG L76NDT fwd	gattacaggtctcgCGAT**VHG**GCGAGT**NDT**TCATCAACCATACAGGGCTACTAC
	P91 A73VHG L76VHG fwd	gattacaggtctcgCGAT**VHG**GCGAGT**VHG**TCATCAACCATACAGGGCTACTAC
	P91 A73VHG L76TGG fwd	gattacaggtctcgCGAT**VHG**GCGAGT**TGG**TCATCAACCATACAGGGCTACTAC
	P91 A73TGG L76NDT fwd	gattacaggtctcgCGAT**TGG**GCGAGT**NDT**TCATCAACCATACAGGGCTACTAC
	P91 A73TGG L76VHG fwd	gattacaggtctcgCGAT**TGG**GCGAGT**VHG**TCATCAACCATACAGGGCTACTAC
	P91 A73TGG L76TGG fwd	gattacaggtctcgCGAT**TGG**GCGAGT**TGG**TCATCAACCATACAGGGCTACTAC
	P91 SM 122 rev	gattacaggtctcgACCGCCGAAACAAAAGC
3	P91 SM 122 NDT fwd	gattacaggtctcaCGGT**NDT**ACCTGCCTTGAACTGGC
	P91 SM 122 VHG fwd	gattacaggtctcaCGGT**VHG**ACCTGCCTTGAACTGGC
	P91 SM 122 TGG fwd	gattacaggtctcaCGGT**TGG**ACCTGCCTTGAACTGGC
	P91 SM 38 rev	gattacaggtctcgCTCGGAAAGACCACCAC

Introduction of an N-terminal 6xHis-tag:

Label	Sequence (5'→3')
pASK_strepless_rev	gattacaggtctcgTTGTATATCTCCTTCTTAAAGTTAAACAAAATTATTTCTAG
6xHis_P91_fwd	gattacaggtctcgACAA**atgcatcaccatcatcaccacggtggaagt**ATGACAGCAAGAAAAGTCGAC

5. NMR spectra

NMR data were collected at 298 K using Bruker Avance spectrometers with ^{1}H resonance frequencies of 400 MHz.

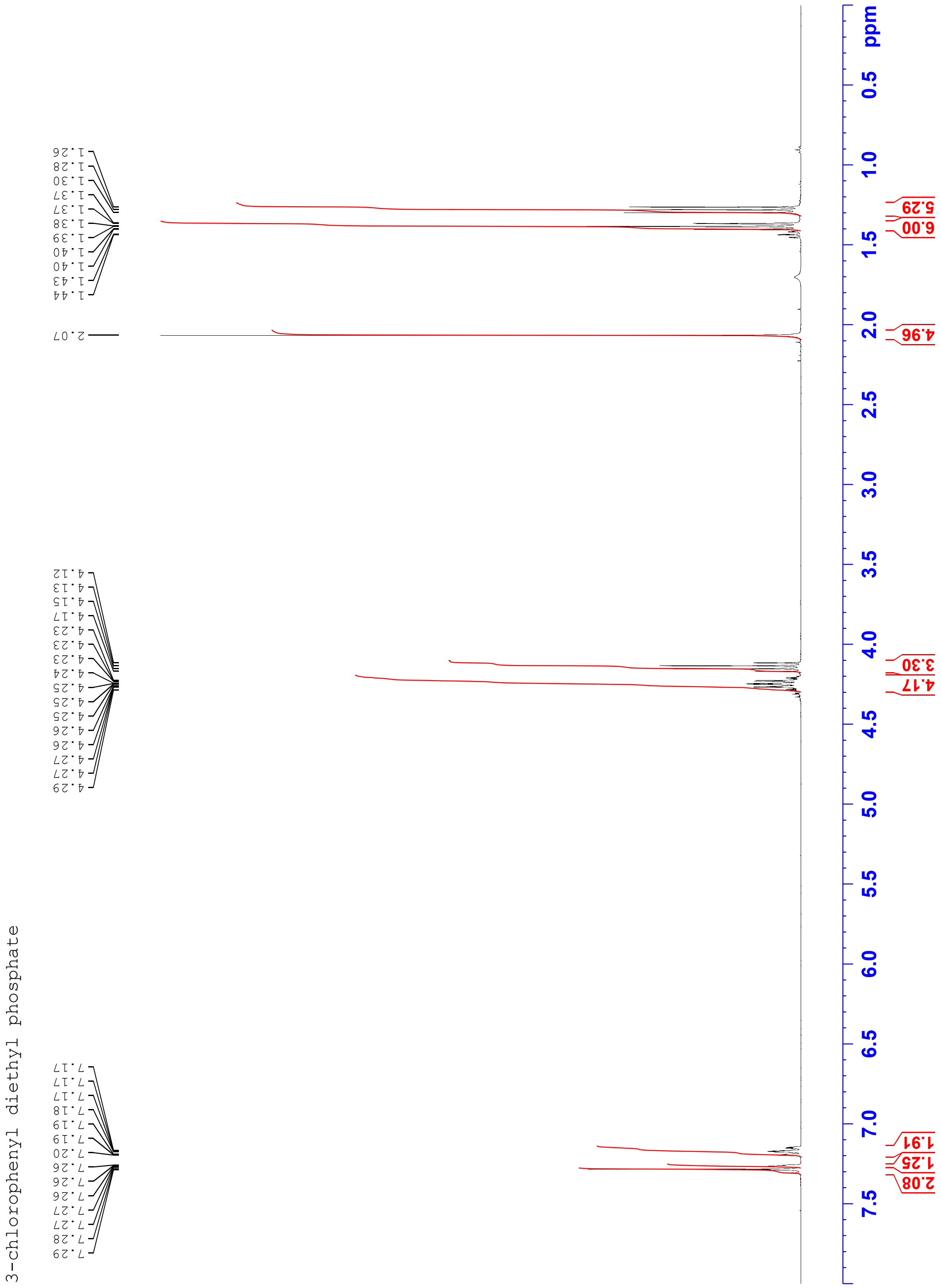

3-cyanophennyl diethyl phosphate

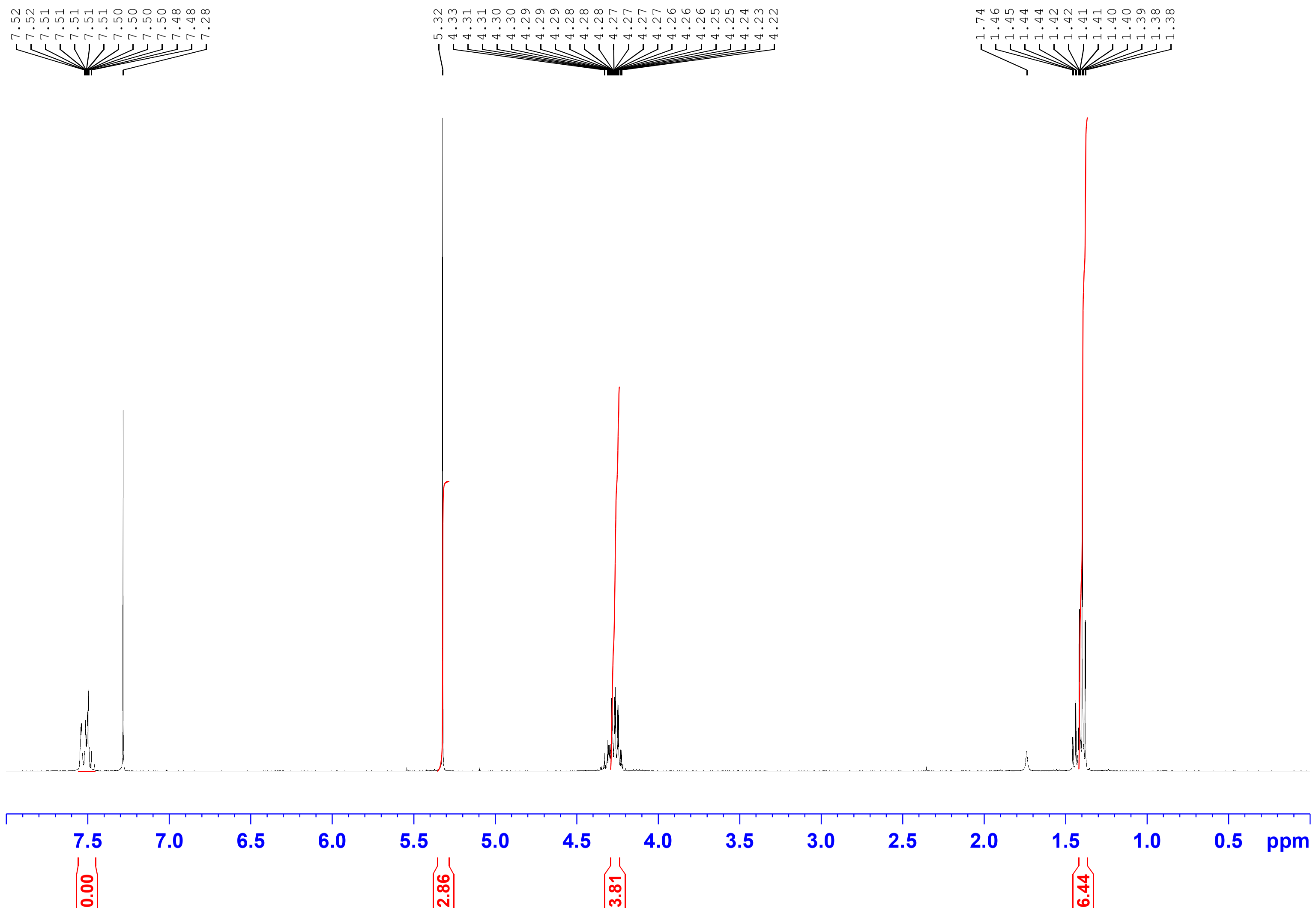

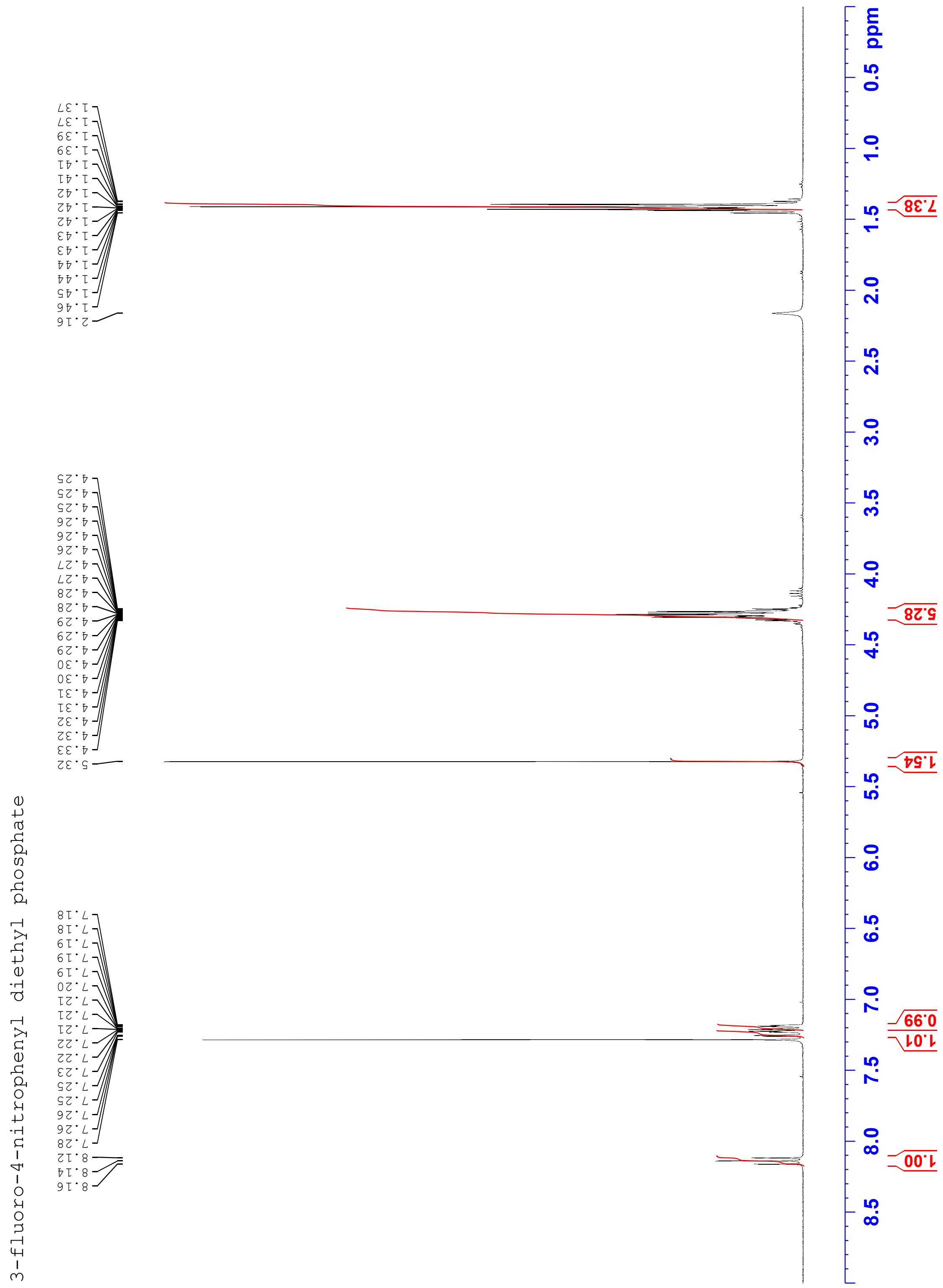
3-fluoro-4-nitrophenyl diethyl phosphate
ppm
7.38
5.28
1.54
0.99
1.01
1.00

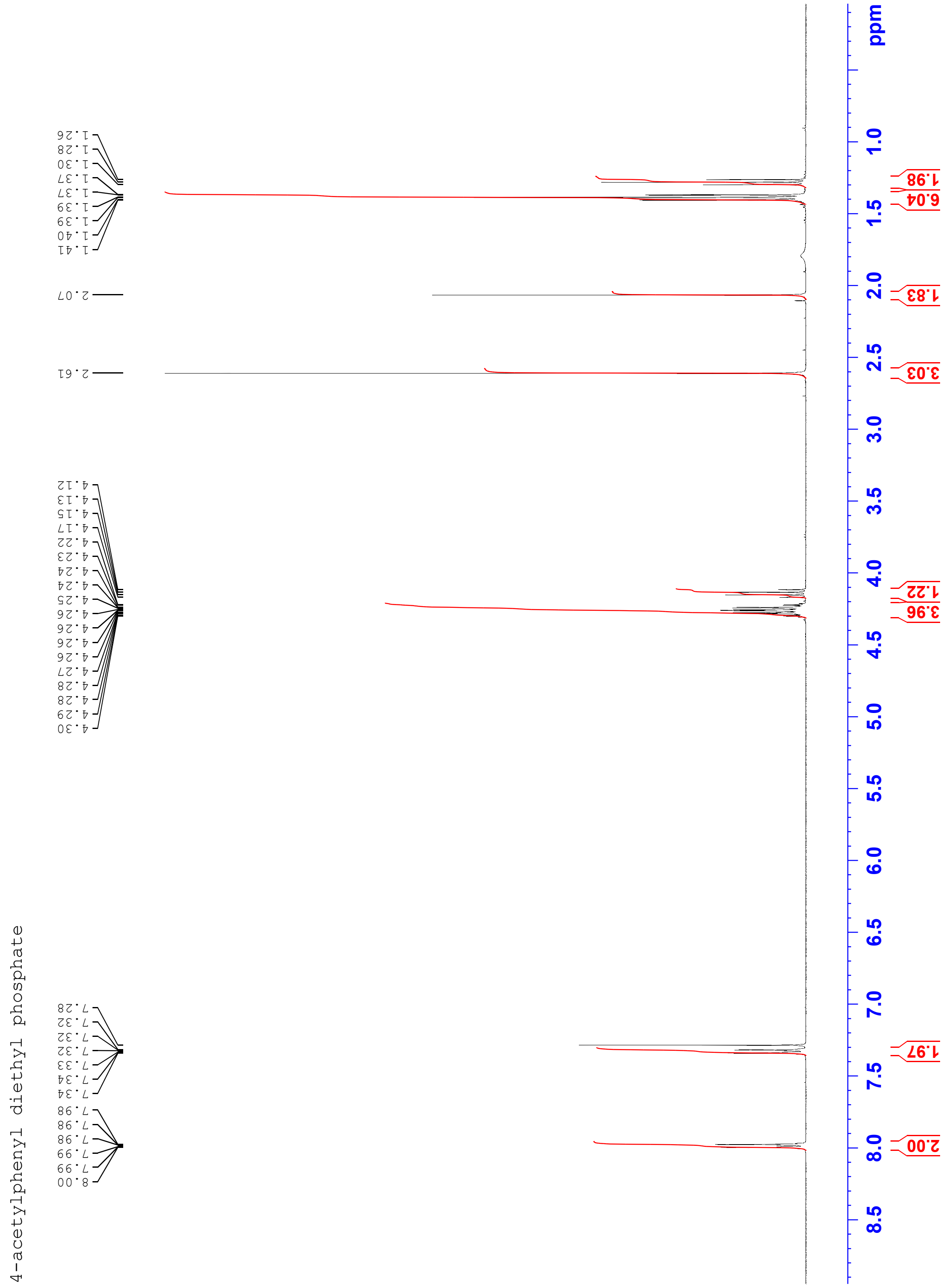
4-acetylphenyl diethyl phosphate
1.26
1.28
1.30
1.37
1.37
1.39
1.39
1.40
1.41
2.07
2.61
4.12
4.13
4.15
4.17
4.22
4.23
4.24
4.24
4.25
4.26
4.26
4.26
4.26
4.26
4.27
4.28
4.28
4.29
4.30
7.28
7.32
7.32
7.32
7.33
7.34
7.34
7.98
7.98
7.98
7.99
7.99
8.00
1.98
6.04
1.83
3.03
1.22
3.96
1.97
2.00
ppm
1.0
1.5
2.0
2.5
3.0
3.5
4.0
4.5
5.0
5.5
6.0
6.5
7.0
7.5
8.0
8.5

4-cyanophenyl diethyl phosphate

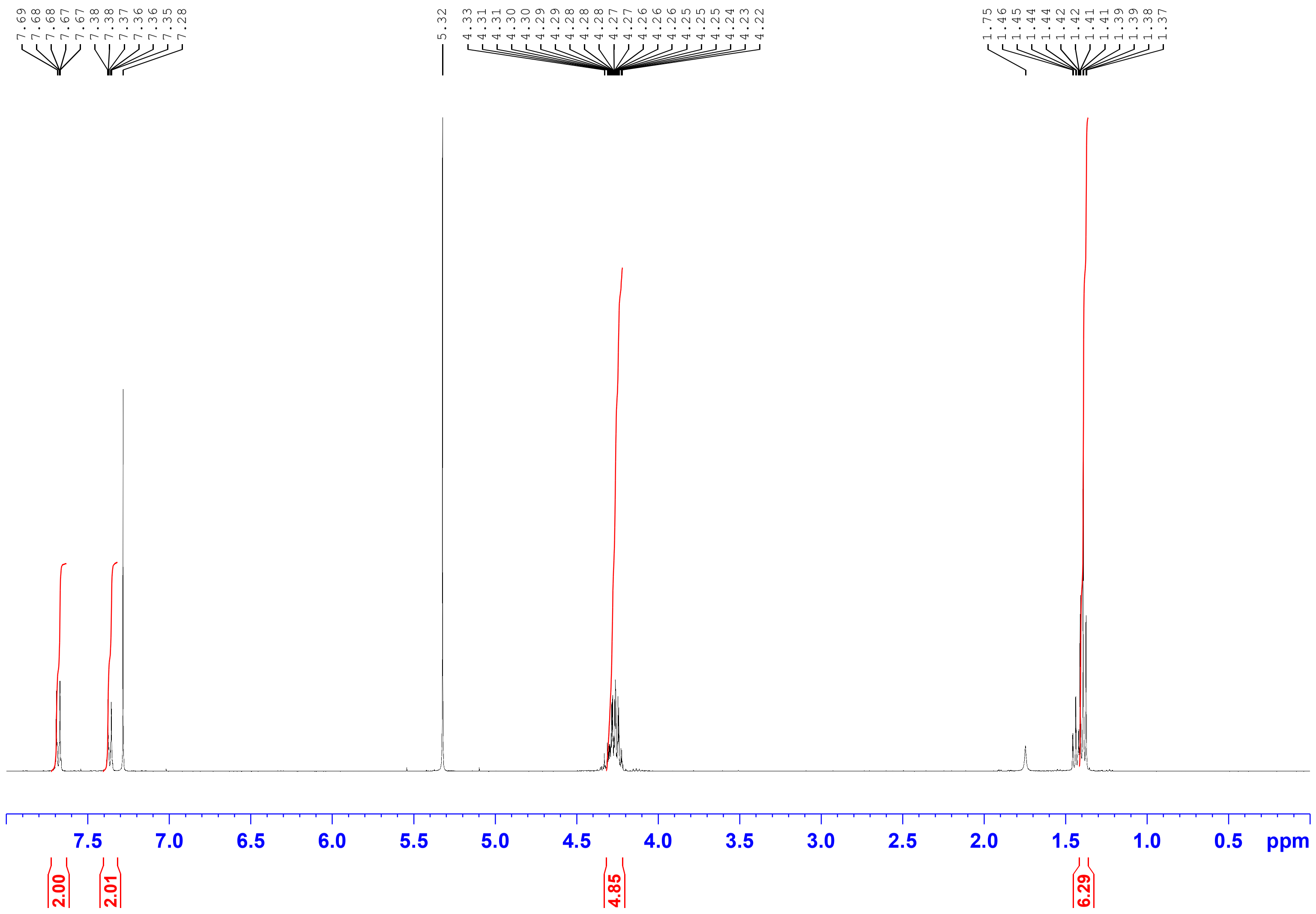

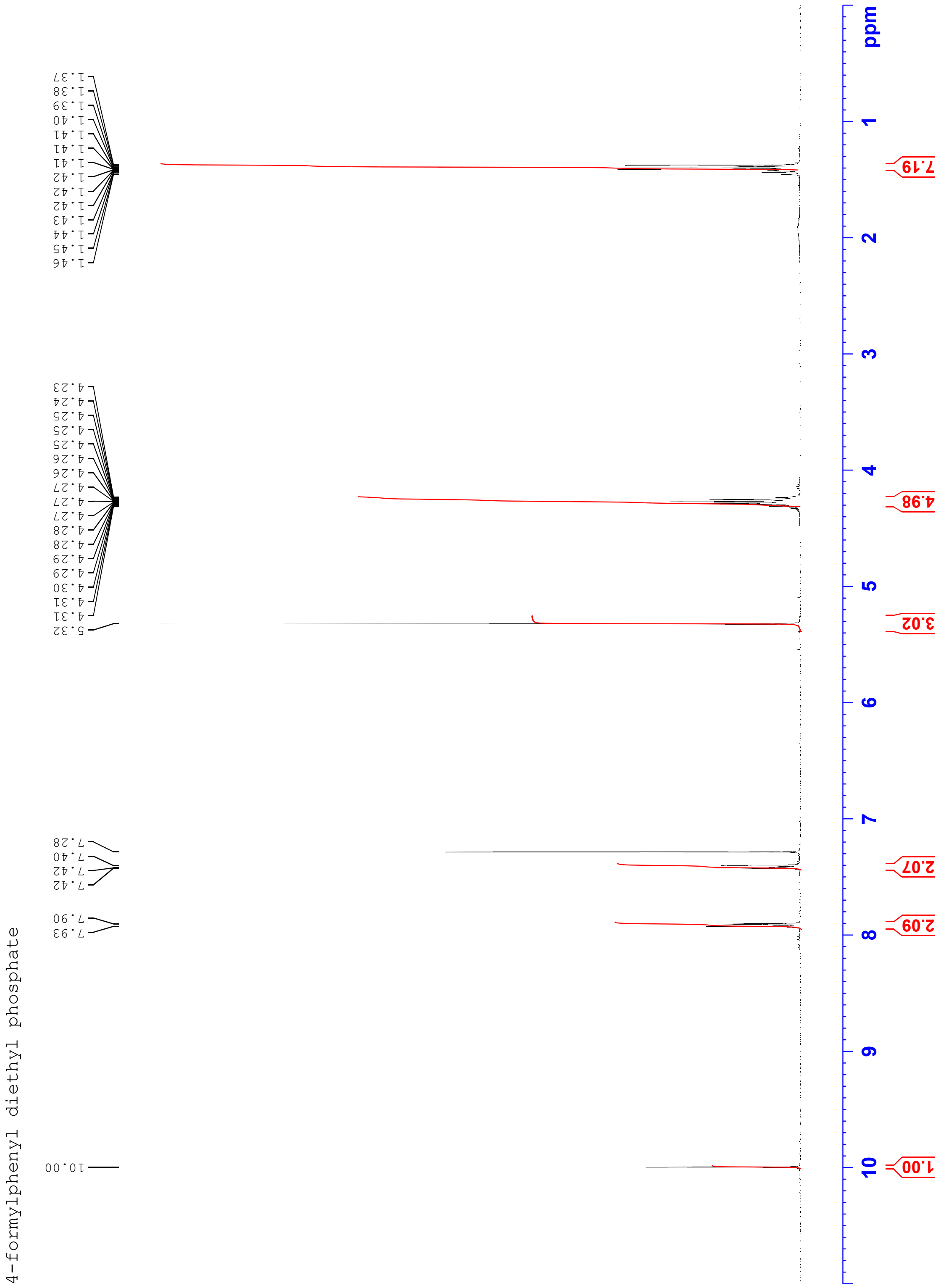
4-formylphenyl diethyl phosphate
1.37
1.38
1.39
1.40
1.41
1.41
1.41
1.42
1.42
1.42
1.43
1.44
1.45
1.46
4.23
4.24
4.25
4.25
4.25
4.26
4.26
4.27
4.27
4.27
4.28
4.28
4.29
4.29
4.30
4.31
4.31
5.32
7.28
7.40
7.42
7.42
7.90
7.93
10.00
7.19
4.98
3.02
2.07
2.09
1.00
ppm
1
2
3
4
5
6
7
8
9
10

Supplementary References:

(1) Fischlechner, M.; Schaerli, Y.; Mohamed, M. F.; Patil, S.; Abell, C.; Hollfelder, F. Evolution of Enzyme Catalysts Caged in Biomimetic Gel-Shell Beads. *Nat. Chem.* **2014**, *6* (9), 791–796. https://doi.org/10.1038/nchem.1996.

(2) Kille, S.; Acevedo-Rocha, C. G.; Parra, L. P.; Zhang, Z.-G.; Opperman, D. J.; Reetz, M. T.; Acevedo, J. P. Reducing Codon Redundancy and Screening Effort of Combinatorial Protein Libraries Created by Saturation Mutagenesis. *ACS Synth. Biol.* **2013**, *2* (2), 83–92. https://doi.org/10.1021/sb300037w.

(3) Engler, C.; Kandzia, R.; Marillonnet, S. A One Pot, One Step, Precision Cloning Method with High Throughput Capability. *PLOS ONE* **2008**, *3* (11), e3647. https://doi.org/10.1371/journal.pone.0003647.

(4) Acevedo-Rocha, C. G.; Reetz, M. T.; Nov, Y. Economical Analysis of Saturation Mutagenesis Experiments. *Sci. Rep.* **2015**, *5* (1). https://doi.org/10.1038/srep10654.

(5) Qin, D.; Xia, Y.; Whitesides, G. M. Soft Lithography for Micro- and Nanoscale Patterning. *Nat. Protoc.* **2010**, *5* (3), 491–502. https://doi.org/10.1038/nprot.2009.234.

(6) Neun, S.; Kaminski, T. S.; Hollfelder, F. Chapter Five - Single-Cell Activity Screening in Microfluidic Droplets. In *Methods in Enzymology*; Allbritton, N. L., Kovarik, M. L., Eds.; Enzyme Activity in Single Cells; Academic Press, 2019; Vol. 628, pp 95–112. https://doi.org/10.1016/bs.mie.2019.07.009.

(7) van Loo, B.; Heberlein, M.; Mair, P.; Zinchenko, A.; Schüürmann, J.; Eenink, B. D. G.; Holstein, J. M.; Dilkaute, C.; Jose, J.; Hollfelder, F.; Bornberg-Bauer, E. High-Throughput, Lysis-Free Screening for Sulfatase Activity Using Escherichia Coli Autodisplay in Microdroplets. *ACS Synth. Biol.* **2019**. https://doi.org/10.1021/acssynbio.9b00274.

(8) Zinchenko, A.; Devenish, S. R. A.; Kintses, B.; Colin, P.-Y.; Fischlechner, M.; Hollfelder, F. One in a Million: Flow Cytometric Sorting of Single Cell-Lysate Assays in Monodisperse Picolitre Double Emulsion Droplets for Directed Evolution. *Anal. Chem.* **2014**, *86* (5), 2526–2533. https://doi.org/10.1021/ac403585p.

(9) Suter-Crazzolara, C.; Unsicker, K. Improved Expression of Toxic Proteins in E. Coli. *BioTechniques* **1995**, *19* (2), 202–204.

(10) Saïda, F. Overview on the Expression of Toxic Gene Products in Escherichia Coli. *Curr. Protoc. Protein Sci.* **2007**, *Chapter 5*, Unit 5.19. https://doi.org/10.1002/0471140864.ps0519s50.

(11) Gasteiger, E. Protein Identification and Analysis Tools on the ExPASy Server.

(12) R Core Team. *R: A Language and Environment for Statistical Computing*; R Foundation for Statistical Computing: Vienna, Austria, 2017.

(13) Colin, P.-Y.; Kintses, B.; Gielen, F.; Miton, C. M.; Fischer, G.; Mohamed, M. F.; Hyvönen, M.; Morgavi, D. P.; Janssen, D. B.; Hollfelder, F. Ultrahigh-Throughput Discovery of Promiscuous Enzymes by Picodroplet Functional Metagenomics. *Nat. Commun.* **2015**, *6*, 10008. https://doi.org/10.1038/ncomms10008.

(14) Pathak, D.; Ollis, D. Refined Structure of Dienelactone Hydrolase at 1.8 Å. *J. Mol. Biol.* **1990**, *214* (2), 497–525. https://doi.org/10.1016/0022-2836(90)90196-S.

(15) Newcomb, R. D.; Campbell, P. M.; Ollis, D. L.; Cheah, E.; Russell, R. J.; Oakeshott, J. G. A Single Amino Acid Substitution Converts a Carboxylesterase to an Organophosphorus Hydrolase and Confers Insecticide Resistance on a Blowfly. *Proc. Natl. Acad. Sci.* **1997**, *94* (14), 7464–7468. https://doi.org/10.1073/pnas.94.14.7464.

(16) Campbell, P. M.; Newcomb, R. D.; Russell, R. J.; Oakeshott, J. G. Two Different Amino Acid Substitutions in the Ali-Esterase, E3, Confer Alternative Types of Organophosphorus Insecticide Resistance in the Sheep Blowfly, Lucilia Cuprina. *Insect Biochem. Mol. Biol.* **1998**, *28* (3), 139–150. https://doi.org/10.1016/S0965-1748(97)00109-4.

(17) Mabbitt, P. D.; Correy, G. J.; Meirelles, T.; Fraser, N. J.; Coote, M. L.; Jackson, C. J. Conformational Disorganization within the Active Site of a Recently Evolved Organophosphate Hydrolase Limits Its Catalytic Efficiency. *Biochemistry* **2016**, *55* (9), 1408–1417. https://doi.org/10.1021/acs.biochem.5b01322.

(18) Lockridge, O.; Blong, R. M.; Masson, P.; Froment, M.-T.; Millard, C. B.; Broomfield, C. A. A Single Amino Acid Substitution, Gly117His, Confers Phosphotriesterase (Organophosphorus Acid Anhydride Hydrolase) Activity on Human Butyrylcholinesterase. *Biochemistry* **1997**, *36* (4), 786–795. https://doi.org/10.1021/bi961412g.

(19) Zueva, I. V.; Lushchekina, S. V.; Daudé, D.; Chabrière, E.; Masson, P. Steady-State Kinetics of Enzyme-Catalyzed Hydrolysis of Echothiophate, a P–S Bonded Organophosphorus as Monitored by Spectrofluorimetry. *Molecules* **2020**, *25* (6), 1371. https://doi.org/10.3390/molecules25061371.

(20) Legler, P. M.; Boisvert, S. M.; Compton, J. R.; Millard, C. B. Development of Organophosphate Hydrolase Activity in a Bacterial Homolog of Human Cholinesterase. *Front. Chem.* **2014**, *2* (46), 1–15. https://doi.org/10.3389/fchem.2014.00046.

(21) Poyot, T.; Nachon, F.; Froment, M.-T.; Loiodice, M.; Wieseler, S.; Schopfer, L. M.; Lockridge, O.; Masson, P. Mutant of Bungarus Fasciatus Acetylcholinesterase with Low Affinity and Low Hydrolase Activity toward Organophosphorus Esters. *Biochim. Biophys. Acta BBA - Proteins Proteomics* **2006**, *1764* (9), 1470–1478. https://doi.org/10.1016/j.bbapap.2006.07.008.

(22) Cheah, E.; Austin, C.; Ashley, G. W.; Ollis, D. Substrate-Induced Activation of Dienelactone Hydrolase: An Enzyme with a Naturally Occurring Cys-His-Asp Triad. *Protein Eng. Des. Sel.* **1993**, *6* (6), 575–583. https://doi.org/10.1093/protein/6.6.575.

(23) Jumper, J.; Evans, R.; Pritzel, A.; Green, T.; Figurnov, M.; Ronneberger, O.; Tunyasuvunakool, K.; Bates, R.; Žídek, A.; Potapenko, A.; Bridgland, A.; Meyer, C.; Kohl, S. A. A.; Ballard, A. J.; Cowie, A.; Romera-Paredes, B.; Nikolov, S.; Jain, R.; Adler, J.; Back, T.; Petersen, S.; Reiman, D.; Clancy, E.; Zielinski, M.; Steinegger, M.; Pacholska, M.; Berghammer, T.; Bodenstein, S.; Silver, D.; Vinyals, O.; Senior, A. W.; Kavukcuoglu, K.; Kohli, P.; Hassabis, D. Highly Accurate Protein Structure Prediction with AlphaFold. *Nature* **2021**, *596* (7873), 583–589. https://doi.org/10.1038/s41586-021-03819-2.

(24) Mirdita, M.; Schütze, K.; Moriwaki, Y.; Heo, L.; Ovchinnikov, S.; Steinegger, M. ColabFold: Making Protein Folding Accessible to All. *Nat. Methods* **2022**, *19* (6), 679–682. https://doi.org/10.1038/s41592-022-01488-1.

(25) Khersonsky, O.; Tawfik, D. S. Structure−Reactivity Studies of Serum Paraoxonase PON1 Suggest That Its Native Activity Is Lactonase. *Biochemistry* **2005**, *44* (16), 6371–6382. https://doi.org/10.1021/bi047440d.

(26) Yang, G.; Hong, N.; Baier, F.; Jackson, C. J.; Tokuriki, N. Conformational Tinkering Drives Evolution of a Promiscuous Activity through Indirect Mutational Effects. *Biochemistry* **2016**, *55* (32), 4583–4593. https://doi.org/10.1021/acs.biochem.6b00561.

(27) Aharoni, A.; Gaidukov, L.; Yagur, S.; Toker, L.; Silman, I.; Tawfik, D. S. Directed Evolution of Mammalian Paraoxonases PON1 and PON3 for Bacterial Expression and Catalytic Specialization. *Proc. Natl. Acad. Sci. U. S. A.* **2004**, *101* (2), 482–487. https://doi.org/10.1073/pnas.2536901100.

(28) Aharoni, A.; Gaidukov, L.; Khersonsky, O.; Gould, S. M.; Roodveldt, C.; Tawfik, D. S. The "evolvability" of Promiscuous Protein Functions. *Nat. Genet.* **2005**, *37* (1), 73–76. https://doi.org/10.1038/ng1482.

(29) Meier, M. M.; Rajendran, C.; Malisi, C.; Fox, N. G.; Xu, C.; Schlee, S.; Barondeau, D. P.; Höcker, B.; Sterner, R.; Raushel, F. M. Molecular Engineering of Organophosphate Hydrolysis Activity from a Weak Promiscuous Lactonase Template. *J. Am. Chem. Soc.* **2013**, *135* (31), 11670–11677. https://doi.org/10.1021/ja405911h.

(30) Griffiths, A. D. Directed Evolution of an Extremely Fast Phosphotriesterase by in Vitro Compartmentalization. *EMBO J.* **2003**, *22* (1), 24–35. https://doi.org/10.1093/emboj/cdg014.

(31) Hawwa, R.; Larsen, S. D.; Ratia, K.; Mesecar, A. D. Structure-Based and Random Mutagenesis Approaches Increase the Organophosphate-Degrading Activity of a Phosphotriesterase Homologue from Deinococcus Radiodurans. *J. Mol. Biol.* **2009**, *393* (1), 36–57. https://doi.org/10.1016/j.jmb.2009.06.083.

(32) Zhang, Y.; An, J.; Ye, W.; Yang, G.; Qian, Z.-G.; Chen, H.-F.; Cui, L.; Feng, Y. Enhancing the Promiscuous Phosphotriesterase Activity of a Thermostable Lactonase (GkaP) for the Efficient Degradation of Organophosphate Pesticides. *Appl. Environ. Microbiol.* **2012**, *78* (18), 6647–6655. https://doi.org/10.1128/AEM.01122-12.

(33) Hoque, M. A.; Zhang, Y.; Chen, L.; Yang, G.; Khatun, M. A.; Chen, H.; Hao, L.; Feng, Y. Stepwise Loop Insertion Strategy for Active Site Remodeling to Generate Novel Enzyme Functions. *ACS Chem. Biol.* **2017**, *12* (5), 1188–1193. https://doi.org/10.1021/acschembio.7b00018.

(34) Jackson, C. J.; Weir, K.; Herlt, A.; Khurana, J.; Sutherland, T. D.; Horne, I.; Easton, C.; Russell, R. J.; Scott, C.; Oakeshott, J. G. Structure-Based Rational Design of a Phosphotriesterase. *Appl. Environ. Microbiol.* **2009**, *75* (15), 5153–5156. https://doi.org/10.1128/AEM.00629-09.

(35) Yang, Q.; Li, Y.; Yang, J.-D.; Liu, Y.; Zhang, L.; Luo, S.; Cheng, J.-P. Holistic Prediction of the PKa in Diverse Solvents Based on a Machine-Learning Approach. *Angew. Chem. Int. Ed.* **2020**, *59* (43), 19282–19291. https://doi.org/10.1002/anie.202008528.
